# A foundation model to predict and capture human cognition

**DOI:** 10.1038/s41586-025-09215-4

**Published:** 2025-07-02

**Authors:** Marcel Binz, Elif Akata, Matthias Bethge, Franziska Brändle, Fred Callaway, Julian Coda-Forno, Peter Dayan, Can Demircan, Maria K. Eckstein, Noémi Éltető, Thomas L. Griffiths, Susanne Haridi, Akshay K. Jagadish, Li Ji-An, Alexander Kipnis, Sreejan Kumar, Tobias Ludwig, Marvin Mathony, Marcelo Mattar, Alireza Modirshanechi, Surabhi S. Nath, Joshua C. Peterson, Milena Rmus, Evan M. Russek, Tankred Saanum, Johannes A. Schubert, Luca M. Schulze Buschoff, Nishad Singhi, Xin Sui, Mirko Thalmann, Fabian J. Theis, Vuong Truong, Vishaal Udandarao, Konstantinos Voudouris, Robert Wilson, Kristin Witte, Shuchen Wu, Dirk U. Wulff, Huadong Xiong, Eric Schulz

**Affiliations:** 1 Institute for Human-Centered AI, Helmholtz Center, Munich, Germany; 2 University of Tübingen, Tübingen, Germany; 3 University of Oxford, Oxford, UK; 4 Max Planck Institute for Biological Cybernetics, Tübingen, Germany; 5 New York University, New York, NY USA; 6 Google DeepMind, London, UK; 7 Princeton University, Princeton, NJ USA; 8 Max Planck School of Cognition, Leipzig, Germany; 9 University of California, San Diego, San Diego, CA USA; 10 Boston University, Boston, MA USA; 11 TU Darmstadt, Darmstadt, Germany; 12 Institute of Computational Biology, Helmholtz Center, Munich, Germany; 13 TUM School of Computation, Information and Technology, Technical University of Munich, Munich, Germany; 14 TUM School of Life Sciences, Technical University of Munich, Munich, Germany; 15 University of Cambridge, Cambridge, UK; 16 Georgia Institute of Technology, Atlanta, GA USA; 17 University of Basel, Basel, Switzerland; 18 Max Planck Institute for Human Development, Berlin, Germany

**Keywords:** Human behaviour, Computational science, Neuroscience

## Abstract

Establishing a unified theory of cognition has been an important goal in psychology^1,2^. A first step towards such a theory is to create a computational model that can predict human behaviour in a wide range of settings. Here we introduce Centaur, a computational model that can predict and simulate human behaviour in any experiment expressible in natural language. We derived Centaur by fine-tuning a state-of-the-art language model on a large-scale dataset called Psych-101. Psych-101 has an unprecedented scale, covering trial-by-trial data from more than 60,000 participants performing in excess of 10,000,000 choices in 160 experiments. Centaur not only captures the behaviour of held-out participants better than existing cognitive models, but it also generalizes to previously unseen cover stories, structural task modifications and entirely new domains. Furthermore, the model’s internal representations become more aligned with human neural activity after fine-tuning. Taken together, our results demonstrate that it is possible to discover computational models that capture human behaviour across a wide range of domains. We believe that such models provide tremendous potential for guiding the development of cognitive theories, and we present a case study to demonstrate this.

## Abstract (web summary)

A computational model called Centaur, developed by fine-tuning a language model on a huge dataset called Psych-101, can predict and simulate human nature in experiments expressible in natural language, even in previously unseen situations.

## Main

The human mind is remarkably general^3^. Not only do we routinely make mundane decisions, such as choosing a breakfast cereal or selecting an outfit, but we also tackle complex challenges, such as figuring out how to cure cancer or explore outer space. We learn skills from only a few demonstrations^4^, reason causally^5^ and fuel our actions through curiosity^6^. Whether we are climbing mountains, playing video games, or creating captivating art, our versatility defines what it means to be human.

By contrast, most contemporary computational models, whether in machine learning or the cognitive sciences, are domain specific. They are designed to excel at one particular problem and only that problem. Consider, for instance, AlphaGo, which is a computer system created by Google DeepMind to master the game of Go^7^. The system can play this particular game at an impressive level, but it cannot do much beyond that. A similar pattern can be observed in the cognitive sciences. For instance, prospect theory, which is one of the most influential accounts of human cognition, offers valuable insights into how people make choices^8^, but it tells us nothing about how we learn, plan or explore.

If we want to understand the human mind in its entirety, we must move from domain-specific theories to an integrated one. The importance of such a unified approach has already been recognized by the pioneers of our field. For example, in 1990, it was stated that “unified theories of cognition are the only way to bring [our] wonderful, increasing fund of knowledge under intellectual control”^2^. How can we make meaningful progress towards such theories?

**Fig. 1:**
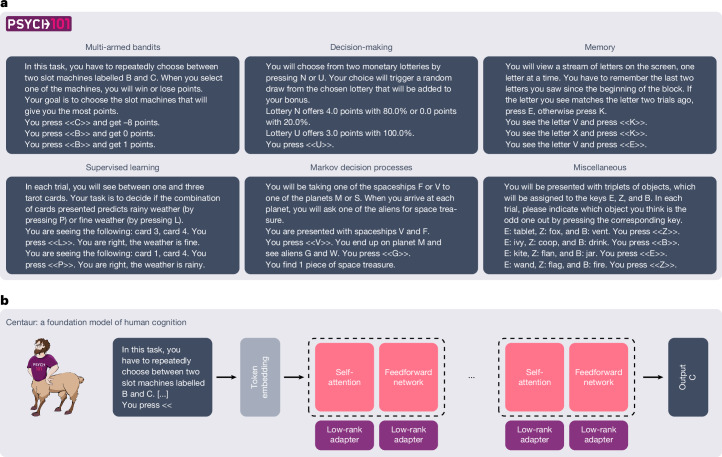
Overview of Psych-101 and Centaur. **a**, Psych-101 comprises trial-by-trial data from 160 psychological experiments with 60,092 participants making 10,681,650 choices in total and involving 253,597,411 text tokens. It contains domains such as multi-armed bandits, decision-making, memory, supervised learning, Markov decision processes and others (the examples shown have been stylized and abbreviated for readability). **b**, Centaur is a foundation of model human cognition that is obtained by adding low-rank adapters to a state-of-the-art language model and fine-tuning it on Psych-101.

**Extended Data Fig. 1:**
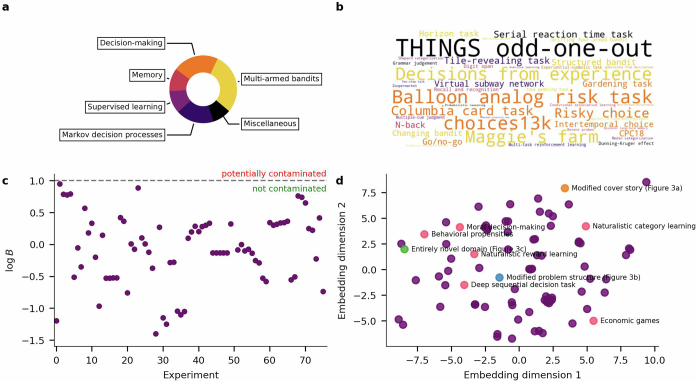
Psych-101. **a**, Proportion of domains included in Psych-101. **b**, Word cloud of experimental paradigms included in Psych-101. **c**, We performed a data contamination analysis using the LogProber method^67^ for every experimental paradigm in Psych-101. LogProber fits a two-parameter exponential model to the cumulative log-likelihood of each sequence being checked for contamination. High acceleration (log B) suggests that a prompt is memorized from the pretraining data. Following the results presented in the original work^67^, we set a threshold for possible contamination to log B ≥ 1. This analysis indicated no evidence of contamination. **d**, Two-dimensional embedding of the experiments used in this paper. To obtain this embedding, we took the corresponding natural language prompts up to the point of the first human choice, extracted a vector-based representation for them using ModernBERT^68^, and finally projected these representations onto two dimensions using multidimensional scaling. Purple dots correspond to experiments from Psych-101, whereas the colored dots correspond to the indicated evaluation experiment.

An important step towards a unified theory of cognition is to build a computational model that can predict and simulate human behaviour in any domain^2,9^. In this paper, we take up this challenge and introduce Centaur—a foundation model of human cognition^10^. Centaur was designed in a data-driven manner by fine-tuning a state-of-the-art large language model^11^ on a large corpus of human behaviour. For this purpose, we curated a large-scale dataset called Psych-101, which covers trial-by-trial data from 160 psychological experiments (see Methods, ‘Data collection’ and Extended Data Fig. 1). We transcribed each of these experiments into natural language, which provides a common format for expressing vastly different experimental paradigms^12,13^. The resulting dataset has an unprecedented scale, containing more than 10,000,000 human choices and including many canonical studies from domains such as multi-armed bandits, decision-making, memory, supervised learning, Markov decision processes and more (see Fig. 1a for an overview and examples).

We subjected Centaur to a series of rigorous tests and demonstrate that it captures human behaviour at several levels of generalization. First, we show that Centaur predicts behaviour of held-out participants (those who are not part of the training data) better than existing cognitive models in almost every single experiment. We then demonstrate that its ability to capture human behaviour also generalizes to held-out experiments. In this context, we find that Centaur accurately predicts human behaviour under modified cover stories, problem structures and even in entirely new domains. Finally, we show that Centaur’s internal representations become more human aligned, even though it was never explicitly trained to capture human neural activity.

Taken together, our results demonstrate that it is possible to discover computational models that capture human behaviour across a wide range of domains. We think that such a predictive model offers many direct opportunities to obtain a better understanding of the human mind^14,15^ and we present a case study that demonstrates this potential.

## Model overview

We built Centaur on top of the open-source language model Llama 3.1 70B, a state-of-the-art model pretrained by Meta AI (hereafter, we refer to this model simply as Llama)^11^. Having a large language model as the backbone allowed us to rely on the vast amounts of knowledge that is present in these models. The training process involved fine-tuning on Psych-101 using a parameter-efficient fine-tuning technique known as quantized low-rank adaptation (QLoRA)^16^. QLoRA relies on a frozen four-bit quantized language model as a base model. Although the parameters of the base model are left unchanged, it adds low-rank adapters, which contain only a few additional, trainable parameters (typically represented in a half-precision floating-point format). In our case, we added low-rank adapters of rank *r* = 8 to all non-embedding layers (that is, all linear layers of the self-attention mechanisms and the feedforward networks), as illustrated in Fig. 1b. With these settings, the newly added parameters amount to 0.15% of the base model’s parameters. We then trained the model for one epoch on the entire dataset using a standard cross-entropy loss. We masked out the loss for all tokens that do not correspond to human responses, thereby ensuring that the model focuses on capturing human behaviour and not on completing experimental instructions. The entire training process took approximately five days on an A100 80GB GPU (Methods, ‘Fine-tuning procedure’).

## Centaur captures human behaviour

**Fig. 2:**
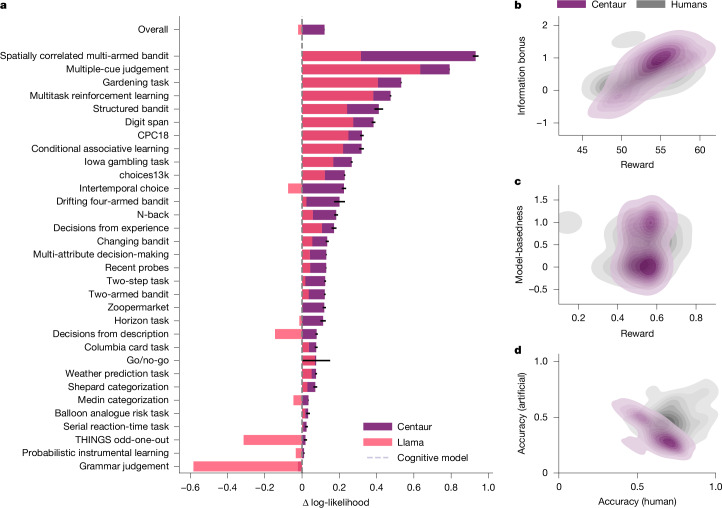
Goodness-of-fit on Psych-101. **a**, Difference in log-likelihood of Centaur and Llama relative to a domain-specific cognitive model for each experiment. A value of zero corresponds to the goodness-of-fit of the domain-specific cognitive model and a value above zero indicates improved goodness-of-fit to human responses. Log-likelihoods are averaged over responses (*n* = 992,867). Error bars correspond to the standard error of the mean. Centaur outperforms both Llama and a collection of domain-specific cognitive models in almost every experiment (one-sided *t*-tests: *t*(1,985,732) = −144.22, *P* ≤ 0.0001; *t*(1,985,732) = −127.58, *P* ≤ 0.0001, respectively). We only included experiments for which we have implemented a domain-specific cognitive model in this graphic and merged different studies using the same paradigm. Extended Data Table 1 contains numerical results for all experiments. **b**, Model simulations on the horizon task. The plot shows the probability densities over reward and an information bonus parameter for both people and simulated runs of Centaur. **c**, Model simulations on the two-step task. The plot shows the probability densities over reward and a parameter indicating how model-based learning was for both people and simulated runs of Centaur. **d**, Model simulations on a social prediction game. The plot shows the probability densities over accuracies of predicting human strategies and strategies of an artificial agent, with matched statistics for both people and simulated runs of Centaur.

We evaluated Centaur on different types of held-out data to demonstrate that it robustly captures human behaviour. In our first analysis, we tested whether it could predict the behaviour of participants who were not part of the training data. For this, we split each transcribed experiment into two parts and used 90% of participants for training and retained 10% for testing. We measured goodness-of-fit to human choices using negative log-likelihoods averaged across responses (Methods, ‘Evaluation metric’). Figure 2a presents the results of this analysis, comparing Centaur with the base model without fine-tuning and a collection of domain-specific models that represent the state-of-the-art in the cognitive-science literature (Extended Data Table 1). Although there was substantial variance in predictability across experiments (Centaur, 0.49; Llama, 0.47), fine-tuning always improved goodness-of-fit. The average difference in log-likelihoods across experiments after fine-tuning was 0.14 (Centaur negative log-likelihood, 0.44; Llama negative log-likelihood, 0.58; one-sided *t*-test: *t*(1,985,732) = −144.22, *P* ≤ 0.0001; Cohen’s *d*, 0.20).

**Extended Data Fig. 2:**
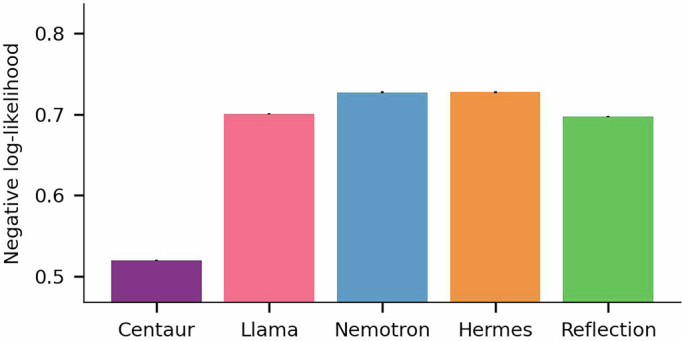
Negative log-likelihoods of Centaur and alternative Llama variants on Psych-101. To rule out the hypothesis that finetuning on any data aligns a model with human behavior, we compared Centaur to various Llama variants finetuned for other purposes (i.e. non-cognitive tasks). Nemotron^69^ is finetuned for instruction-following. Hermes^70^ is finetuned for various purposes, including agentic capabilities, roleplaying, reasoning, multi-turn conversation, and long context coherence. Reflection is finetuned for reasoning. None of the Llama variants captures human behavior better than the base model, ruling out the hypothesis that finetuning generally leads to models that are better at predicting human behavior. Error bars correspond to the standard error of the mean, taken over responses.

**Extended Data Fig. 3:**
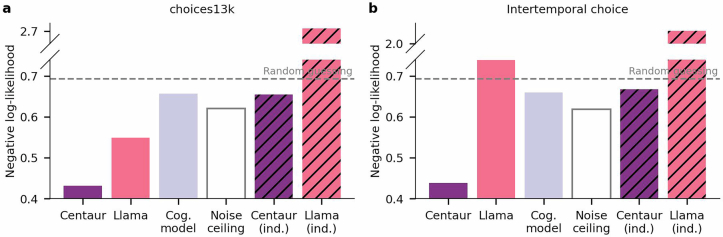
Noise ceiling analysis. We conducted a noise ceiling analysis to better understand the capabilities of Centaur. It is not straightforward to estimate the noise ceiling for experiments with sequential dependencies, which includes the majority of Psych-101. Hence, we focused on two experiments for which such an analysis is possible: **a**, the choices13k data set^18^ and **b**, an intertemporal choice experiment^52^. In both cases, we found that Centaur substantially exceeds the estimated noise ceiling. This is possible because Centaur can pick up on context-dependent patterns that are not captured by standard noise ceiling analyses. Therefore, we have performed an additional analysis testing how well Centaur can predict human responses if we prompt it to predict each response independently. We use the suffix “ind.” to indicate this way of prompting the model. Centaur still matches the performance of domain-specific cognitive models when context-independent prompts are used, amounting to roughly half of the estimated noise ceiling.

Furthermore, we compared Centaur with the previously mentioned collection of domain-specific cognitive models. These models include, among others, the generalized context model^17^, a prospect theory model^18^ and various reinforcement learning models^19,20^ (Methods, ‘Domain-specific cognitive models’). We observed that Centaur outperforms domain-specific cognitive models in all but one experiment. The average difference in predicting human behaviour to the domain-specific cognitive models was 0.13 (cognitive models, negative log-likelihood, 0.56; one-sided *t*-test: *t*(1,985,732) = −127.58, *P* ≤ 0.0001; Cohen’s *d*, 0.18). Extended Data Figs. 2 and 3 contain more comparisons to models fine-tuned on non-behavioural data and a noise-ceiling analysis.

The previous analyses have focused on predicting human responses conditioned on previously executed behaviour. We may ask whether Centaur can also generate human-like behaviour when simulated in an open-loop fashion (that is, when feeding its own responses back into the model). This setting arguably provides a much stronger test of the model’s capabilities and is sometimes also referred to as model falsification^21^. To check whether Centaur survives this test, we ran open-loop simulations in three different experimental paradigms and inspected the distributions of statistics that resulted from these simulations. First, we simulated Centaur on the horizon-task paradigm, a two-armed bandit task used to detect different types of exploration strategies^20^. We found that Centaur (mean = 54.12, s.d. = 2.89) achieved a performance comparable to human participants (mean = 52.78, s.d. = 2.90), which was supported by an equivalence test using the two one-sided *t*-tests procedure with a ±3-point margin (*P* = 0.02). Centaur also engaged in a similar level of uncertainty-guided directed exploration (Fig. 2b), a pattern that is notably absent in many contemporary language models^12^.

We also observed that Centaur does not merely capture the behaviour of the average participant, but rather the distribution over trajectories produced by the entire population. For example, in the two-step task (a well-known paradigm used to tease apart model-free and model-based reinforcement learning^19^), Centaur, just like human subjects, produced trajectories in which learning is purely model-free, purely model-based and mixtures thereof (as the bimodal distribution in Fig. 2c shows).

Finally, we verified that Centaur fails at predicting non-human behaviour. For this, we considered a study that required participants to predict either human responses or responses of an artificial agent with matched statistics in four canonical economic games^22^. Mirroring the results of the original human study, Centaur accurately predicted human responses (64% accuracy) but struggled to predict artificial responses (35% accuracy; one-sided *t*-test: *t*(230) = 20.32, *P* ≤ 0.0001; Fig. 2d). Taken together, these results demonstrate that Centaur exhibits human-like characteristics across various settings, confirming that it can generate meaningful open-loop behaviour.

## Probing generalization abilities

So far, we have shown that Centaur generalizes to previously unseen participants performing experiments that were part of the training data. A true foundation model of human cognition, however, must also capture behaviour in any arbitrary experiment, even if that experiment was not part of the training data. To probe whether Centaur has this ability, we exposed it to a series of increasingly complex out-of-distribution evaluations.

**Fig. 3:**
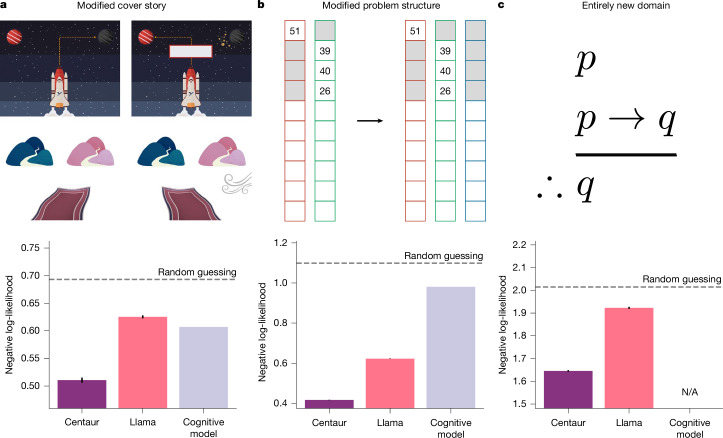
Evaluation in different held-out settings. **a**, Negative log-likelihoods averaged over responses (*n* = 9,702) for the two-step task with a modified cover story^23^. **b**, Negative log-likelihoods averaged over responses (*n* = 510,154) for a three-armed bandit experiment^25^. **c**, Negative log-likelihoods averaged over responses (*n* = 99,204) for an experiment probing logical reasoning^26^ with items based on the Law School Admission Test (LSAT). Centaur outperforms both Llama and domain-specific cognitive models when faced with modified cover stories, problem structures and entirely new domains. N/A, not applicable. Error bars show the s.e.m. The image in **a** is reproduced from ref. ^23^, Springer Nature Limited. The image in **c** is reproduced from Wikipedia.org.

First, we investigated whether Centaur is robust in the face of changes to the cover story. For this analysis, we relied on data collected in ref. ^23^, which used the aforementioned two-step task. In addition to the canonical cover story (spaceships travelling to foreign planets in search of treasures), the study introduced a new cover story involving magical carpets. Importantly, Psych-101 includes experiments using the canonical spaceship cover story^24^ but no experiments with the magic-carpet cover story. Even so, we found that Centaur captured human behaviour in the magic-carpet experiment of ref. ^23^ (Fig. 3a). As in our previous analysis, we observed an improvement after fine-tuning, as well as a favourable goodness-of-fit when compared with a domain-specific cognitive model (Centaur negative log-likelihood, 0.51; Llama negative log-likelihood, 0.63; cognitive model negative log-likelihood, 0.61; one-sided *t*-test comparing Centaur with Llama: *t*(9,701) = −24.7, *P* ≤ 0.0001; one-sided *t*-test comparing Centaur with the domain-specific cognitive model: *t*(9,701) = −20.7, *P* ≤ 0.0001; the domain-specific cognitive model used in this analysis was a hybrid model that combined model-based and model-free reinforcement learning)^19^.

In a second out-of-distribution evaluation, we probed whether Centaur is robust to modifications in task structure. To test this, we exposed it to a paradigm known as Maggie’s farm^25^. Maggie’s farm extends the horizon task paradigm by adding a third option. Psych-101 encompasses several two-armed bandit experiments (including the horizon task) but not Maggie’s farm or any other three-armed bandit experiments (it does, however, contain multi-armed bandit experiments with more than three options to choose between). Thus, this analysis provides a test of Centaur’s robustness to structural task modifications. We found that Centaur captured human behaviour on Maggie’s farm, as shown in Fig. 3b. We again observed a benefit of fine-tuning, as well as a favourable goodness-of-fit compared with a domain-specific cognitive model, which did not generalize well to this setting (Centaur negative log-likelihood, 0.42; Llama negative log-likelihood, 0.62; cognitive model negative log-likelihood, 0.98; one-sided *t*-test comparing Centaur with Llama: *t*(510,153) = −204.2, *P* ≤ 0.0001; one-sided *t*-test comparing Centaur with the domain-specific cognitive model: *t*(510,153) = −559.8, *P* ≤ 0.0001).

Finally, we investigated whether Centaur could capture human behaviour even in entirely new domains. In this context, we considered a study investigating logical reasoning^26^. Although Psych-101 includes probabilistic and causal reasoning problems, we purposefully excluded any studies involving logical reasoning. As in the previous analyses, there was again a positive effect of fine-tuning (Centaur negative log-likelihood, 1.65; Llama negative log-likelihood, 1.92; one-sided *t*-test: *t*(198,406) = −50.39, *P* ≤ 0.0001; Cohen’s *d*, 0.23; Fig. 3c). Note that we did not compare with any domain-specific cognitive model in this setting, because it is unclear how to construct a model that would make any meaningful transfer from training data that does not include any related problems.

**Extended Data Fig. 4:**
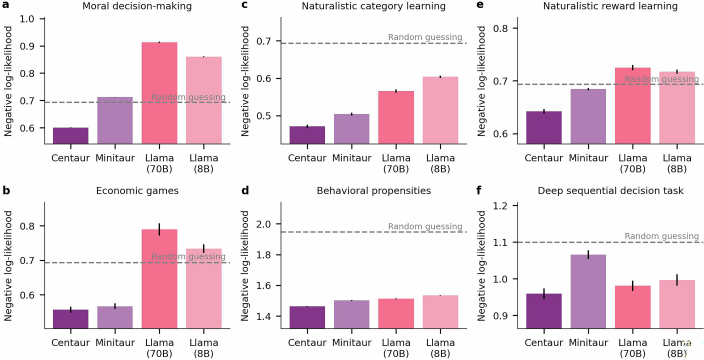
Further out-of-distribution evaluations. Each subplot shows negative log-likelihoods for a different experiment. None of these paradigms were included in Psych-101, hence they provide a stress test for a model’s generalization capabilities. Centaur robustly captured human behavior in all of these settings, while smaller and non-finetuned models did not do so consistently. Error bars correspond to the standard error of the mean, taken over responses. We state one-sided t-tests comparing the negative log-likelihoods of Centaur to those of Llama in brackets. **a**, Negative log-likelihoods on moral decision-making^27^ (t(181388) = −103.54, p ≤ 0.0001). **b**, Negative log-likelihoods on economic games^1^ (t(7798) = −11.69, p ≤ 0.0001). **c**, Negative log-likelihoods on naturalistic category learning^1^ (t(21838) = −14.05, p ≤ 0.0001). **d**, Negative log-likelihoods on behavioral propensities^30^ (t(156230) = −11.06, p ≤ 0.0001). **e**, Negative log-likelihoods on naturalistic reward learning^1^ (t(9838) = −12.63, p ≤ 0.0001). **f**, Negative log-likelihoods on a deep sequential decision task^31^ (t(6092) = −1.06, p = 0.144).

We consolidated these results by analysing Centaur on six more out-of-distribution experimental paradigms that were not part of the training data in any shape or form (including moral decision-making^27^, economic games^28^, naturalistic category and reward learning^29^, behavioural propensities^30^ and a deep sequential decision task^31^). Centaur robustly captured human behaviour in all these settings, whereas smaller and non-fine-tuned models did not do so consistently (Extended Data Fig. 4).

As well as analysing human choice data, we also examined whether Centaur could predict human response times. Hick’s law^32^ indicates that individual response times are a linear function of response entropies. Therefore, we extracted nearly 4,000,000 response times for a subset of experiments in Psych-101 and fitted three linear mixed effects models, each predicting log-transformed response times based on log-transformed response entropies derived from a different computational model. We found that the response entropies derived from Centaur captured a larger proportion of the variance in response times (conditional *R*^2^, 0.87) than those derived from Llama (conditional *R*^2^, 0.75, log[BF_Centaur, Llama_] = 53,773.5) and the cognitive models (conditional *R*^2^, 0.77, log[BF_Centaur, cognitive models_] = 14,995.5), thereby highlighting Centaur’s ability to predict measures beyond pure choice data.

**Fig. 4:**
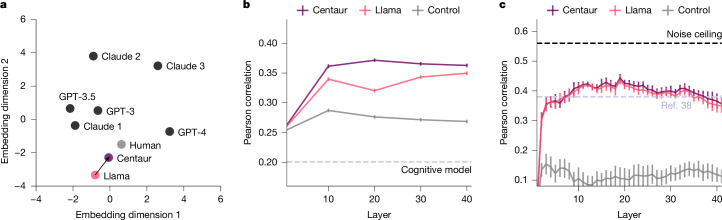
Human alignment. **a**, Multidimensional scaling embedding of the ten behavioural metrics in CogBench^33^ for different models. **b**, Pearson correlation coefficients indicating how well human neural activity in the two-step task^37^ can be decoded using Centaur’s internal representations extracted from different layers. **c**, Pearson correlation coefficients indicating how well human neural activity in a sentence-reading task^38^ can be decoded using Centaur’s internal representations extracted from different layers. Control refers to a model that used representations extracted from a randomly initialized transformer model with matched architecture.

**Extended Data Fig. 5:**
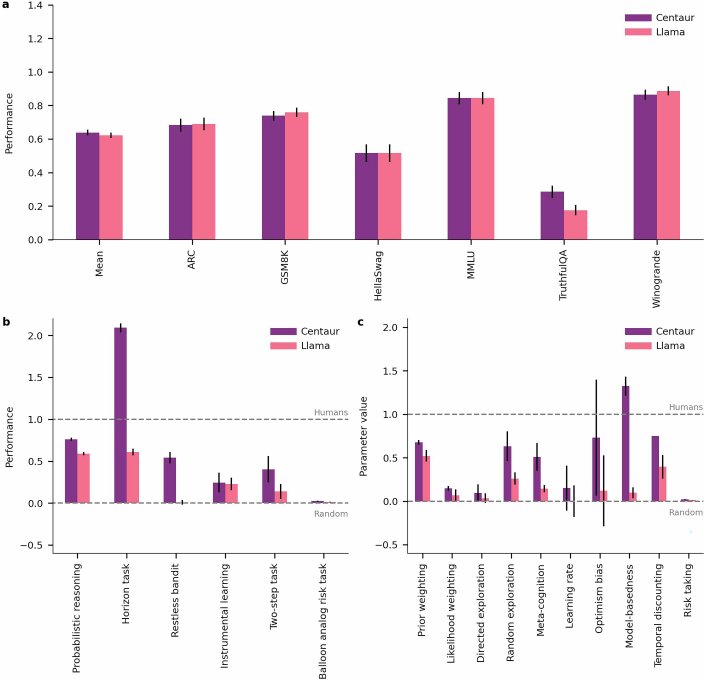
metabench and CogBench results. **a**, Results for metabench^34^, a sparse benchmark containing several canonical benchmarks from the machine learning literature. We find that Centaur maintains the level of performance of Llama, indicating that finetuning on human behavior did not lead to deterioration in other tasks (ARC: z = −0.126, p = 0.9, GSM8K: z = −0.529, p = 0.597, HellaSwag: z = 0.0, p = 1.0, MMLU: z = 0.0, p = 1.0, Winogrande: z = −0.556, p = 0.578). Performance on TruthfulQA^71^ – which measures how models mimic human falsehoods – even improved significantly with finetuning (z = 2.312, p = 0.021; all z-test were two-sided). **b**, Performance-based metrics from CogBench^33^, a benchmark that includes ten behavioral metrics derived from seven cognitive psychology experiments. We find that – relative to Llama – Centaur’s performance improves in all experiments (Probabilistic reasoning: z = 6.371, p ≤ 0.0001, Horizon task: z = 22.176, p ≤ 0.0001, Restless bandit: z = 7.317, p ≤ 0.0001, Instrumental learning: z = 0.126, p = 0.45, Two-step task: z = 1.458, p = 0.072, Balloon analog risk task: z = 1.496, p = 0.067; all z-test were one-sided). **c**, Behavioral metrics from CogBench. We observe that Centaur becomes more similar to human subjects in all ten behavioral metrics (Prior weighting: z = 2.176, p = 0.015, Likelihood weighting: z = 1.131, p = 0.129, Directed exploration: z = 0.525, p = 0.3, Random exploration: z = 2.014, p = 0.022, Meta-cognition: z = 2.206, p = 0.014, Learning rate: z = 0.477, p = 0.317, Optimism bias: z = 0.78, p = 0.218, Model-basedness: z = 9.608, p ≤ 0.0001, Temporal discounting: z = 2.594, p = 0.005, Risk taking: z = 1.612, p = 0.053; all z-test were one-sided).

To demonstrate that the model does not degrade on problems it was pretrained for, we furthermore verified it on a collection of benchmarks from the machine-learning literature^33,34^. We found that Centaur remains stable in performance-based benchmarks, even improving over the base model in some of them^34^ (Extended Data Fig. 5a,b). Finally, in benchmarks that measure human alignment, we observed a shift towards human-like characteristics (Extended Data Fig. 5c). Figure 4a depicts this improved alignment on a low-dimensional embedding derived from ten behavioural metrics in CogBench, a benchmark to test the cognitive abilities of large language models^33^.

## Alignment to human neural activity

**Extended Data Fig. 6:**
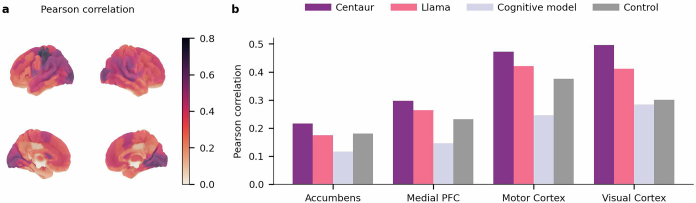
Finegrained neural alignment results in the two-step task. **a**, Pearson correlation coefficients between the predicted activity from Centaur’s representations and the BOLD data shown on a surface brain (image created with nilearn^66^). Centaur achieves the most accurate predictions in the left motor cortex. As participants performed the task with their right hand in the scanner, this effect may be explained by Centaur’s strong performance in predicting choices. **b**, Predictive performance of Centaur’s representations against alternatives for ROIs that have been identified as behaviorally relevant in previous work. Cortical scores are averaged over the corresponding bilateral parcels in the Schaefer atlas. The accumbens is defined based on the Harvard-Oxford atlas. Pearson correlation coefficients are shown for layer 20 but exhibit a similar pattern across all layers. Centaur outperformed Llama and the cognitive model in predicting activity in accumbens, the ROI from the original study that showed a reward prediction error effect^19,37^. We found a similar pattern in the medial PFC, another region that showed an effect in the original article^37^, as well as in the sensory and motor cortices.

Despite being trained to match only human behaviour, we also wondered whether Centaur’s internal representations become more aligned with human neural activity. To check whether this is the case, we conducted two analyses in which we predicted human neural activity using the model’s internal representations^35,36^. We first conducted a whole-brain analysis in which we predicted functional magnetic resonance imaging (fMRI) measurements of people performing the two-step task^37^. For this, we relied on data collected in a previous study^37^ involving 94 participants each making 300 choices. Participants were tested on either the magic-carpet cover story (which we had already used in one of our earlier generalization analyses) or an abstract cover story. Neither of these two cover stories was part of Centaur’s training data. We extracted recordings from models’ residual stream before each choice and after feedback. We then aggregated human neural activity in each region and regressed the aggregated activity on Centaur’s internal representations. This procedure was then repeated separately for each participant and region (Methods, ‘Neural alignment’). Figure 4b shows the resulting Pearson correlation coefficients across layers for both Centaur and Llama averaged over measurements (*n* = 11,374). We found that Centaur’s representations consistently outperformed Llama’s representations in predicting human neural activity (all pairwise one-sided *t*-tests, *P* ≤ 0.001), indicating that fine-tuning a model on large-scale behavioural data aligned its internal representations to human neural activity. It is worth noting that this type of analysis was possible only because of the expressivity of Centaur’s representations, and that using representations of a conventional cognitive model led to a substantial drop in performance (dashed line in Fig. 4b). A more fine-grained report of our results is given in Extended Data Fig. 6.

We expanded these results in a second analysis, for which we relied on a previously collected dataset involving fMRI measurements of people reading simple six-word sentences, such as “That is such a beautiful picture!”^38^. The primary goal of this analysis was to show that neural alignment in unrelated settings remains intact after fine-tuning on cognitive experiments. We focused on a subset of five participants who each passively read 1,000 sentences, spread across 20 experimental runs and two scanning sessions. The presented sentences were extracted from nine corpora and selected to maximize semantic diversity. We closely followed the protocol of the original study and predicted aggregated neural activity across participants in the language network. We repeated this procedure for representations extracted from different layers in both Centaur and Llama. Predictability peaked at around layer 20, as shown in Fig. 4c. This peak is consistent with the hypothesis that the intermediate layers of such models contain the most information. We performed an inverse-weighted meta-analysis^39^ on the difference in correlations between Centaur and Llama, and this indicated that there was a significant benefit of fine-tuning when pooling across layers (*β* = 0.007, 95% confidence interval [0.0002, 0.013], *P* = 0.045). Although this effect was consistent across layers, it was not statistically significant for any individual layer.

## Model-guided scientific discovery

**Fig. 5:**
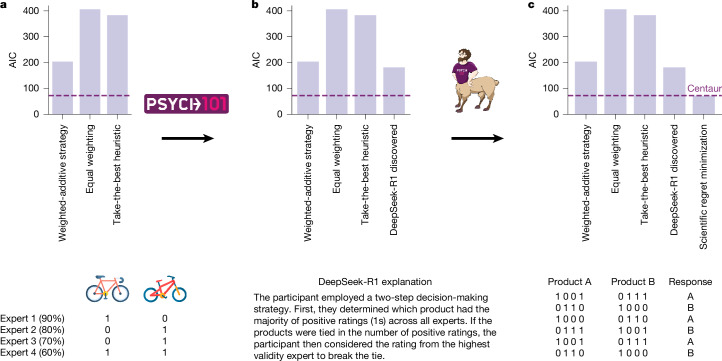
Model-guided scientific discovery. **a**, We used Psych-101 and Centaur to guide the development of a cognitive model for a multi-attribute decision-making study^41^. Each panel shows the AIC for the set of models considered at the given stage, starting with the models considered in the original study. **b**, We asked DeepSeek-R1 to generate an explanation for the human responses and formalized the resulting verbal strategy into a formal computational model. **c**, We refined this model through scientific regret minimization using Centaur as a reference model. Six data points are shown for which Centaur makes accurate predictions but the DeepSeek-R1-discovered model does not. We then used this information to design a domain-specific cognitive model that is as predictive as Centaur but is still interpretable. The bicycle images in **a** are reproduced from Flaticon.com.

Psych-101 and Centaur both constitute valuable tools for scientific discovery. In the following section, we present an example of how each of them can be used to improve our understanding of human decision-making. The individual steps of this process are illustrated in Fig. 5.

Psych-101 contains human behavioural data in a natural-language format, which means it can be readily processed and analysed by a language-based reasoning model such as DeepSeek-R1 (ref. ^40^). To demonstrate this use case, we asked DeepSeek-R1 to generate an explanation of participants’ behaviour in a multi-attribute decision-making experiment^41^. In this paradigm, participants are given two different options that are each characterized by various features (in our case, four expert ratings for two products) and they must then decide which of the two options they prefer (Fig. 5a). The model produced several explanations, one of which caught our attention: “The participant employed a two-step decision-making strategy. First, they determined which product had the majority of positive ratings (1 s) across all experts. If the products were tied in the number of positive ratings, the participant then considered the rating from the highest validity expert to break the tie.” This strategy combines two well-known heuristic decision-making strategies that, as far as we know, have not been considered in this combination before. We then took this verbal strategy, implemented it as a formal computational model and found that it explained human response behaviour more accurately than the three strategies considered in the original study (a weighted-additive strategy, equal weighting and take-the-best heuristic; Fig. 5b).

However, the DeepSeek-R1-discovered model Akaike information criterion (AIC; 181.7) still fell short of the goodness-of-fit of Centaur (AIC, 72.5), indicating that there is still room for improvement. We therefore used a method known as scientific regret minimization, which uses a black-box predictive model as a reference to identify responses that are in principle predictable but are not captured by a given model^42^. Typically, scientific regret minimization requires the collection of a large-scale experiment-specific dataset to train this predictive model. Centaur, however, can be used out-of-the-box and without the need to collect any domain-specific data, thereby circumventing this step and broadening the scope of scientific regret minimization considerably (indeed, the multi-attribute decision-making data set under consideration contained fewer than 100 participants, placing it far out of reach for conventional scientific regret minimization). When inspecting the responses that were well predicted by Centaur but not by the DeepSeek-R1-discovered model, we observed that they all involved problems in which participants chose the option with fewer positive ratings overall but which was rated positively by a higher-validity expert (see Fig. 5c for an illustration of these problems and Methods, ‘Model-guided scientific discovery’ for further details). This pattern indicates that the switch between the two heuristics is probably not as strict as initially suggested by the DeepSeek-R1-discovered strategy. To capture this, we replaced the either-or rule with a weighted average of both heuristics. We found that the model that resulted from this process matched Centaur in terms of its goodness-of-fit (AIC, 71.7) but was still interpretable. We entered the resulting AIC values of all the models in a group-level model-selection procedure^43^ and estimated the protected exceedance probability, which is defined as the probability that a particular model has a higher frequency within a group than all the other candidate models. The protected exceedance probability of the model that resulted from scientific regret minimization was *P* = 0.83. Notably, the result of this model comparison stands in contrast to the one that was conducted with the original set of models and indicates that people rely on a combination of heuristics when making decisions, as opposed to following a weighted-additive strategy^44^.

## Discussion

In this paper we have introduced Centaur, a foundation model of human cognition that was obtained by fine-tuning a state-of-the-art language model on Psych-101, which is a large-scale dataset of human behaviour. This approach allowed us to leverage the vast knowledge embedded in large language models and also align them with human behaviour^13^. Centaur successfully captured human behaviour and passed a wide range of out-of-distribution checks. It generalized not only to unseen participants, but also to different cover stories, structural variations and entirely new domains. In addition to analysing the model on a behavioural level, we also conducted a series of analyses on its internal representations, in which we found increased alignment with human neural activity.

We also conducted a case study demonstrating how both Psych-101 and Centaur can be used for guiding the development of predictive, yet interpretable, cognitive models. The individual steps of our procedure are generic, so it could serve as a blueprint for model-guided scientific discovery in other experimental paradigms in the future. Looking beyond this example, Centaur finds many more applications in the context of automated cognitive science^45,46^. It may, for instance, be used for in silico prototyping of experimental studies^47^. In this context, one could use the model to figure out which designs lead to the largest effect sizes, how to design a study to reduce the number of required participants or to estimate the power of an effect.

The present paper takes initial steps in leveraging Centaur to gain deeper insights into human cognition, and it also opens up exciting new avenues for future exploration. First, one could further probe Centaur’s internal representations to understand how it represents knowledge and processes information. The resulting insights could, in turn, be used to generate hypotheses about knowledge representation and information processing in humans that could be validated in future experimental studies. We believe that tools such as sparse auto-encoders^48^ and attention map visualization^49^ provide promising avenues towards accomplishing this goal, and we hope to explore them in future studies.

Furthermore, it might also be possible to train models with different architectures from scratch using the dataset that we created in the process of this paper. Doing so would enable us to investigate the neural architecture of human cognition at a scale that could not have been done before. We might, for example, ask questions such as whether human information processing is better described by attention-based architectures^50^ or by architectures with a vector-based memory, or how much we can improve by incorporating theories from the neuroscience literature^51^. We expect an eventual outcome of such an approach to contain both domain-specific and domain-general modules, thereby allowing us to investigate the interplay between the two.

As far as we know, Psych-101 is already the broadest and largest dataset of human behaviour available, and we view its development as an ongoing process and plan to develop it further. The focus in its current state is largely on learning and decision-making, but we intend to eventually include more domains, such as psycholinguistics, social psychology and economic games. Experiments with information about individual differences are another source of neglected data in the current iteration of Psych-101. Ideally, we want to include all types of relevant information about subjects (including age, personality traits or socioeconomic status) in the prompt, such that a model trained on these data can capture individual differences. Experiments from developmental psychology or computational psychiatry provide an ideal source for this purpose. Finally, although we have already included some cross-cultural and meta-studies^52–55^, the current iteration still has a strong bias towards a Western, educated, industrialized, rich and democratic (WEIRD) population^56^.

Eventually, we hope to provide any psychological data in a standardized format that facilitates benchmarking, thereby complementing existing efforts from the neuroscience community^57,58^. Although the natural-language format (together with quite a bit of reverse-engineering) used in this work allows us to express a vast range of experimental paradigms, it introduces a selection bias against experiments that cannot be expressed in natural language. The long-term objective should therefore be to move towards a multimodal data format^59^.

## Conclusion

When the idea of a unified model of cognition was first proposed, researchers expressed concern that established areas of cognitive science might react negatively to such a model. In particular, they feared that the approach might be seen as unfamiliar or incompatible with existing theories, just like an “intruder with improper pheromones”^60^. This could lead to an “attack of the killer bees”, in which researchers in more-conventional fields would fiercely critique or reject the new model to defend their established approaches. To mitigate these concerns, the concept of a cognitive decathlon was proposed: a rigorous evaluation framework in which competing models of cognition are tested across ten experiments and judged on their cumulative performance in them. In the current work, we applied Centaur to the equivalent of 16 such cognitive decathlons, in which it was tested against numerous established models and consistently won every competition. This outcome indicates that the data-driven discovery of domain-general models of cognition is a promising research direction. The next step for future research should be to translate this domain-general computational model into a unified theory of human cognition^2^.

## Methods

### Data collection

We constructed Psych-101 by transcribing data from 160 psychological experiments into natural language. Each prompt was designed to include the entire trial-by-trial history of a complete session from a single participant. The experiments included were selected using the following criteria: publicly available data on a trial-by-trial level; the possibility of transcription into text without a significant loss of information; and coverage of a broad spectrum of domains. The transcription of each experiment was done manually by the authors. Approval from the institutional review board was obtained by the individual studies as required. We designed our natural-language prompts using the following principles: instructions should follow the original study as closely as possible; simplifications were made where appropriate; and a maximum prompt length of roughly 32,768 tokens was used. Full information about all the experiments included is provided in the Supplementary Information, Example prompts.

### Fine-tuning procedure

Llama 3.1 70B was the base model for our fine-tuning procedure. We used a parameter-efficient fine-tuning technique known as QLoRA^16^, which adds so-called low-rank adapters to each layer of a four-bit quantized base model. The base model was kept fixed during fine-tuning and only the parameters of the low-rank adapters were adjusted. We added low-rank adapters of rank *r* = 8 to all linear layers of the self-attention mechanisms and the feedforward networks. Each low-rank adapter modifies the forward pass as follows:

$$\begin{array}{l}{\bf{Y}}={\bf{XW}}+{\alpha }{\bf{X}}{{\bf{L}}}_{1}{{\bf{L}}}_{2}\\ {\bf{W}}\in {{\bf{R}}}^{{h}\times {o}}\,;{{\bf{L}}}_{1}\in {{\bf{R}}}^{{h}\times {r}}\,;{{\bf{L}}}_{2}\in {{\bf{R}}}^{{r}\times {o}},\end{array}$$

where **XW** is the (quantized) linear transformation of the base model and **XL**_1_**L**_2_ is the low-rank adapter component, with **X** being the input to the layer with dimensionality *h* and **Y** being the output of the layer with dimensionality* o*. The hyperparameter *α* controls the trade-off between the two. **R** is the set of real numbers. Low-rank adapter computations were performed in half-precision floating-point format. For further details on this technique, please see the original work^16^.

We fine-tuned the model for one epoch on the entire dataset using a standard cross-entropy loss (we experimented with prolonged training but found that this led to overfitting). We only back-propagated the loss at human responses and masked out the loss for all other tokens. The effective batch size was set to 32, the learning rate to 0.00005 and the weight decay to 0.01. We used an 8-bit AdamW optimizer^61^ with a linearly increasing warm up over the first 100 gradient steps. The fine-tuning procedure was implemented using the unsloth library (https://unsloth.ai/).

**Extended Data Fig. 7:**
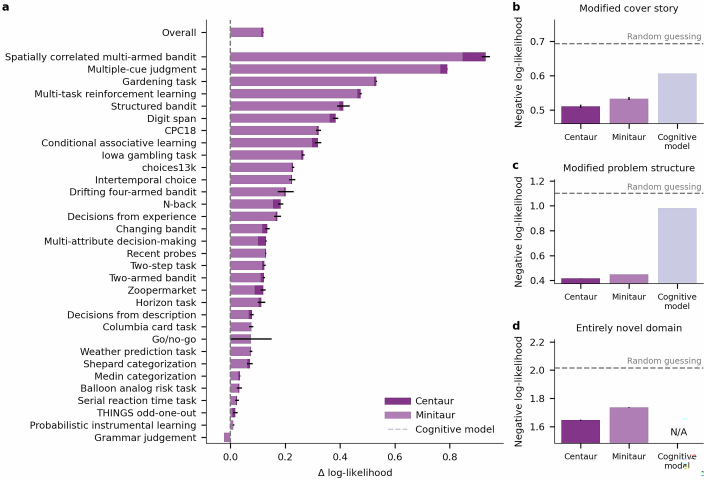
Log-likelihood comparison between Centaur and Minitaur on the analyses from the main text. **a**, Negative log-likelihoods relative to the domain-specific cognitive models on held-out participants from Psych-101. Error bars correspond to the standard error of the mean, taken over responses. **b**, Negative log-likelihoods for the two-step task with a modified cover story. **c**, Negative log-likelihoods for a three-armed bandit experiment. **d**, Negative log-likelihoods for an experiment probing logical reasoning with items based on the Law School Admission Test (LSAT).

We have also trained a smaller version of Centaur, called Minitaur, that uses Llama 3.1 8B as the base model following the same recipe. Minitaur captures human behaviour close to its training distribution but generalizes less robustly than the larger model to out-of-distribution experiments (Extended Data Fig. 7). Nevertheless, we believe that Minitaur is useful for prototyping because it does not require access to any specific hardware (it runs, for instance, on the free GPU instances in Google Colab).

### Evaluation metric

We used (negative) log-likelihoods averaged over responses as our evaluation metric. For experiments with multi-token responses, we summed log-likelihoods within a response and averaged across responses. We used one-sided *t*-tests whenever we tested whether Centaur outperformed a competing model in predicting human behaviour, because our hypotheses were directional and based on the prior expectation that Centaur would perform better. Because the number of observations in our analyses is generally large, reported significant effects survive after correcting for multiple comparisons where appropriate.

### Domain-specific cognitive models

We selected as our baseline models 14 cognitive and statistical models that together cover most of the experiments in Psych-101. Further details regarding the included models and their specifications are provided in Supplementary Information, Modelling details.

For our main analysis, we were interested in predicting the behaviour of held-out participants. Therefore, we fitted a joint set of parameters for all participants in the training data and evaluated how well a model with these parameters predicts the responses of held-out participants. Mirroring the evaluation metric for the language-based models, we evaluated goodness-of-fit using (negative) log-likelihoods averaged over responses.

For the out-of-distribution evaluations, we fitted model parameters using the most similar experiment in the training set, and then we evaluated how well a model with the resulting parameters predicts human responses in the unseen setting. The most similar experiment for the magic-carpet version of the two-step task was a two-step task experiment with the default spaceship cover story. The most similar experiment for Maggie’s farm was the horizon task. We included no baseline model for the logical reasoning task, because none of the experiments in the training data were similar to it.

### Neural alignment

The neural alignment analysis on the two-step task was conducted using data collected in a previous study^37^. We used a regularized linear regression model to predict fMRI data from internal representations of Centaur and Llama (a separate model was used for each participant and region). We fitted each of these models on data from two scanning blocks and evaluated them on data from the third. The regularization strength was selected using a nested cross-validation procedure. For each run, we split the beta maps into cortical and subcortical regions of interest (ROI) using the Schaefer 2018 atlas with 100 ROIs^62^. We averaged the betas within each ROI, reducing the number of betas from the number of voxels to the number of ROIs. All cortical and subcortical ROIs from the atlas were evaluated. Reported Pearson correlation coefficients correspond to the average across all ROIs.

Internal representations were extracted from the models’ residual stream and transformed using a principal component analysis. We set the number of retained components such that they explained 95% of the variance.

The fMRI data were preprocessed using fMRIPrep 24.0 (ref. ^63^). We used the default settings of fMRIPrep, and all the scans were aligned to the MNI152NLin2009cAsym atlas^64^. To extract effect estimates for each subtrial of the task (such as the second step of the fifth trial, or the feedback of the tenth trial), we built separate general linear models (GLMs). Each GLM included the subtrial of interest as a separate regressor, whose *z*-scored beta estimates were used for the alignment analysis. This part of the data was not modelled using other regressors. Furthermore, we included different regressors capturing all the first steps, all the second steps and all the feedback steps. Finally, we used six rotation and translation estimates as well as framewise displacement as noise regressors. The haemodynamic response was modelled using the spm^65^ model. A high-pass filter of 0.01 Hz and a Gaussian kernel with 6 mm full-width at half-maximum was applied. The GLMs were built using nilearn^66^.

The neural alignment analysis on the sentence-reading task was conducted using publicly available code from the original study^38^. No other changes were made apart from replacing GPT2-XL with Centaur and Llama. Please see the original study^38^ for further details.

### Model-guided scientific discovery

In our model-guided scientific discovery analysis, we focused on participants in the test set to avoid any potential contamination issues. We fitted parameters of all cognitive models individually for each participant using a maximum-likelihood estimation. Models were compared with each other using the AIC. The three models from the original study were implemented by the following equations:

$$\begin{array}{c}{\rm{p}}({\rm{a}}={\rm{A}}|{{\bf{x}}}_{{\rm{A}}},{{\bf{x}}}_{{\rm{B}}},{\rm{W}}{\rm{A}}{\rm{D}}{\rm{D}})\propto \exp (\beta \cdot {{\bf{w}}}_{{\rm{W}}{\rm{A}}{\rm{D}}{\rm{D}}}^{{\rm{\top }}}{{\bf{x}}}_{{\rm{A}}})\\ {{\bf{w}}}_{{\rm{W}}{\rm{A}}{\rm{D}}{\rm{D}}}=[0.9,\,0.8,\,0.7,\,0.6]\\ {\rm{p}}({\rm{a}}={\rm{A}}|{{\bf{x}}}_{{\rm{A}}},{{\bf{x}}}_{{\rm{B}}},{\rm{E}}{\rm{W}})\propto \exp (\beta \cdot {{\bf{w}}}_{{\rm{E}}{\rm{W}}}^{{\rm{\top }}}{{\bf{x}}}_{{\rm{A}}})\\ {{\bf{w}}}_{{\rm{E}}{\rm{W}}}=[1,\,1,\,1,\,1]\\ {\rm{p}}({\rm{a}}={\rm{A}}|{{\bf{x}}}_{{\rm{A}}},{{\bf{x}}}_{{\rm{B}}},{\rm{T}}{\rm{T}}{\rm{B}})\propto \exp (\beta \cdot {{\bf{w}}}_{{\rm{T}}{\rm{T}}{\rm{B}}}^{{\rm{\top }}}{{\bf{x}}}_{{\rm{A}}})\\ {{\bf{w}}}_{{\rm{T}}{\rm{T}}{\rm{B}}}=[1,\,0.5,\,0.25,\,0.125]\end{array},$$

where **x**_A_ and **x**_B_ are vectors containing four expert ratings (either 0 or 1) and *β* is a free parameter controlling the noise level.

We prompted DeepSeek-R1 (in the Distill-Llama-70B variant) to generate explanations of human decision-making; the corresponding prompt is provided in Supplementary Information, Model-guided scientific discovery. We then formalized the explanation shown in Fig. 5b into the following computational model:

$$p(a={\rm{A}},|{{\bf{x}}}_{{\rm{A}}},{{\bf{x}}}_{{\rm{B}}},{\rm{D}}{\rm{e}}{\rm{e}}{\rm{p}}{\rm{S}}{\rm{e}}{\rm{e}}{\rm{k}}-{\rm{R}}1)\propto \{\begin{array}{cc}\exp (\beta \cdot {{\bf{w}}}_{{\rm{T}}{\rm{T}}{\rm{B}}}^{{\rm{\top }}}{{\bf{x}}}_{{\rm{A}}}), & {\rm{i}}{\rm{f}}\sum _{i}{{\bf{x}}}_{{\rm{A}},{\rm{i}}}=\sum _{i}{{\bf{x}}}_{{\rm{B}},{\rm{i}}}\\ \exp (\beta \cdot {{\bf{w}}}_{{\rm{E}}{\rm{W}}}^{{\rm{\top }}}{{\bf{x}}}_{{\rm{A}}}), & {\rm{e}}{\rm{l}}{\rm{s}}{\rm{e}}\end{array}$$

For the scientific regret minimization pipeline, we computed the difference in log-likelihoods between Centaur and the DeepSeek-R1-discovered model. We visualized and inspected the ten data points with the greatest difference. This process resulted in the following computational model:

$${\rm{p}}({\rm{a}}={\rm{A}}|{{\bf{x}}}_{{\rm{A}}},{{\bf{x}}}_{{\rm{B}}},{\rm{S}}{\rm{R}}{\rm{M}})\propto \exp (\beta \cdot (\sigma \cdot {{\bf{w}}}_{{\rm{T}}{\rm{T}}{\rm{B}}}^{{\rm{\top }}}{{\bf{x}}}_{{\rm{A}}}+(1-\sigma )\cdot {{\bf{w}}}_{{\rm{E}}{\rm{W}}}^{{\rm{\top }}}{{\bf{x}}}_{{\rm{A}}}))$$

where *σ* is a free parameter constrained between 0 and 1 that controls the trade-off between the two strategies.

### Reporting summary

Further information on research design is available in the Nature Portfolio Reporting Summary linked to this article.

## Online content

Any methods, additional references, Nature Portfolio reporting summaries, source data, extended data, supplementary information, acknowledgements, peer review information; details of author contributions and competing interests; and statements of data and code availability are available at 10.1038/s41586-025-09215-4.

## Supplementary information

Supplementary Information Supplementary Methods, containing prompts used for the model-guided scientific discovery analysis and details of the domain-specific cognitive models, and Supplementary Notes, containing a discussion of Centaur in the light of the criteria for a unified computational model^2^, and prompts for each experimental paradigm in Psych-101.

Reporting Summary

## Figures and Tables

**Extended Data Table 1:**
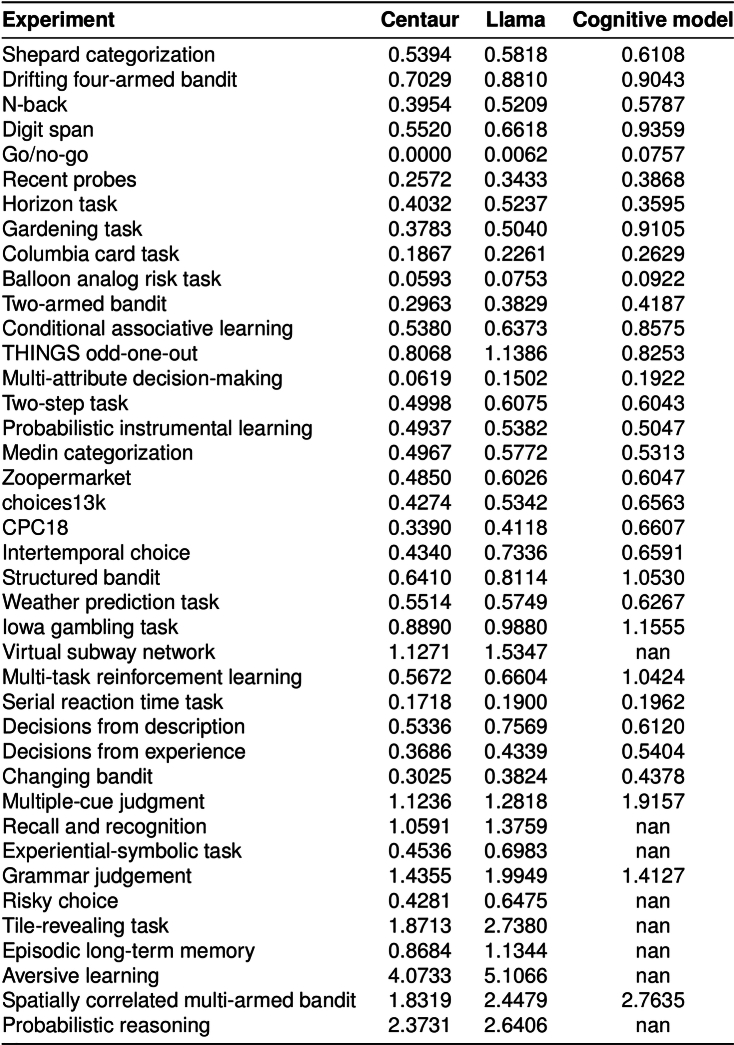
Psych-101 metrics

Full negative log-likelihoods on held-out participants.

## Data Availability

Psych-101 is publicly available on the Huggingface platform at https://huggingface.co/datasets/marcelbinz/Psych-101. The test set is accessible under a CC-BY-ND-4.0 licence through a gated repository at https://huggingface.co/datasets/marcelbinz/Psych-101-test.

## Code availability

Centaur is available on the Huggingface platform at https://huggingface.co/marcelbinz/Llama-3.1-Centaur-70B-adapter. The extra code needed to reproduce our results is available at https://github.com/marcelbinz/Llama-3.1-Centaur-70B.
