# Supplementary material for: A foundation model to predict and capture human cognition
Source: Nature. 2025 Jul 2;644(8078):1002–9. doi: 10.1038/s41586-025-09215-4 (PMC12390832; doi:10.1038/s41586-025-09215-4)
Supplement: Supplementary file 1 — Supplementary Methods, containing prompts used for the model-guided scientific discovery analysis and details of the domain-specific cognitive models, and Supplementary Notes, containing a discussion of Centaur in the light of the criteria for a unified computational model2, and prompts for each experimental paradigm in Psych-101. [file 41586_2025_9215_MOESM1_ESM.pdf]

nature portfolio

https://doi.org/10.1038/s41586-025-09215-4

Supplementary information

A foundation model to predict and capture human cognition

In the format provided by the authors and unedited

A foundation model of human cognition

Supplementary Information (SI)

Supplementary Methods 4
Model-guided scientific discovery 4
Modeling details 4
Generalized context model 4
Prospect theory model 5
Hyperbolic discounting model 5
Dual-systems model 5
Rescorla-Wagner model 6
Rescorla-Wagner model with context 6
Linear regression model 7
Weighted-additive model 7
Decision-updated reference point model 7
Odd-one-out model 8
Multi-task reinforcement learning model 8
GP-UCB model 8
Rational model 9
Lookup table model 9

Supplementary Notes 9
Newell test 9
Behave as an (almost) arbitrary function of the environment 9
Operate in real time 10
Exhibit rational, that is, effective adaptive behavior 10
Use vast amounts of knowledge about the environment 10
Behave robustly in the face of error, the unexpected, and the unknown 10
Integrate diverse knowledge 10
Use (natural) language 10
Exhibit self-awareness and a sense of self 11
Learn from its environment 11
Acquire capabilities through development 11
Arise through evolution 11
Be realizable within the brain 11
Example prompts 11
Shepard categorization 11

Drifting four-armed bandit . . . 13
Multiple-cue judgment . . . 15
Recall and recognition . . . 17
N-back . . . 20
Digit span . . . 23
Go/no-go . . . 27
Recent probes . . . 29
Horizon task . . . 31
Gardening task . . . 34
Columbia card task . . . 35
Balloon analog risk task . . . 37
Experiential-symbolic task . . . 40
Two-armed bandit . . . 41
Conditional associative learning . . . 44
THINGS odd-one-out . . . 46
Multi-attribute decision-making . . . 48
Grammar judgement . . . 49
Two-step task . . . 52
Two-step task . . . 53
Risky choice . . . 55
Tile-revealing task . . . 57
Probabilistic instrumental learning . . . 64
Medin categorization . . . 66
Zoopermarket . . . 68
choices13k . . . 70
CPC18 . . . 72
Episodic long-term memory . . . 74
Intertemporal choice . . . 75
Horizon task . . . 76
Structured bandit . . . 79
Horizon task . . . 81
Weather prediction task . . . 84
Iowa gambling task . . . 86
Virtual subway network . . . 88
Multi-task reinforcement learning . . . 89
Horizon task . . . 91
Horizon task . . . 94
Aversive learning . . . 96
Spatially correlated multi-armed bandit . . . 98
Serial reaction time task . . . 100
Decisions from description . . . 102
Decisions from experience . . . 102
Changing bandit . . . 105
Probabilistic reasoning . . . 107
Two-step task . . . 109
Evaluation data . . . 111

Two-step task (modified cover story) 111
Maggie's farm (modified problem structure) 113
Logical reasoning (entirely novel domain) 114
Two-step task (neural alignment) 116
Sentence reading (neural alignment) 118

Supplementary Methods

Model-guided scientific discovery

We generated an explanation for the behavior of every participant in the multi-attribute decision-making study of [1] using DeepSeek-R1 based on the following prompt template:

> In the following, you will see a transcript of a psychological experiment. Participant responses are highlighted within "<<" and ">>" characters. Your job will be to explain what strategies that participant used to solve the given task. Your explanation should be targeted at an expert cognitive scientist.
> ### START OF TRANSCRIPT ###
> [TRANSCRIPT]
> ### END OF TRANSCRIPT ###
> Please explain what strategies that participant used to solve the given task in less than two hundred words:

Modeling details

In the following, we list the domain-specific cognitive and statistical models used in our comparison. Each model was implemented in PyTorch [2]. We optimized a joint set of parameters for all participants in the training data by maximizing log-likelihoods of their choices and then evaluated how well a model with these parameters predicts choices of held-out participants. The optimization procedure involved 1000 iterations over the entire training set, and relied on a gradient-based algorithm [3] with an initial learning rate of 0.1. We use $\mathbb{1}$ to denote the indicator function that takes a value of one if the argument is true and zero otherwise.

Generalized context model

Reference: [4]
This model was used for the following experiments:

- Shepard categorization
- Medin categorization
- Weather prediction task

It uses the following log-likelihood:

$$p(c_t = i | x_t = \mathbf{x}_t) \propto \exp\left(\beta \sum_{k=1}^{t-1} \exp\left(-||\mathbf{x}_k - \mathbf{x}_t||_2\right) \cdot \mathbb{1}\left[y_k = i\right]\right)$$

where $\mathbf{x}_t$ are the features of the item observed at trial t and y_t is the corresponding class label. β is a free parameter of the model.

Prospect theory model

Reference: [5]
This model was used for the following experiments:

- CPC18
- choices13k
- Decisions from description

It uses the following log-likelihood:

$$
\begin{aligned}
p(c_t = i | p_i = \mathbf{p}_i, x_i = \mathbf{x}_i) &\propto \exp\left(\exp(\beta)\left(\pi(\mathbf{p}_i)^\top u(\mathbf{x}_i)\right)\right) \\
\pi(\mathbf{p}_i) &= \text{sigmoid}(a) + \text{sigmoid}(b) \cdot \mathbf{p}_i \\
u(\mathbf{x}_i) &= \begin{cases} \text{sigmoid}(c) \cdot \mathbf{x}_i^{\text{sigmoid}(d)} & \text{where } \mathbf{x}_i \geq 0 \\ -\text{sigmoid}(e) \cdot (-\text{sigmoid}(f)\, \mathbf{x}_i)^{\text{sigmoid}(g)} & \text{where } \mathbf{x}_i < 0 \end{cases}
\end{aligned}
$$

where $\mathbf{p}_i$ is the vector of probabilities and $\mathbf{x}_i$ is the vector of values for each possible outcome in option i. β, a, b, c, d, e, f, and g are free parameters of the model.

Hyperbolic discounting model

Reference: [6]
This model was used for the following experiments:

- Intertemporal choice

It uses the following log-likelihood:

$$
p(c_t = i | x_i = x_i, \gamma_i = \gamma_i) \propto \exp\left(\beta\left(x_i \cdot \frac{1}{1 + (a \cdot \gamma_i)}\right)\right)
$$

where x_i is the reward and γ_i is the delay of delivery for option i. β and a are free parameters of the model.

Dual-systems model

Reference: [7]
This model was used for the following experiments:

- Two-step task

It uses the following log-likelihood:

$$
p(c_t = i | s_t = s) \propto \begin{cases} \exp\left(\beta\left(\text{sigmoid}(\tau)\, Q^{\text{MB}}_{s,i} + (1 - \text{sigmoid}(\tau))\, Q^{\text{MF}}_{s,i}\right)\right) & \text{if } s = 0 \\ \exp\left(\beta Q^{\text{MF}}_{s,i}\right) & \text{if } s > 0 \end{cases}
$$

where $Q^{\text{MB}}_{s,i}$ and $Q^{\text{MF}}_{s,i}$ are model-based and model-free value estimates that are computed as described in [7]. β and τ are free parameters of the model. We also included a

stickiness term for the first stage choices, which is omitted for brevity in the equations above.

Rescorla-Wagner model

Reference: [8]
This model was used for the following experiments:

- Drifting four-armed bandit
- Horizon task
- Two-armed bandit
- Probabilistic instrumental learning
- Iowa gambling task
- Changing bandit
- Decisions from experience

It uses the following log-likelihood:

$$
\begin{aligned}
p(c_t = i) &\propto \exp\left(aV_{i,t} + bS_{i,t} + cI_{i,t}\right) \\
V_{i,t} &= \begin{cases} V_{i,t-1} + \text{sigmoid}\left(\alpha^{+}\right)\left(r_{t-1} - V_{i,t-1}\right) & \text{if } c_{t-1} = i \text{ and } r_{t-1} - V_{i,t-1} \geq 0 \\ V_{i,t-1} + \text{sigmoid}\left(\alpha^{-}\right)\left(r_{t-1} - V_{i,t-1}\right) & \text{if } c_{t-1} = i \text{ and } r_{t-1} - V_{i,t-1} < 0 \\ V_{i,t-1} & \text{otherwise} \end{cases} \\
S_{i,t} &= \mathbb{1}\left[c_{t-1} = i\right] \\
I_{i,t} &= \sum_{k=1}^{t-1} \mathbb{1}\left[c_k = i\right] \\
V_{i,1} &= d \\
S_{i,1} &= 0 \\
I_{i,1} &= 0
\end{aligned}
$$

where r_t is the reward obtained in trial t. α^+, α^-, a, b, c, and d are free parameters of the model.

Rescorla-Wagner model with context

Reference: [9]
This model was used for the following experiments:

- Conditional associative learning

It uses the following log-likelihood:

$$
\begin{aligned}
p(c_t = i | s_t = s) &\propto \exp\left(\beta V_{s,i,t}\right) \\
V_{s,i,t} &= \begin{cases} V_{s,i,t-1} + \text{sigmoid}\left(\alpha\right)\left(r_{t-1} - V_{s,i,t-1}\right) & \text{if } c_{t-1} = i \text{ and } s_{t-1} = s \\ V_{s,i,t-1} & \text{otherwise} \end{cases}
\end{aligned}
$$

$$V_{s,i,1} = d$$

where r_t is the reward obtained in trial t. α, β, and d are free parameters of the model.

Linear regression model

Reference: [10]
This model was used for the following experiments:

- Multiple-cue judgment
- Gardening task

It uses the following log-likelihood for multiple-cue judgment:

$$p(c_t = i | x_t = \mathbf{x}_t) \propto \exp\left(\beta \left(\mathbf{w}_t^\top \mathbf{x}_t - i\right)^2 + \gamma\right)$$

It uses the following log-likelihood for the gardening task:

$$p(c_t = \text{accept} | x_t = \mathbf{x}_t) \propto \exp\left(\beta \mathbf{w}_t^\top \mathbf{x}_t\right)$$
$$p(c_t = \text{reject} | x_t = \mathbf{x}_t) \propto \exp\left(0\right)$$

and the following learning rule for both tasks:

$$\mathbf{w}_t = \mathbf{w}_{t-1} + \alpha \left(r_{t-1} - \mathbf{w}_{t-1}^\top \mathbf{x}_{t-1}\right) \mathbf{x}_{t-1}$$
$$\mathbf{w}_1 = \mathbf{d}$$

where r_t is the reward obtained in trial t and $\mathbf{x}_t$ are the observed features. α, β, γ, and $\mathbf{d}$ are free parameters of the model.

Weighted-additive model

Reference: [11]
This model was used for the following experiments:

- Multi-attribute decision-making

It uses the following log-likelihood:

$$p(c_t = i | x_i = \mathbf{x}_i) \propto \exp\left(\mathbf{w}^\top \mathbf{x}_i\right)$$

where $\mathbf{x}_i$ is the vector of features for option i. $\mathbf{w}$ are free parameters of the model.

Decision-updated reference point model

Reference: [12]
This model was used for the following experiments:

- Columbia card task

It uses the following log-likelihood:

$$
\begin{aligned}
p(c_t = \text{sample}|x_{\text{win}}, x_{\text{loss}}, p_{\text{win}}, p_{\text{loss}}) &\propto \exp\left(h \cdot (x_{\text{win}} \cdot p_{\text{win}} + x_{\text{loss}} \cdot p_{\text{loss}}) + i\right) \\
p(c_t = \text{stop}|x_{\text{win}}, x_{\text{loss}}, p_{\text{win}}, p_{\text{loss}}) &\propto \exp\left(j\right) \\
\pi\left(p\right) &= \text{sigmoid}\left(a\right) + \text{sigmoid}\left(b\right) \cdot p \\
u\left(v\right) &= \begin{cases} \text{sigmoid}\left(c\right) \cdot v^{\text{sigmoid}(d)} & \text{where } v \geq 0 \\ -\text{sigmoid}\left(e\right) \cdot \left(-\text{sigmoid}\left(f\right) v\right)^{\text{sigmoid}(g)} & \text{where } v < 0 \end{cases}
\end{aligned}
$$

where x_{win} and x_{loss} are the values that can be won or lost respectively, and p_{win} and p_{loss} are the corresponding probabilities. a, b, c, d, e, f, g, h, i, and j are free parameters of the model.

Odd-one-out model

Reference: [13]
This model was used for the following experiments:

- THINGS odd-one-out

It uses the following log-likelihood:

$$p(c_t = i|x_i, x_j, x_k) \propto \exp\left(\mathbf{x}_j^\top \mathbf{x}_k\right)$$

where x_i, x_j, and x_k are the observed objects with their corresponding embeddings $\mathbf{x}_i$, $\mathbf{x}_j$, and $\mathbf{x}_k \in \mathbb{R}^{16}$ that are free parameters of the model.

Multi-task reinforcement learning model

Reference: [14]
This model was used for the following experiments:

- Multi-task reinforcement learning
- Zoopermarket

GP-UCB model

Reference: [15]
This model was used for the following experiments:

- Spatially correlated multi-armed bandit
- Structured bandit

It uses the following log-likelihood:

$$p(c_t = i) \propto \exp\left(\beta\left(\mathbf{m}_{i,t} + \exp\left(\gamma\right) \mathbf{s}_{i,t}\right)\right)$$

where $\mathbf{m}_{i,t}$ and $\mathbf{s}_{i,t}$ are obtained via Gaussian Process regression with a radial basis function kernel as described in [15]. β and γ are free parameters of the model.

Rational model

Reference: N/A
This model was used for the following experiments:

- Balloon analog risk task
- N-back
- Digit span
- Go/no-go
- Recent probes
- Serial reaction time task

It uses the following log-likelihood:

$$p(c_t = i | o_t = j) \propto \exp\left(\Theta_{j,i}\right)$$

where j is the optimal choice at trial t. $\Theta \in \mathbb{R}^{N_c \times N_c}$ are free parameters of the model.

Lookup table model

Reference: N/A
This model was used for the following experiments:

- Grammar judgement

It uses the following log-likelihood:

$$p(c_t = i) \propto \exp\left(\Theta_{t,i}\right)$$

where $\Theta \in \mathbb{R}^{T \times N_c}$ are free parameters of the model.

Supplementary Notes

Newell test

Together with his call for unified theories of cognition [16, 17], Newell outlined a set of criteria that a unified computational model should fulfill. Centaur is the first model to satisfy the majority of these criteria (see Table 1). Most importantly, it (1) behaves as an almost arbitrary function of the environment, (2) operates in real time, and (3) relies on vast amounts of knowledge about the world. We provide an extended discussion on Newell's criteria in the following.

Behave as an (almost) arbitrary function of the environment

This is the most important criterion according to Newell. Centaur fulfills it more than any previous model as shown by our extensive analysis. Yet, Centaur's scope is still limited to psychological experiments that can be expressed in natural language. It will be an important avenue for future research to transfer this ability to real-world applications.

Criterion	Fulfilled by Centaur
Behave as an (almost) arbitrary function of the environment	✓
Operate in real time	✓
Exhibit rational, that is, effective adaptive behavior	✓
Use vast amounts of knowledge about the environment	✓
Behave robustly in the face of error, the unexpected, and the unknown	✓
Integrate diverse knowledge	✓
Use (natural) language	✓
Exhibit self-awareness and a sense of self	•
Learn from its environment	✓
Acquire capabilities through development	✗
Arise through evolution	✗
Be realizable within the brain	✓

Supplementary Table 1 Newell test for a theory of cognition.

Operate in real time

Centaur can simulate human behavior in (almost) real-time. For example, running an open-loop simulation of a typical two-step task experiment takes around 30 minutes, while it takes around 20 minutes for the average human participant. We believe that inference time could be further optimized to fully close this gap.

Exhibit rational, that is, effective adaptive behavior

Bayesian inference is the gold standard for rational and adaptive behavior [18]. Previous work has shown that systems that engage in in-context learning implement Bayesian inference implicitly [19]. In-context learning is at the heart of Centaur, thereby making it a rational and adaptive system.

Use vast amounts of knowledge about the environment

Large language models are the biggest knowledge bases we have to date. As Centaur is built on top of a state-of-the-art language model, it fulfills this criterion by design.

Behave robustly in the face of error, the unexpected, and the unknown

Our extensive out-of-distribution evaluations clearly demonstrate that Centaur has this ability.

Integrate diverse knowledge

This was originally a criterion on symbols and abstractions [16]. At the basic level, Centaur is a system that processes language. Language is a symbolic system, meaning that Centaur fulfills this criterion.

Use (natural) language

No further elaboration is required.

Exhibit self-awareness and a sense of self

This is a tricky criterion as Centaur is trained on a population of individuals. Yet, to make good predictions about the future, Centaur needs to make inferences about the person who has produced a given trajectory, and in order to do that it requires a representation of that person [20].

Learn from its environment

Psych-101 contains many experiments that require learning from an environment. Centaur does well in those experiments, thereby clearly satisfying this criterion.

Acquire capabilities through development

Centaur makes no claims about how human behavior might arise through development.

Arise through evolution

Centaur makes no claims about how human behavior might arise through evolution.

Be realizable within the brain

We have shown that Centaur's internal representations are robust predictors of human neural activity. Even though there is clearly a gap between the transformer architecture that Centaur is based on and the human brain, Centaur still represents the current state-of-the-art when looking at neural alignment to human subjects.

Example prompts

The following sections present the experiments contained in Psych-101 in detail. Example prompts are truncated to 4096 characters but otherwise shown as is.

Shepard categorization

Data source: [21]

Number of experiments: 1
Number of participants: 85
Number of choices: 29776

Example prompt:
You will be shown several examples of geometric objects.
Your task is to learn a rule that allows you to tell whether an object belongs to the E or K category.
For each presented object, you will be asked to make a category judgment by pressing the corresponding key and then you will receive feedback.
You will encounter four different problems with different rules.

You encounter a new problem with a new rule determining which objects belong to

each category:
You see a big black square. You press <<K>>. The correct category is K.
You see a small black triangle. You press <<K>>. The correct category is E.
You see a big white triangle. You press <<E>>. The correct category is K.
You see a small white triangle. You press <<E>>. The correct category is E.
You see a small white square. You press <<E>>. The correct category is E.
You see a small black square. You press <<K>>. The correct category is E.
You see a big white square. You press <<E>>. The correct category is K.
You see a big black triangle. You press <<E>>. The correct category is K.
You see a big white square. You press <<E>>. The correct category is K.
You see a big black square. You press <<E>>. The correct category is K.
You see a small white triangle. You press <<K>>. The correct category is E.
You see a small black triangle. You press <<K>>. The correct category is E.
You see a big white triangle. You press <<E>>. The correct category is K.
You see a small white square. You press <<K>>. The correct category is E.
You see a small black square. You press <<K>>. The correct category is E.
You see a big black triangle. You press <<E>>. The correct category is K.
You see a small white square. You press <<E>>. The correct category is E.
You see a small black square. You press <<E>>. The correct category is E.
You see a big white triangle. You press <<K>>. The correct category is K.
You see a small black triangle. You press <<K>>. The correct category is E.
You see a small white triangle. You press <<E>>. The correct category is E.
You see a big white square. You press <<K>>. The correct category is K.
You see a big black triangle. You press <<E>>. The correct category is K.
You see a big black square. You press <<E>>. The correct category is K.
You see a small black triangle. You press <<E>>. The correct category is E.
You see a small white square. You press <<K>>. The correct category is E.
You see a small black square. You press <<E>>. The correct category is E.
You see a big black square. You press <<K>>. The correct category is K.
You see a big white square. You press <<K>>. The correct category is K.
You see a big white triangle. You press <<K>>. The correct category is K.
You see a small white triangle. You press <<E>>. The correct category is E.
You see a big black triangle. You press <<K>>. The correct category is K.
You see a small white square. You press <<E>>. The correct category is E.
You see a big black triangle. You press <<K>>. The correct category is K.
You see a big black triangle. You press <<K>>. The correct category is K.
You see a small white triangle. You press <<E>>. The correct category is E.
You see a big white triangle. You press <<K>>. The correct category is K.
You see a small white triangle. You press <<E>>. The correct category is E.
You see a small black triangle. You press <<E>>. The correct category is E.
You see a small black square. You press <<E>>. The correct category is E.
You see a small black triangle. You press <<E>>. The correct category is E.
You see a big white square. You press <<K>>. The correct category is K.
You see a big black square. You press <<K>>. The correct category is K.
You see a big white square. You press <<K>>. The correct category is K.

You see a big black square. You press <<K>>. The correct category is K.
You see a small black square. You press <<E>>. The correct category is E.
You see a big white triangle. You press <<E>>. The correct category is K.
You see a small white square. You press <<E>>. The correct category is E.
You see a big white square. You press <<K>>. The correct category is K.
You see

Drifting four-armed bandit

Data source: [22]

Number of experiments: 1
Number of participants: 869
Number of choices: 125952

Example prompt:
You will be asked to repeatedly choose between four different options labeled L, G, O, and U.
You select an option by pressing the corresponding key on your keyboard.
Each time you select an option, you will get a different number of points.
Your goal is to win as many points as possible.

You press <<L>> and get 84.0 points.
You press <<G>> and get 90.0 points.
You press <<O>> and get 53.0 points.
You press <<U>> and get 24.0 points.
You press <<G>> and get 92.0 points.
You press <<G>> and get 78.0 points.
You press <<L>> and get 71.0 points.
You press <<L>> and get 75.0 points.
You press <<G>> and get 80.0 points.
You press <<G>> and get 80.0 points.
You press <<G>> and get 91.0 points.
You press <<G>> and get 90.0 points.
You press <<U>> and get 29.0 points.
You press <<O>> and get 45.0 points.
You press <<G>> and get 81.0 points.
You press <<G>> and get 75.0 points.
You press <<G>> and get 82.0 points.
You press <<G>> and get 82.0 points.
You press <<G>> and get 87.0 points.
You press <<G>> and get 85.0 points.
You press <<G>> and get 87.0 points.
You press <<G>> and get 87.0 points.
You press <<G>> and get 79.0 points.
You press <<G>> and get 75.0 points.

You press <<L>> and get 61.0 points.
You press <<O>> and get 40.0 points.
You press <<U>> and get 37.0 points.
You press <<G>> and get 72.0 points.
You press <<G>> and get 73.0 points.
You press <<L>> and get 66.0 points.
You press <<G>> and get 57.0 points.
You press <<L>> and get 64.0 points.
You press <<L>> and get 63.0 points.
You press <<L>> and get 61.0 points.
You press <<O>> and get 54.0 points.
You press <<U>> and get 30.0 points.
You press <<L>> and get 59.0 points.
You press <<O>> and get 56.0 points.
You press <<O>> and get 46.0 points.
You press <<L>> and get 59.0 points.
You press <<G>> and get 63.0 points.
You press <<G>> and get 63.0 points.
You press <<G>> and get 58.0 points.
You press <<L>> and get 53.0 points.
You press <<O>> and get 60.0 points.
You press <<O>> and get 59.0 points.
You press <<L>> and get 52.0 points.
You press <<O>> and get 54.0 points.
You press <<U>> and get 23.0 points.
You press <<G>> and get 52.0 points.
You press <<O>> and get 52.0 points.
You press <<L>> and get 67.0 points.
You press <<L>> and get 71.0 points.
You press <<L>> and get 71.0 points.
You press <<L>> and get 69.0 points.
You press <<G>> and get 46.0 points.
You press <<O>> and get 47.0 points.
You press <<U>> and get 19.0 points.
You press <<L>> and get 63.0 points.
You press <<L>> and get 58.0 points.
You press <<L>> and get 62.0 points.
You press <<L>> and get 53.0 points.
You press <<L>> and get 59.0 points.
You press <<L>> and get 65.0 points.
You press <<G>> and get 58.0 points.
You press <<O>> and get 54.0 points.
You press <<L>> and get 61.0 points.
You press <<G>> and get 66.0 points.
You press <<O>> and get 62.0 points.

You press <<L>> and get 61.0 points.
You press <<G>> and get 56.0 points.
You press <<O>> and get 58.0 points.
You press <<L>> and get 49.0 points.
You press <<O>> and get 50.0 points.
You press <<G>> and get 63.0 points.
You press <<G>> and get 68.0 points.
You press <<G>> and get 56.0 points.
You press <<G>> and get 59.0 points.
You press <<G>> and get 66.0 points.
You press <<G>> and get 56.0 points.
You press <<G>> and get 56.0 points.
You press <<L>> and get 48.0 points.
You press <<G>> and get 59.0 points.
You press <<G>> and get 55.0 points.
You press <<O>> and get 51.0 points.
You press <<O>> and get 58.0 points.
You press <<O>> and get 51.0 points.
You press <<O>> and get 62.0 points.
You press <<O>> and get 64.0 points.
You press <<O>> and get 60.0 points.
You press <<O>> and get 62.0 points.
You press <<O>> and get 64.0 points.
You press <<O>> and get 50.0 points.
You press <<U>> and get 40.0 points.
You press <<O>> and get 50.0 points.
You press <<G>> and get 49.0 points.
You press <<L>> and get 33.0 points.
You press <<G>> and get 55.0 points.
You press <<O>> and get 78.0 points.
You press <<O>> and get 79.0 points.
You press <<O>> and get 85.0 points.
You press <<O>> and get 82.0 points.
You press <<O>> and get 83.0 po

Multiple-cue judgment

Data source: [23]

Number of experiments: 3
Number of participants: 232
Number of choices: 52464

Example prompt:
Your task is to estimate the blood concentration of the hormone Caldionine based on information about the amount of two other hormones, Progladine and Amalydine, in

multiple individuals' urine.
Both Progladine and Amalydine can take five values (very little, a little, average, a lot, very much).
Caldionine can take nine values (extremely low, very low, low, somewhat low, normal, somewhat high, high, very high, extremely high).
Your goal is to estimate the concentration of Caldionine correctly.
You will receive feedback about the actual concentration after making your estimate.
This feedback will stop at some point.

Progladine: a lot. Amalydine: very much. You say that the Caldionine concentration is <<high>>. That is incorrect. The correct concentration of Caldionine is somewhat low.
Progladine: average. Amalydine: a lot. You say that the Caldionine concentration is <<somewhat low>>. That is correct. The correct concentration of Caldionine is indeed somewhat low.
Progladine: a lot. Amalydine: average. You say that the Caldionine concentration is <<normal>>. That is incorrect. The correct concentration of Caldionine is somewhat high.
Progladine: average. Amalydine: a little. You say that the Caldionine concentration is <<low>>. That is incorrect. The correct concentration of Caldionine is somewhat high.
Progladine: a little. Amalydine: average. You say that the Caldionine concentration is <<normal>>. That is incorrect. The correct concentration of Caldionine is somewhat low.
Progladine: a lot. Amalydine: a little. You say that the Caldionine concentration is <<very high>>. That is incorrect. The correct concentration of Caldionine is high.
Progladine: very little. Amalydine: very little. You say that the Caldionine concentration is <<normal>>. That is correct. The correct concentration of Caldionine is indeed normal.
Progladine: very much. Amalydine: a little. You say that the Caldionine concentration is <<very high>>. That is correct. The correct concentration of Caldionine is indeed very high.
Progladine: a lot. Amalydine: very little. You say that the Caldionine concentration is <<very high>>. That is correct. The correct concentration of Caldionine is indeed very high.
Progladine: a little. Amalydine: a little. You say that the Caldionine concentration is <<normal>>. That is correct. The correct concentration of Caldionine is indeed normal.
Progladine: a little. Amalydine: very little. You say that the Caldionine concentration is <<somewhat high>>. That is correct. The correct concentration of Caldionine is indeed somewhat high.
Progladine: very little. Amalydine: a little. You say that the Caldionine concentration is <<somewhat low>>. That is correct. The correct concentration of Caldionine is indeed somewhat low.
Progladine: very much. Amalydine: a lot. You say that the Caldionine concentration

is <<very high>>. That is incorrect. The correct concentration of Caldionine is somewhat high.
Progladine: a little. Amalydine: very much. You say that the Caldionine concentration is <<very low>>. That is correct. The correct concentration of Caldionine is indeed very low.
Progladine: average. Amalydine: very much. You say that the Caldionine concentration is <<low>>. That is correct. The correct concentration of Caldionine is indeed low.
Progladine: very much. Amalydine: average. You say that the Caldionine concentration is <<somewhat high>>. That is incorrect. The correct concentration of Caldionine is high.
Progladine: average. Amalydine: very little. You say that the Caldionine concentration is <<high>>. That is correct. The correct concentration of Caldionine is indeed high.
Progladine: a little. Amalydine: a lot. You say that the Caldionine concentration is <<normal>>. That is incorrect. The correct concentration of Caldionine is low.
Progladine: very much. Amalydine: very much. You say that the Caldionine concentration is <<normal>>. That is correct. The correct concentration of Caldionine is indeed normal.
Progladine: a lot. Amalydine: a lot. You say that the Caldionine concentration is <<normal>>. That is correct.

Recall and recognition

Data source: [24]

Number of experiments: 1
Number of participants: 424
Number of choices: 109201

Example prompt:
You study the following 20 word pairs:
FILE, GERMAN
STANDS, RISES
OFFICER, FUEL
CLASSROOM, JOURNEY
TERRITORY, EDUCATIONAL
TRANSPORTATION, AGREEMENT
SIGNIFICANT, SPECIALIZED
TUBE, ENEMY
CLUB, RAPID
LEGAL, CONCEPT
TOWARDS, VICTORY
RARELY, BAY
ELECTION, ROYAL
BELONG, SUPREME

FRIGHTENED, PLAINS
SLAVES, FILM
STOMACH, WHISPERED
NURSE, GOLDEN
POLITICS, WINGS
BOXES, TOM

You will now view a single pair of words.
Your task is to indicate if the pair of words you see on the screen was studied as a pair on the list you just studied (by pressing D) or was not a pair (by pressing N).

You view the word pair SPECIALIZED, POLITICS. You press <<N>>.
You view the word pair TOWARDS, VICTORY. You press <<D>>.
You view the word pair SUPREME, BELONG. You press <<D>>.
You view the word pair WINGS, OFFICER. You press <<D>>.
You view the word pair CONCEPT, FRIGHTENED. You press <<N>>.
You view the word pair GERMAN, NURSE. You press <<N>>.
You view the word pair BAY, RARELY. You press <<D>>.
You view the word pair FILE, GOLDEN. You press <<N>>.
You view the word pair JOURNEY, LEGAL. You press <<N>>.
You view the word pair CLASSROOM, ENEMY. You press <<N>>.
You view the word pair ROYAL, ELECTION. You press <<D>>.
You view the word pair BOXES, TOM. You press <<D>>.
You view the word pair TERRITORY, EDUCATIONAL. You press <<D>>.
You view the word pair WHISPERED, STOMACH. You press <<D>>.
You view the word pair STANDS, RISES. You press <<D>>.
You view the word pair AGREEMENT, TUBE. You press <<N>>.
You view the word pair FUEL, TRANSPORTATION. You press <<N>>.
You view the word pair CLUB, RAPID. You press <<D>>.
You view the word pair SLAVES, FILM. You press <<D>>.
You view the word pair PLAINS, SIGNIFICANT. You press <<N>>.

You study the following 20 word pairs:
METALS, BEAUTY
SHORE, COLONISTS
HIGHEST, FASHION
INFLUENCED, FLEW
SHADOW, GRANDFATHER
MOTOR, DISAPPEARED
DETAIL, SHOULDERS
REPORTS, MALE
PAINT, COMMUNITIES
STORM, RESULTING
SELECTION, NIGHTS
APPLICATION, FELLOW

DESIGN, PRINCIPLE
POSSIBILITY, AFRICAN
REMOVE, TRAIL
STAFF, JUSTICE
SECTIONS, TRADITIONAL
CLAY, MIXED
TEA, AGRICULTURE
PATIENT, LIMIT

You will now view a single word item.
Your task is to type the other word in the pair.
For example if you studied BRICK, BRACK and you now see BRICK, your response should be BRACK.
The spelling does not matter; focus on providing as many responses as possible.
If you cannot recall the word, answer DON'T REMEMBER.

You view the word STAFF. You answer <<JUSTICE>>.
You view the word MOTOR. You answer <<DON'T REMEMBER>>.
You view the word INFLUENCED. You answer <<DON'T REMEMBER>>.
You view the word DETAIL. You answer <<SHOULDER>>.
You view the word REMOVE. You answer <<DON'T REMEMBER>>.
You view the word PATIENT. You answer <<LIMITED>>.
You view the word PAINT. You answer <<COMMUNITY>>.
You view the word STORM. You answer <<RESULTS>>.
You view the word TEA. You answer <<ARIGCULTURE>>.
You view the word SHADOW. You answer <<GRANDFATHER>>.
You view the word APPLICATION. You answer <<FELLOW>>.
You view the word METALS. You answer <<BEAUTY>>.
You view the word POSSIBILITY. You answer <<AFRICANS>>.
You view the word DESIGN. You answer <<PRINCPLE>>.
You view the word SHORE. You answer <<DON'T REMEMBER>>.
You view the word HIGHEST. You answer <<DON'T REMEMBER>>.
You view the word CLAY. You answer <<MIXED>>.
You view the word REPORTS. You answer <<DON'T REMEMBER>>.
You view the word SECTIONS. You answer <<DON'T REMEMBER>>.
You view the word SELECTION. You answer <<DON'T REMEMBER>>.

You study the following 20 word pairs:
DEFENSE, EXPANSION
PROGRESS, PROMISE
EGG, ATTRACTIVE
CRYING, RECOGNIZE
PURE, AUNT
OBTAINED, FEATURE
TRAVELED, FLOWER

INCREASINGLY, FEMALE
POURED, VALUABLE
TIGHT, HOLDS
BIGGER, HONOR
DRUG, SPENDING
DOUBLE, OUTPUT
ESTABLISH, CRITICAL
DRIED, CHRISTMAS
PROCESSING, NUMEROUS
MEASURES, PARAGRAPH
AFFAIRS, MOVEMENTS
DAMAGE, FED
WARS, CONTRACT

You will now view a single word item.
Your task is to indicate if the item you view was on the list you just studied (by pressing D) or not on the list (by pressing N).

You view the word EXPANSION. You press <<D>>.

N-back

Data source: [25]

Number of experiments: 1
Number of participants: 470
Number of choices: 200821

Example prompt:
You will view a stream of letters on the screen, one letter at a time.
At the beginning of a block, you are told a number N.
You have to remember the last N letters you saw since the beginning of the block.
If the letter you see matches the letter N trials ago, press W, otherwise press D.
The case of the letter is irrelevant, so ”t” matches ”T” for example.
If you make more than 5 mistakes in a block, N is decreased by 1.
If you make fewer than 3 mistakes in a block, N is increased by 1.
You will go through 20 blocks with 20+N trials each.

Block 0, N = 2:
You see the letter d.
You see the letter g.
You see the letter D and press <<W>>.
You see the letter v.
You see the letter B.
You see the letter V.
You see the letter d.

You see the letter g.
You see the letter D and press <<W>>.
You see the letter t.
You see the letter G.
You see the letter B and press <<D>>.
You see the letter G.
You see the letter g and press <<W>>.
You see the letter g and press <<W>>.
You see the letter v.
You see the letter V.
You see the letter B.
You see the letter G.
You see the letter G.
You see the letter G and press <<W>>.
You see the letter G.

Block 1, N = 1:
You see the letter g.
You see the letter D and press <<D>>.
You see the letter D.
You see the letter d and press <<W>>.
You see the letter D.
You see the letter d and press <<W>>.
You see the letter v.
You see the letter b and press <<D>>.
You see the letter V.
You see the letter t and press <<D>>.
You see the letter d.
You see the letter b and press <<D>>.
You see the letter G.
You see the letter t and press <<D>>.
You see the letter D.
You see the letter t and press <<D>>.
You see the letter t.
You see the letter b and press <<D>>.
You see the letter B.
You see the letter B and press <<W>>.
You see the letter v.

Block 2, N = 1:
You see the letter g.
You see the letter g and press <<W>>.
You see the letter d.
You see the letter d and press <<W>>.
You see the letter T.
You see the letter v.

You see the letter V.
You see the letter D and press <<W>>.
You see the letter g.
You see the letter G and press <<W>>.
You see the letter t.
You see the letter D and press <<D>>.
You see the letter t.
You see the letter g and press <<D>>.
You see the letter g.
You see the letter D and press <<W>>.
You see the letter d.
You see the letter V.
You see the letter B.
You see the letter v and press <<D>>.
You see the letter V.

Block 3, N = 1:
You see the letter T.
You see the letter d and press <<W>>.
You see the letter t.
You see the letter V and press <<D>>.
You see the letter v.
You see the letter B and press <<D>>.
You see the letter b.
You see the letter D and press <<D>>.
You see the letter d.
You see the letter d and press <<W>>.
You see the letter G.
You see the letter B and press <<D>>.
You see the letter G.
You see the letter d.
You see the letter G.
You see the letter v and press <<D>>.
You see the letter B.
You see the letter t and press <<D>>.
You see the letter T.
You see the letter T and press <<W>>.
You see the letter T.

Block 4, N = 1:
You see the letter d.
You see the letter d and press <<W>>.
You see the letter d.
You see the letter V and press <<D>>.
You see the letter V.

You see the letter v and press <<D>>.
You see the letter B.
You see the letter B and press <<W>>.
You see the letter g.
You see the letter B.
You see the letter t.
You see the letter D.
You see the letter d.
You see the letter B.
You see the letter B.
You see the letter t.
You see the letter d.
You see the letter G.
You see the letter d.
You see the letter v.
You see the letter t.

Block 5, N = 1:
You see the letter v.
You see the letter t and press <<D>>.
You see the letter D.
You see the letter V and press <<D>>.
You see the letter v.
You see the letter D and press <<D>>.
You see the letter D.
You see the letter B and press <<D>>.
You see the letter g.
You see the letter v and press <<D>>.
You see the letter d.
You see the letter g.
You see the letter G.
You see the letter D and press <<D>>.
You see the letter b.
You see the letter v.
You see the letter V.
You see the letter D and press <<D>>.
You see the

Digit span

Data source: [25]

Number of experiments: 1
Number of participants: 472
Number of choices: 97012

Example prompt:
You will view a series of digits and are then asked to recall them in the order you have seen them by pressing the corresponding keys.
After having recalled all digits, please press 'S' to indicate the end of your recalled sequence.

The digits are the following: [4, 8, 2]
You press <<4>>.
You press <<8>>.
You press <<2>>.
You press <<S>>.

You will view a series of digits and are then asked to recall them in the order you have seen them by pressing the corresponding keys.
After having recalled all digits, please press 'S' to indicate the end of your recalled sequence.

The digits are the following: [5, 2, 8, 5]
You press <<5>>.
You press <<2>>.
You press <<8>>.
You press <<5>>.
You press <<S>>.

You will view a series of digits and are then asked to recall them in the order you have seen them by pressing the corresponding keys.
After having recalled all digits, please press 'S' to indicate the end of your recalled sequence.

The digits are the following: [9, 2, 8, 2, 5]
You press <<9>>.
You press <<2>>.
You press <<8>>.
You press <<2>>.
You press <<5>>.
You press <<S>>.

You will view a series of digits and are then asked to recall them in the order you have seen them by pressing the corresponding keys.
After having recalled all digits, please press 'S' to indicate the end of your recalled sequence.

The digits are the following: [8, 1, 4, 8, 4, 9]
You press <<8>>.
You press <<1>>.

You press <<4>>.
You press <<8>>.
You press <<8>>.
You press <<1>>.
You press <<S>>.

You will view a series of digits and are then asked to recall them in the order you have seen them by pressing the corresponding keys.
After having recalled all digits, please press 'S' to indicate the end of your recalled sequence.

The digits are the following: [5, 1, 7, 1, 6, 3]
You press <<7>>.
You press <<1>>.
You press <<5>>.
You press <<1>>.
You press <<6>>.
You press <<3>>.
You press <<S>>.

You will view a series of digits and are then asked to recall them in the order you have seen them by pressing the corresponding keys.
After having recalled all digits, please press 'S' to indicate the end of your recalled sequence.

The digits are the following: [5, 9, 5, 9, 1]
You press <<5>>.
You press <<9>>.
You press <<5>>.
You press <<9>>.
You press <<1>>.
You press <<S>>.

You will view a series of digits and are then asked to recall them in the order you have seen them by pressing the corresponding keys.
After having recalled all digits, please press 'S' to indicate the end of your recalled sequence.

The digits are the following: [7, 2, 5, 8, 2, 6]
You press <<7>>.
You press <<7>>.
You press <<2>>.
You press <<8>>.
You press <<5>>.
You press <<2>>.

You press <<S>>.

You will view a series of digits and are then asked to recall them in the order you have seen them by pressing the corresponding keys.
After having recalled all digits, please press 'S' to indicate the end of your recalled sequence.

The digits are the following: [8, 5, 8, 3, 9, 4]
You press <<8>>.
You press <<5>>.
You press <<8>>.
You press <<3>>.
You press <<9>>.
You press <<4>>.
You press <<S>>.

You will view a series of digits and are then asked to recall them in the order you have seen them by pressing the corresponding keys.
After having recalled all digits, please press 'S' to indicate the end of your recalled sequence.

The digits are the following: [9, 6, 9, 5, 1, 7, 1]
You press <<9>>.
You press <<6>>.
You press <<9>>.
You press <<5>>.
You press <<7>>.
You press <<1>>.
You press <<7>>.
You press <<S>>.

You will view a series of digits and are then asked to recall them in the order you have seen them by pressing the corresponding keys.
After having recalled all digits, please press 'S' to indicate the end of your recalled sequence.

The digits are the following: [7, 2, 5, 9, 1, 8]
You press <<7>>.
You press <<2>>.
You press <<5>>.
You press <<9>>.
You press <<1>>.
You press <<8>>.
You press <<S>>.

You will view a series of digits and are then asked to recall them in the order you have seen them by pressing the corresponding keys.
After having recalled all digits, please

Go/no-go

Data source: [25]

Number of experiments: 1
Number of participants: 463
Number of choices: 150517

Example prompt:
In this task, you need to emit responses to certain stimuli and omit responses to others.
You will see one of two colours, colour1 or colour2, on the screen in each trial.
You need to press button X when you see colour1 and press nothing when you see colour2.
You need to respond as quickly as possible.
You will be doing 10 practice trials followed by 350 test trials.

You see colour1 and press nothing.
You see colour2 and press <<X>> in 753.0ms.
You see colour2 and press <<X>> in 381.0ms.
You see colour2 and press nothing.
You see colour1 and press <<X>> in 473.0ms.
You see colour1 and press <<X>> in 713.0ms.
You see colour2 and press nothing.
You see colour1 and press <<X>> in 364.0ms.
You see colour2 and press nothing.
You see colour1 and press <<X>> in 378.0ms.
You see colour1 and press <<X>> in 794.0ms.
You see colour1 and press <<X>> in 436.0ms.
You see colour1 and press <<X>> in 427.0ms.
You see colour1 and press <<X>> in 337.0ms.
You see colour1 and press <<X>> in 269.0ms.
You see colour1 and press <<X>> in 312.0ms.
You see colour1 and press <<X>> in 273.0ms.
You see colour2 and press nothing.
You see colour1 and press <<X>> in 288.0ms.
You see colour1 and press <<X>> in 276.0ms.
You see colour1 and press <<X>> in 314.0ms.
You see colour1 and press <<X>> in 309.0ms.
You see colour1 and press <<X>> in 320.0ms.
You see colour1 and press <<X>> in 342.0ms.
You see colour1 and press <<X>> in 301.0ms.

You see colour1 and press <<X>> in 289.0ms.
You see colour2 and press nothing.
You see colour1 and press <<X>> in 360.0ms.
You see colour2 and press <<X>> in 424.0ms.
You see colour2 and press nothing.
You see colour1 and press <<X>> in 525.0ms.
You see colour1 and press <<X>> in 306.0ms.
You see colour1 and press <<X>> in 387.0ms.
You see colour1 and press <<X>> in 292.0ms.
You see colour1 and press <<X>> in 317.0ms.
You see colour1 and press <<X>> in 270.0ms.
You see colour1 and press <<X>> in 278.0ms.
You see colour2 and press nothing.
You see colour1 and press <<X>> in 277.0ms.
You see colour1 and press <<X>> in 311.0ms.
You see colour1 and press <<X>> in 338.0ms.
You see colour1 and press <<X>> in 323.0ms.
You see colour1 and press <<X>> in 304.0ms.
You see colour1 and press <<X>> in 323.0ms.
You see colour1 and press <<X>> in 354.0ms.
You see colour1 and press <<X>> in 292.0ms.
You see colour1 and press <<X>> in 302.0ms.
You see colour1 and press <<X>> in 309.0ms.
You see colour2 and press nothing.
You see colour1 and press <<X>> in 340.0ms.
You see colour1 and press <<X>> in 603.0ms.
You see colour1 and press <<X>> in 289.0ms.
You see colour1 and press <<X>> in 284.0ms.
You see colour1 and press <<X>> in 275.0ms.
You see colour1 and press <<X>> in 299.0ms.
You see colour2 and press nothing.
You see colour1 and press <<X>> in 265.0ms.
You see colour1 and press <<X>> in 267.0ms.
You see colour2 and press nothing.
You see colour1 and press <<X>> in 274.0ms.
You see colour1 and press <<X>> in 382.0ms.
You see colour1 and press <<X>> in 272.0ms.
You see colour2 and press nothing.
You see colour1 and press <<X>> in 258.0ms.
You see colour1 and press <<X>> in 305.0ms.
You see colour1 and press <<X>> in 320.0ms.
You see colour1 and press <<X>> in 261.0ms.
You see colour1 and press <<X>> in 275.0ms.
You see colour2 and press nothing.
You see colour1 and press <<X>> in 424.0ms.

You see colour1 and press <<X>> in 266.0ms.
You see colour1 and press <<X>> in 273.0ms.
You see colour1 and press <<X>> in 287.0ms.
You see colour1 and press <<X>> in 437.0ms.
You see colour1 and press <<X>> in 293.0ms.
You see colour1 and press <<X>> in 297.0ms.
You see colour1 and press <<X>> in 308.0ms.
You see colour1 and press <<X>> in 313.0ms.
You see colour1 and press <<X>> in 373.0ms.
You see colour1 and press <<X>> in 390.0ms.
You see colour1 and press <<X>> in 304.0ms.
You see colour1 and press <<X>> in 334.0ms.
You see colour1 and press <<X>> in 326.0ms.
You see colour1 and press <<X>> in 382.0ms.
You see colour1 and press <<X>> in 803.0ms.
You see colour1 and press <<X>> in 430.0ms.
You see colour1 and press <<X>> in 324.0ms.
You see colour

Recent probes

Data source: [25]

Number of experiments: 1
Number of participants: 471
Number of choices: 34714

Example prompt:
You will repeatedly observe sequences of six letters.
You have to remember these letters before they disappear.
Afterward, you will be prompted with one letter. You have to answer whether the letter was part of the six previous letters.
If you think it was, you have to press C. If you think it was not, press Q.

You are shown the letters ['C', 'I', 'Q', 'F', 'W', 'Z']. You see the letter Y. You press <<Q>>.
You are shown the letters ['I', 'Q', 'C', 'D', 'M', 'V']. You see the letter U. You press <<Q>>.
You are shown the letters ['I', 'O', 'C', 'X', 'A', 'Q']. You see the letter M. You press <<C>>.
You are shown the letters ['Z', 'C', 'W', 'I', 'J', 'O']. You see the letter C. You press <<Q>>.
You are shown the letters ['Q', 'M', 'F', 'V', 'P', 'E']. You see the letter W. You press <<C>>.
You are shown the letters ['W', 'F', 'U', 'M', 'B', 'Q']. You see the letter V. You press <<Q>>.

You are shown the letters ['R', 'U', 'F', 'J', 'W', 'D']. You see the letter W. You press <<C>>.
You are shown the letters ['X', 'U', 'R', 'Y', 'H', 'F']. You see the letter X. You press <<Q>>.
You are shown the letters ['R', 'Q', 'M', 'X', 'V', 'U']. You see the letter W. You press <<C>>.
You are shown the letters ['G', 'Q', 'M', 'N', 'R', 'O']. You see the letter V. You press <<Q>>.
You are shown the letters ['T', 'P', 'Q', 'M', 'W', 'G']. You see the letter X. You press <<Q>>.
You are shown the letters ['P', 'J', 'Q', 'S', 'D', 'T']. You see the letter J. You press <<Q>>.
You are shown the letters ['J', 'R', 'H', 'Q', 'F', 'P']. You see the letter F. You press <<C>>.
You are shown the letters ['B', 'V', 'J', 'G', 'R', 'H']. You see the letter R. You press <<Q>>.
You are shown the letters ['X', 'J', 'V', 'L', 'B', 'D']. You see the letter B. You press <<Q>>.
You are shown the letters ['N', 'M', 'J', 'C', 'V', 'X']. You see the letter C. You press <<Q>>.
You are shown the letters ['N', 'T', 'J', 'R', 'M', 'W']. You see the letter C. You press <<Q>>.
You are shown the letters ['N', 'T', 'J', 'E', 'I', 'D']. You see the letter N. You press <<Q>>.
You are shown the letters ['J', 'T', 'N', 'K', 'C', 'B']. You see the letter T. You press <<C>>.
You are shown the letters ['M', 'O', 'N', 'T', 'P', 'J']. You see the letter O. You press <<Q>>.
You are shown the letters ['O', 'Q', 'W', 'U', 'M', 'N']. You see the letter Q. You press <<C>>.
You are shown the letters ['Q', 'Z', 'Y', 'O', 'I', 'W']. You see the letter I. You press <<C>>.
You are shown the letters ['Z', 'A', 'Y', 'F', 'Q', 'G']. You see the letter Q. You press <<C>>.
You are shown the letters ['Z', 'M', 'Y', 'P', 'A', 'B']. You see the letter X. You press <<Q>>.
You are shown the letters ['L', 'X', 'M', 'Z', 'Y', 'N']. You see the letter B. You press <<Q>>.
You are shown the letters ['M', 'J', 'X', 'C', 'L', 'U']. You see the letter P. You press <<Q>>.
You are shown the letters ['S', 'J', 'E', 'H', 'X', 'M']. You see the letter M. You press <<C>>.
You are shown the letters ['F', 'I', 'E', 'A', 'J', 'S']. You see the letter X. You press <<Q>>.
You are shown the letters ['D', 'U', 'I', 'M', 'O', 'T']. You see the letter Q. You press

<<Q>>.
You are shown the letters [’I’, ’D’, ’U’, ’G’, ’Q’, ’W’]. You see the letter B. You press <<Q>>.
You are shown the letters [’U’, ’I’, ’B’, ’L’, ’D’, ’Z’]. You see the letter W. You press <<Q>>.
You are shown the letters [’U’, ’E’, ’B’, ’I’, ’H’, ’R’]. You see the letter I. You press <<C>>.
You are shown the letters [’S’, ’B’, ’U’, ’E’, ’F’, ’N’]. You see the letter I. You press <<Q>>.
You are shown the letters [’Y’, ’S’, ’Q’, ’U’, ’B’, ’A’]. You see the letter Y. You press <<C>>.
You are shown the letters [’Q’, ’V’, ’Y’, ’S’, ’H’, ’O’]. You see the letter H. You press <<C>>.
You are shown the letters [’V’, ’Z’, ’P’, ’D’, ’Q’, ’Y’]. You see the letter H. You press <<C>>.
You are shown the letters [’Z’, ’P’, ’V’, ’W’, ’L’, ’N’]. You see the letter H. You press <<Q>>.
You are shown the letters [’U’, ’S’, ’Z’, ’V’, ’P’, ’M’]. You see the letter W. You press <<Q>>.
You are shown the letters [’U’, ’Y’, ’Z’, ’S’, ’C’, ’G’]. You see the letter W. You press <<Q>>.

Horizon task

Data source: [26]

Number of experiments: 1
Number of participants: 26
Number of choices: 29120

Example prompt:
You are participating in multiple games involving two slot machines, labeled I and H.
The two slot machines are different across different games.
Each time you choose a slot machine, you get some points.
You choose a slot machine by pressing the corresponding key.
Each slot machine tends to pay out about the same amount of points on average.
Your goal is to choose the slot machines that will give you the most points across the experiment.
The first 4 trials in each game are instructed trials where you will be told which slot machine to choose.
After these instructed trials, you will have the freedom to choose for either 1 or 6 trials.

Game 1. There are 10 trials in this game.
You are instructed to press I and get 73 points.
You are instructed to press H and get 91 points.

You are instructed to press I and get 68 points.
You are instructed to press H and get 95 points.
You press <<I>> and get 71 points.
You press <<H>> and get 96 points.
You press <<I>> and get 57 points.
You press <<I>> and get 30 points.
You press <<I>> and get 54 points.
You press <<H>> and get 81 points.

Game 2. There are 10 trials in this game.
You are instructed to press I and get 38 points.
You are instructed to press I and get 1 points.
You are instructed to press I and get 18 points.
You are instructed to press H and get 44 points.
You press <<I>> and get 5 points.
You press <<I>> and get 3 points.
You press <<H>> and get 53 points.
You press <<I>> and get 6 points.
You press <<H>> and get 37 points.
You press <<H>> and get 50 points.

Game 3. There are 5 trials in this game.
You are instructed to press H and get 24 points.
You are instructed to press H and get 34 points.
You are instructed to press I and get 68 points.
You are instructed to press I and get 53 points.
You press <<I>> and get 57 points.

Game 4. There are 10 trials in this game.
You are instructed to press H and get 53 points.
You are instructed to press I and get 57 points.
You are instructed to press H and get 45 points.
You are instructed to press H and get 51 points.
You press <<H>> and get 37 points.
You press <<I>> and get 35 points.
You press <<I>> and get 51 points.
You press <<I>> and get 37 points.
You press <<I>> and get 29 points.
You press <<H>> and get 53 points.

Game 5. There are 5 trials in this game.
You are instructed to press H and get 46 points.
You are instructed to press I and get 9 points.
You are instructed to press H and get 38 points.
You are instructed to press H and get 36 points.

You press <<H>> and get 37 points.

Game 6. There are 5 trials in this game.
You are instructed to press I and get 35 points.
You are instructed to press I and get 36 points.
You are instructed to press I and get 27 points.
You are instructed to press H and get 49 points.
You press <<H>> and get 41 points.

Game 7. There are 5 trials in this game.
You are instructed to press H and get 53 points.
You are instructed to press H and get 59 points.
You are instructed to press I and get 80 points.
You are instructed to press I and get 99 points.
You press <<H>> and get 54 points.

Game 8. There are 10 trials in this game.
You are instructed to press H and get 21 points.
You are instructed to press I and get 43 points.
You are instructed to press H and get 18 points.
You are instructed to press H and get 19 points.
You press <<H>> and get 26 points.
You press <<H>> and get 21 points.
You press <<I>> and get 47 points.
You press <<I>> and get 37 points.
You press <<I>> and get 37 points.
You press <<I>> and get 36 points.

Game 9. There are 5 trials in this game.
You are instructed to press H and get 47 points.
You are instructed to press H and get 41 points.
You are instructed to press I and get 40 points.
You are instructed to press I and get 35 points.
You press <<I>> and get 30 points.

Game 10. There are 5 trials in this game.
You are instructed to press I and get 69 points.
You are instructed to press I and get 69 points.
You are instructed to press H and get 65 points.
You are instructed to press I and get 68 points.
You press <<I>> and get 63 points.

Game 11. There are 10

Gardening task

Data source: [27]

Number of experiments: 1
Number of participants: 320
Number of choices: 192000

Example prompt:
You are going to plant trees in two different gardens labeled North and South.
The trees look different from each other regarding their leafiness and branchiness.
There are 5 levels of leafiness (0, 1, 2, 3, 4) and 5 levels of branchiness (0, 1, 2, 3, 4).
In each round, you get presented with a tree.
You can accept to plant the tree by pressing T and reject to plant it by pressing N.
If you accept to plant the tree and your answer is correct, you will be rewarded with points, otherwise, you will lose some points.
If you reject to plant the tree, you will not be rewarded (0 points).
Your task is to learn which type of tree grows best in each garden.
During the training phase, there will be feedback on every trial about your decisions.
During the testing phase, there will be no feedback for your decision.

You get a tree with level 3 of leafiness and level 0 of branchiness in the South garden. You press <<T>> and get -50 points. You would have gotten 0 points, had you rejected to plant the tree.
You get a tree with level 4 of leafiness and level 0 of branchiness in the North garden. You press <<T>> and get -50 points. You would have gotten 0 points, had you rejected to plant the tree.
You get a tree with level 1 of leafiness and level 1 of branchiness in the South garden. You press <<T>> and get -25 points. You would have gotten 0 points, had you rejected to plant the tree.
You get a tree with level 3 of leafiness and level 1 of branchiness in the North garden. You press <<T>> and get -25 points. You would have gotten 0 points, had you rejected to plant the tree.
You get a tree with level 0 of leafiness and level 4 of branchiness in the North garden. You press <<T>> and get 50 points. You would have gotten 0 points, had you rejected to plant the tree.
You get a tree with level 2 of leafiness and level 2 of branchiness in the North garden. You press <<N>> and get 0 points. You would have gotten 0 points, had you accepted to plant the tree.
You get a tree with level 0 of leafiness and level 2 of branchiness in the South garden. You press <<T>> and get 0 points. You would have gotten 0 points, had you rejected to plant the tree.
You get a tree with level 4 of leafiness and level 3 of branchiness in the North garden. You press <<N>> and get 0 points. You would have gotten -50 points, had you accepted to plant the tree.
You get a tree with level 1 of leafiness and level 1 of branchiness in the North garden.

You press <<T>> and get 25 points. You would have gotten 0 points, had you rejected to plant the tree.
You get a tree with level 2 of leafiness and level 1 of branchiness in the South garden. You press <<N>> and get 0 points. You would have gotten -25 points, had you accepted to plant the tree.
You get a tree with level 4 of leafiness and level 4 of branchiness in the North garden. You press <<N>> and get 0 points. You would have gotten -50 points, had you accepted to plant the tree.
You get a tree with level 1 of leafiness and level 3 of branchiness in the South garden. You press <<N>> and get 0 points. You would have gotten 25 points, had you accepted to plant the tree.
You get a tree with level 0 of leafiness and level 2 of branchiness in the South garden. You press <<N>> and get 0 points. You would have gotten 0 points, had you accepted to plant the tree.
You get a tree with level 4 of leafiness and level 1 of branchiness in the South garden. You press <<T>> and get -25 points. You would have gotten 0 points, had you rejected to plant the tree.
You get a tree with level 4 of leafiness and level 0 of branchiness in the North garden. You press <<N>> and get 0 points. You would have gotten -50 points, had you accepted to plant the tree.
You get a tree with level 1 of leafiness and level 1 of branchiness in the South garden. You press <<T>> and get -25 points. You would have gotten 0 points, had you rejected to plant the tree.
You get a tree with level 0 of leafiness and level 2 of branchiness in the South garden. You press <<N>> and get 0 points. You would have gotten 0 points, had you accepted to plant the tree.
You get a

Columbia card task

Data source: [28]

Number of experiments: 1
Number of participants: 1368
Number of choices: 613299

Example prompt:
You will play a games with 84 rounds.
In each round, you will be presented with 32 face-down cards.
Every card is either a gain card or a loss card.
If you turn over a gain card, the gain amount of that card (between 10 and 600 points) will be added to your current game score.
If you turn over a loss card, the loss amount of that card (between 25 and 750 points) will be subtracted from your game score.
In different rounds, between 1 and 28 cards are loss cards.
Loss and gain amounts also differ between rounds.

You may keep turning over cards as long as you keep encountering gain cards.
You may also stop the round at any point and claim your current payout.
If you encounter a loss card, the round ends immediately.
Your gains and losses will be summed up to give you your final score for each round.
Press E to turn a card over, or C to stop the round and claim your current payout.

Round 1:
You will be awarded 150 points for turning over a gain card.
You will lose 75 points for turning over a loss card.
There are 20 loss cards in this round.
You press <<E>> and turn over a loss card. Your current score is -75. The round has now ended because you encountered a loss card.
Your final score for this round is -75.

Round 2:
You will be awarded 50 points for turning over a gain card.
You will lose 100 points for turning over a loss card.
There are 1 loss cards in this round.
You press <<E>> and turn over a gain card. Your current score is 50.
You press <<E>> and turn over a gain card. Your current score is 100.
You press <<E>> and turn over a gain card. Your current score is 150.
You press <<E>> and turn over a gain card. Your current score is 200.
You press <<E>> and turn over a loss card. Your current score is 100. The round has now ended because you encountered a loss card.
Your final score for this round is 100.

Round 3:
You will be awarded 200 points for turning over a gain card.
You will lose 100 points for turning over a loss card.
There are 10 loss cards in this round.
You press <<E>> and turn over a gain card. Your current score is 200.
You press <<E>> and turn over a gain card. Your current score is 400.
You press <<E>> and turn over a gain card. Your current score is 600.
You press <<C>> and claim your payout.
Your final score for this round is 600.

Round 4:
You will be awarded 200 points for turning over a gain card.
You will lose 50 points for turning over a loss card.
There are 28 loss cards in this round.
You press <<C>> and claim your payout.
Your final score for this round is 0.

Round 5:
You will be awarded 20 points for turning over a gain card.

You will lose 750 points for turning over a loss card.
There are 1 loss cards in this round.
You press <<E>> and turn over a gain card. Your current score is 20.
You press <<E>> and turn over a gain card. Your current score is 40.
You press <<E>> and turn over a gain card. Your current score is 60.
You press <<E>> and turn over a gain card. Your current score is 80.
You press <<E>> and turn over a gain card. Your current score is 100.
You press <<C>> and claim your payout.
Your final score for this round is 100.

Round 6:
You will be awarded 300 points for turning over a gain card.
You will lose 100 points for turning over a loss card.
There are 16 loss cards in this round.
You press <<E>> and turn over a loss card. Your current score is -100. The round has now ended because you encountered a loss card.
Your final score for this round is -100.

Round 7:
You will be awarded 10 points for turning over a gain card.
You will lose 500 points for turning over a loss card.
There are 3 loss cards in this round.
You press <<E>> and turn over a gain card. Your current score is 10.
You press <<E>> and turn over a gain card. Your current score is 20.
You press <<E>> and turn over a gain card. Your current score is 30.
You press <<E>> and turn over a gain card. Your current score is 40.
You press <<C>> and claim your payout.
Your final score for this round is 40.

Round 8:
You will be awarded 10 points for turning over a gain card.
You will lose 250 points for turning over a loss

Balloon analog risk task

Data source: [28]

Number of experiments: 1
Number of participants: 1331
Number of choices: 1496974

Example prompt:
Throughout the task, you will be presented with balloons, one at a time.
In each step, you can choose to pump up the balloon by pressing H and you will accumulate 1 point for each pump.
At any point, you can stop pumping up the balloon by pressing W and you will

collect your accumulated points.
You will repeat this procedure on multiple different balloons.
It is your choice to determine how much to pump up the balloon, but be aware that at some point the balloon will explode.
If the balloon explodes before you collect your accumulated points, then you move on to the next balloon and the points are lost.

Balloon 1:
You press <<H>> <<H>> <<H>> <<H>> <<H>> <<H>> <<H>> <<H>> <<H>> <<H>> <<H>> <<H>> <<H>>. The balloon was inflated too much and explodes.

Balloon 2:
You press <<H>> <<H>> <<H>> <<H>> <<H>> <<H>> <<H>> <<H>> <<H>> <<H>> <<H>> <<H>> <<H>> <<H>> <<H>> <<H>> <<H>> <<H>> <<H>> <<H>> <<H>> <<H>> <<H>> <<H>> <<H>> <<H>> <<H>> <<H>> <<H>> <<H>> <<H>> <<H>> <<H>> <<H>> <<H>> <<H>> <<H>> <<H>> <<H>> <<H>> <<H>> <<H>> <<H>> <<H>> <<H>> <<H>> <<H>> <<H>> <<H>> <<H>> <<H>> <<H>> <<H>> <<H>> <<H>> <<H>> <<H>> <<H>> <<H>> <<H>> <<W>>. You stop inflating the balloon and get 60 points.

Balloon 3:
You press <<H>> <<H>> <<H>> <<H>> <<H>> <<H>> <<H>> <<H>> <<H>> <<H>> <<H>> <<H>> <<H>> <<H>> <<H>> <<H>> <<H>> <<H>> <<H>> <<H>> <<H>> <<H>>. The balloon was inflated too much and explodes.

Balloon 4:
You press <<H>> <<H>> <<H>> <<H>> <<H>> <<H>> <<H>> <<H>> <<H>> <<H>> <<H>> <<H>> <<H>> <<H>> <<H>> <<H>> <<H>> <<H>> <<H>> <<H>> <<H>> <<H>> <<H>> <<H>> <<H>> <<H>> <<H>> <<H>> <<H>> <<H>>. The balloon was inflated too much and explodes.

Balloon 5:
You press <<H>> <<H>> <<H>> <<H>> <<H>> <<H>> <<H>> <<H>> <<H>> <<H>> <<H>> <<H>> <<H>> <<H>> <<H>> <<H>> <<H>> <<H>> <<H>> <<H>> <<H>> <<H>> <<H>> <<H>> <<H>> <<H>> <<H>> <<H>> <<H>> <<H>> <<H>> <<H>> <<H>> <<H>> <<H>> <<H>> <<H>>. The balloon was inflated too much and explodes.

Balloon 6:
You press <<H>> <<H>> <<H>> <<H>> <<H>> <<H>> <<H>> <<H>> <<H>> <<H>> <<H>> <<H>> <<H>> <<H>> <<H>> <<H>> <<H>>

<<H>> <<W>>. You stop inflating the balloon and get 18 points.

Balloon 7:
You press <<H>> <<H>> <<H>> <<H>> <<H>> <<H>> <<H>> <<H>> <<H>> <<H>> <<H>> <<H>> <<H>> <<H>> <<H>> <<H>> <<H>> <<H>> <<H>> <<H>> <<H>> <<H>> <<H>> <<H>> <<H>> <<H>> <<H>> <<H>>. The balloon was inflated too much and explodes.

Balloon 8:
You press <<H>> <<H>> <<H>> <<H>> <<H>> <<H>> <<H>> <<H>> <<H>> <<H>> <<H>> <<H>> <<H>> <<H>> <<H>> <<H>> <<H>> <<H>> <<H>> <<H>> <<H>> <<H>> <<H>> <<W>>. You stop inflating the balloon and get 23 points.

Balloon 9:
You press <<H>> <<H>> <<H>> <<H>> <<H>> <<H>> <<H>> <<H>> <<H>> <<H>> <<H>> <<H>> <<H>> <<H>> <<H>> <<H>> <<H>> <<H>> <<H>> <<H>> <<H>> <<H>> <<H>> <<H>> <<H>> <<H>> <<W>>. You stop inflating the balloon and get 26 points.

Balloon 10:
You press <<H>> <<H>> <<H>> <<H>> <<H>> <<H>> <<H>> <<H>> <<H>> <<H>> <<H>> <<H>> <<H>> <<H>> <<H>> <<H>> <<H>> <<H>> <<H>> <<H>> <<H>> <<H>> <<H>> <<H>> <<H>> <<H>> <<H>> <<H>> <<H>> <<H>> <<H>> <<H>> <<H>> <<H>> <<H>> <<W>>. You stop inflating the balloon and get 35 points.

Balloon 11:
You press <<H>> <<H>> <<H>> <<H>> <<H>> <<H>> <<H>> <<H>> <<H>> <<H>> <<H>> <<H>> <<H>> <<H>> <<H>> <<H>> <<H>> <<H>> <<H>> <<H>> <<H>> <<H>> <<H>> <<H>> <<H>> <<H>> <<H>> <<H>> <<H>> <<H>> <<H>> <<H>> <<H>> <<H>> <<H>> <<W>>. You stop inflating the balloon and get 35 points.

Balloon 12:
You press <<H>> <<H>> <<H>> <<H>> <<H>> <<H>> <<H>> <<H>> <<H>> <<H>> <<H>> <<H>> <<H>> <<H>> <<H>> <<H>> <<H>> <<H>> <<H>> <<H>> <<H>> <<H>> <<H>> <<H>> <<H>> <<H>> <<H>> <<H>> <<H>> <<H>> <<H>> <<H>> <<H>> <<H>> <<H>> <<H>> <<H>> <<H>> <<H>> <<H>> <<H>> <<H>> <<H>> <<H>> <<H>> <<H>> <<H>> <<H>> <<H>> <<H>> <<W>>. You stop inflating the balloon and get 50 points.

Balloon 13:
You press <<H>> <<H>> <<H>> <<H>> <<H>> <<H>> <<H>> <<H>> <<H>> <<H>> <<H>> <<H>> <<H>> <<H>> <<H>> <<H>> <<H>>

<<H>> <<H>> <<H>> <<H>> <<H>> <<H>> <<H>> <<H>> <<H>> <<H>> <<H>> <<H>> <<H>> <<H>> <<H>> <<H>> <<H>> <<H>> <<H>> <<H>> <<H>> <<H>> <<H>> <<H>> <<H>> <<H>> <<H>> <<H>> <<H>> <<H>> <<H>>

Experiential-symbolic task

Data source: [29]

Number of experiments: 4
Number of participants: 346
Number of choices: 70608

Example prompt:
This experiment is composed of three parts.
In each round of the first part, you have to choose between one of two options represented by letters.
In a given pair, one option is, on average, more advantageous compared to the other.
You can win or lose the following outcomes: 1.0 points and -1.0 points.
In the second part, there will be two types of options.
The first type of option is represented by the letters you already encountered during the previous part.
Note that the options maintain the same odds of winning or losing a point as in the first part.
The second type of option is represented by an explicit description of the odds of winning or losing a point.
In each round of the third part, you will be presented with the options you met in the first and the second part.
This is the occasion to test your knowledge of each options's outcome.
You will be asked to indicate (in percentages), what are the odds that a given option makes you win a point.
You can choose an option by pressing its corresponding key.
Your goal for the first two parts is to maximize the amount of received points.
Your goal in the third part is to guess as accurately as possible.

You can choose between option Q and option L. You press <<Q>> and get 1.0 points. You would have gotten 1.0 points had you chosen option L instead.
You can choose between option Z and option H. You press <<Z>> and get 1.0 points. You would have gotten 1.0 points had you chosen option H instead.
You can choose between option D and option X. You press <<X>> and get 1.0 points. You would have gotten -1.0 points had you chosen option D instead.
You can choose between option U and option C. You press <<C>> and get 1.0 points. You would have gotten 1.0 points had you chosen option U instead.
You can choose between option U and option C. You press <<C>> and get -1.0 points. You would have gotten 1.0 points had you chosen option U instead.
You can choose between option D and option X. You press <<X>> and get 1.0

points. You would have gotten 1.0 points had you chosen option D instead.
You can choose between option Z and option H. You press <<Z>> and get 1.0 points. You would have gotten -1.0 points had you chosen option H instead.
You can choose between option Z and option H. You press <<Z>> and get 1.0 points. You would have gotten 1.0 points had you chosen option H instead.
You can choose between option D and option X. You press <<X>> and get -1.0 points. You would have gotten 1.0 points had you chosen option D instead.
You can choose between option D and option X. You press <<D>> and get 1.0 points. You would have gotten 1.0 points had you chosen option X instead.
You can choose between option Q and option L. You press <<Q>> and get 1.0 points. You would have gotten -1.0 points had you chosen option L instead.
You can choose between option D and option X. You press <<X>> and get -1.0 points. You would have gotten 1.0 points had you chosen option D instead.
You can choose between option D and option X. You press <<D>> and get 1.0 points. You would have gotten -1.0 points had you chosen option X instead.
You can choose between option Q and option L. You press <<Q>> and get -1.0 points. You would have gotten -1.0 points had you chosen option L instead.
You can choose between option Q and option L. You press <<L>> and get -1.0 points. You would have gotten 1.0 points had you chosen option Q instead.
You can choose between option Q and option L. You press <<Q>> and get 1.0 points. You would have gotten -1.0 points had you chosen option L instead.
You can choose between option Q and option L. You press <<Q>> and get -1.0 points. You would have gotten 1.0 points had you chosen option L instead.
You can choose between option U and option C. You press <<C>> and get -1.0 points. You would have gotten 1.0 points had you chosen option U instead.
You can choose between option U and option C. You press <<C>> and get -1.0 points. You would have gotten -1.0 points had you chosen option U instead.
You can choose between option Q and option L. You press <<Q>> and get 1.0 poi

Two-armed bandit

Data source: [30]

Number of experiments: 2
Number of participants: 80
Number of choices: 16000

Example prompt:
In this task, you have to repeatedly choose between two slot machines labeled U and P.
You can choose a slot machine by pressing its corresponding key.
When you select one of the machines, you will win or lose points.
Machine U will not always give you the same points when you select it again, but machine P will always give 0 points when you select it.
Your goal is to choose the slot machines that will give you the most points.
You will receive feedback about the outcome after making a choice.

You will play 20 games in total, each with a different pair of slot machines. Each game will consist of 10 trials.

Game 1:
You press <<U>> and get -1 points.
You press <<U>> and get 0 points.
You press <<U>> and get 2 points.
You press <<U>> and get -1 points.
You press <<U>> and get 1 points.
You press <<U>> and get -1 points.
You press <<U>> and get -1 points.
You press <<U>> and get -1 points.
You press <<U>> and get 1 points.
You press <<U>> and get 1 points.

Game 2:
You press <<U>> and get -1 points.
You press <<U>> and get -1 points.
You press <<U>> and get -2 points.
You press <<U>> and get 0 points.
You press <<U>> and get -1 points.
You press <<P>> and get 0 points.
You press <<P>> and get 0 points.
You press <<P>> and get 0 points.
You press <<P>> and get 0 points.
You press <<P>> and get 0 points.

Game 3:
You press <<U>> and get -2 points.
You press <<U>> and get -1 points.
You press <<P>> and get 0 points.
You press <<P>> and get 0 points.
You press <<P>> and get 0 points.
You press <<U>> and get -4 points.
You press <<P>> and get 0 points.
You press <<P>> and get 0 points.
You press <<P>> and get 0 points.
You press <<P>> and get 0 points.

Game 4:
You press <<U>> and get 2 points.
You press <<U>> and get 0 points.
You press <<U>> and get 2 points.
You press <<U>> and get 1 points.
You press <<U>> and get 2 points.

You press <<U>> and get -1 points.
You press <<U>> and get 0 points.
You press <<U>> and get 2 points.
You press <<U>> and get 1 points.
You press <<U>> and get 0 points.

Game 5:
You press <<U>> and get 0 points.
You press <<U>> and get 1 points.
You press <<U>> and get 1 points.
You press <<U>> and get 0 points.
You press <<U>> and get 2 points.
You press <<U>> and get 1 points.
You press <<U>> and get 2 points.
You press <<U>> and get 1 points.
You press <<U>> and get 2 points.
You press <<U>> and get 1 points.

Game 6:
You press <<U>> and get 3 points.
You press <<U>> and get 2 points.
You press <<U>> and get 0 points.
You press <<U>> and get 2 points.
You press <<U>> and get 2 points.
You press <<U>> and get 2 points.
You press <<U>> and get 1 points.
You press <<U>> and get 3 points.
You press <<U>> and get 2 points.
You press <<U>> and get 1 points.

Game 7:
You press <<U>> and get -1 points.
You press <<U>> and get -3 points.
You press <<P>> and get 0 points.
You press <<P>> and get 0 points.
You press <<P>> and get 0 points.
You press <<P>> and get 0 points.
You press <<P>> and get 0 points.
You press <<P>> and get 0 points.
You press <<P>> and get 0 points.
You press <<P>> and get 0 points.

Game 8:
You press <<U>> and get 0 points.
You press <<U>> and get -1 points.

You press <<P>> and get 0 points.
You press <<P>> and get 0 points.
You press <<P>> and get 0 points.
You press <<P>> and get 0 points.
You press <<P>> and get 0 points.
You press <<P>> and get 0 points.
You press <<P>> and get 0 points.
You press <<P>> and get 0 points.

Game 9:
You press <<U>> and get 2 points.
You press <<U>> and get 3 points.
You press <<U>> and get 4 points.
You press <<U>> and get 1 points.
You press <<U>> and get 1 points.
You press <<U>> and get 2 points.
You press <<U>> and get 2 points.
You press <<U>> and get 2 points.
You press <<U>> and get -1 points.
You press <<U>> and get 2 points.

Game 10:
You press <<U>> and get 1 points.
You press <<U>> and get 0 points.
You press <<U>> and get 0 points.
You press <<U>> and get 1 points.
You press <<U>> and get -1 points.
You press <<U>> and get -2 points.
You press <<U>> and get 1 points.
You press <<U>> and get 2 points.
You press <<U>> and get 1 points.
You pr

Conditional associative learning

Data source: [31]

Number of experiments: 1
Number of participants: 74
Number of choices: 40539

Example prompt:
You are presented with a series of stimuli, each associated with one of three possible responses.
Your goal is to learn which response is the correct one for each stimulus.
When a stimulus is presented, you can press one of three keys to respond.

The three responses available are S, F, and A.
After your response, you will receive feedback: 1 point for a correct response, or 0 points for an incorrect response.
The correct response for one stimulus does not inform you about the correct response for another stimulus.
You will play 13 games, each with a different mapping from stimuli to responses.

Game 1:
There are 6 different stimuli.
You see stimulus 1. You press <<S>> and get 0 points.
You see stimulus 0. You press <<F>> and get 0 points.
You see stimulus 4. You press <<A>> and get 1 points.
You see stimulus 5. You press <<S>> and get 0 points.
You see stimulus 3. You press <<F>> and get 1 points.
You see stimulus 3. You press <<F>> and get 1 points.
You see stimulus 4. You press <<A>> and get 1 points.
You see stimulus 2. You press <<S>> and get 0 points.
You see stimulus 1. You press <<S>> and get 0 points.
You see stimulus 5. You press <<S>> and get 0 points.
You see stimulus 2. You press <<S>> and get 0 points.
You see stimulus 0. You press <<A>> and get 0 points.
You see stimulus 3. You press <<F>> and get 1 points.
You see stimulus 3. You press <<F>> and get 1 points.
You see stimulus 5. You press <<A>> and get 1 points.
You see stimulus 1. You press <<A>> and get 0 points.
You see stimulus 4. You press <<A>> and get 1 points.
You see stimulus 1. You press <<S>> and get 0 points.
You see stimulus 0. You press <<S>> and get 1 points.
You see stimulus 5. You press <<S>> and get 0 points.
You see stimulus 4. You press <<A>> and get 1 points.
You see stimulus 2. You press <<S>> and get 0 points.
You see stimulus 0. You press <<S>> and get 1 points.
You see stimulus 2. You press <<S>> and get 0 points.
You see stimulus 3. You press <<F>> and get 1 points.
You see stimulus 1. You press <<A>> and get 0 points.
You see stimulus 5. You press <<A>> and get 1 points.
You see stimulus 4. You press <<A>> and get 1 points.
You see stimulus 0. You press <<S>> and get 1 points.
You see stimulus 1. You press <<S>> and get 0 points.
You see stimulus 3. You press <<F>> and get 1 points.
You see stimulus 4. You press <<A>> and get 1 points.
You see stimulus 0. You press <<S>> and get 1 points.
You see stimulus 2. You press <<S>> and get 0 points.
You see stimulus 2. You press <<S>> and get 0 points.
You see stimulus 3. You press <<F>> and get 1 points.

You see stimulus 5. You press <<S>> and get 0 points.
You see stimulus 1. You press <<A>> and get 0 points.
You see stimulus 0. You press <<F>> and get 0 points.
You see stimulus 5. You press <<F>> and get 0 points.
You see stimulus 4. You press <<A>> and get 1 points.
You see stimulus 4. You press <<A>> and get 1 points.
You see stimulus 1. You press <<A>> and get 0 points.
You see stimulus 3. You press <<F>> and get 1 points.
You see stimulus 2. You press <<F>> and get 1 points.
You see stimulus 2. You press <<F>> and get 1 points.
You see stimulus 3. You press <<F>> and get 1 points.
You see stimulus 0. You press <<F>> and get 0 points.
You see stimulus 1. You press <<F>> and get 1 points.
You see stimulus 5. You press <<F>> and get 0 points.
You see stimulus 0. You press <<F>> and get 0 points.
You see stimulus 5. You press <<F>> and get 0 points.
You see stimulus 4. You press <<A>> and get 1 points.
You see stimulus 2. You press <<A>> and get 0 points.
You see stimulus 0. You press <<F>> and get 0 points.
You see stimulus 0. You press <<F>> and get 0 points.
You see stimulus 1. You press <<A>> and get 0 points.
You see stimulus 2. You press <<A>> and get 0 points.
You see stimulus 1. You press <<A>> and get 0 points.
You see stimulus 4. You press <<A>> and get 1 points.
You see stimulus 4. You press <<A>> and get 1 points.
You see stimulus 2. You press <<S>> and get 0 points.
You see stimulus 5. You press <<S>> and get 0 points.
You see stimulus 5. You press <<F>> and get 0 points.

THINGS odd-one-out

Data source: [32]

Number of experiments: 1
Number of participants: 11122
Number of choices: 2611240

Example prompt:
You will be presented with triplets of objects, which will be assigned to the keys B, J, and K.
In each trial, please indicate which object you think is the odd one out by pressing the corresponding key.
In other words, please choose the object that is the least similar to the other two.

B: prune, J: nail polish, and K: diskette. You press <<K>>.
B: ladle, J: water bottle, and K: pug. You press <<K>>.

B: punch, J: hair, and K: lollipop. You press <<J>>.
B: oar, J: mug, and K: macaroni. You press <<B>>.
B: towel, J: hot tub, and K: mallet. You press <<K>>.
B: train set, J: hot-water bottle, and K: treasure. You press <<J>>.
B: shutter, J: cleaver, and K: toe. You press <<K>>.
B: straw, J: sweeper, and K: baton. You press <<J>>.
B: footprint, J: beehive, and K: skunk. You press <<B>>.
B: cash machine, J: thermostat, and K: mandolin. You press <<K>>.
B: orange, J: throne, and K: stir fry. You press <<J>>.
B: boy, J: burrito, and K: microscope. You press <<J>>.
B: pheasant, J: sponge, and K: orchid. You press <<J>>.
B: forklift, J: clipper, and K: hip. You press <<J>>.
B: candelabra, J: beard, and K: glue. You press <<J>>.
B: raccoon, J: hammer, and K: roulette wheel. You press <<B>>.
B: wing, J: beanie, and K: girl. You press <<B>>.
B: piggy bank, J: footrest, and K: sandal. You press <<B>>.
B: knee, J: cornhusk, and K: tuning fork. You press <<B>>.
B: anklet, J: bedpost, and K: ice cube. You press <<B>>.
B: mannequin, J: stove, and K: coin. You press <<K>>.
B: tortellini, J: cantaloupe, and K: sequin. You press <<K>>.
B: coffee filter, J: fingerprint, and K: rose. You press <<B>>.
B: porcupine, J: christmas tree, and K: corkscrew. You press <<B>>.
B: freezer, J: coat rack, and K: puffin. You press <<K>>.
B: maggot, J: mouth, and K: stockings. You press <<B>>.
B: soap, J: hot-water bottle, and K: knitting needle. You press <<K>>.
B: mosquito net, J: baklava, and K: beanbag. You press <<J>>.
B: skewer, J: baklava, and K: propeller. You press <<K>>.
B: nail polish, J: goose, and K: pizza. You press <<J>>.
B: face mask, J: cinnamon, and K: toilet paper. You press <<B>>.
B: bag, J: eel, and K: trampoline. You press <<J>>.
B: lightbulb, J: moose, and K: curling iron. You press <<J>>.
B: dumbwaiter, J: jigsaw puzzle, and K: lamb. You press <<K>>.
B: eyeliner, J: shopping basket, and K: flipper. You press <<K>>.
B: bowtie, J: wooden leg, and K: kangaroo. You press <<K>>.
B: puffin, J: wire cutters, and K: battery. You press <<B>>.
B: toilet, J: rack, and K: star fruit. You press <<K>>.
B: stingray, J: cork, and K: fire pit. You press <<B>>.
B: bun, J: snow, and K: tinsel. You press <<J>>.
B: almond, J: trailer, and K: paper. You press <<B>>.
B: anteater, J: chalice, and K: wedge. You press <<B>>.
B: scone, J: pie, and K: ant. You press <<K>>.
B: heater, J: aircraft carrier, and K: joystick. You press <<B>>.
B: comic book, J: playing card, and K: organ. You press <<K>>.
B: flag, J: measuring cup, and K: strawberry. You press <<B>>.
B: bunkbed, J: tractor, and K: windshield. You press <<B>>.

B: aircraft carrier, J: prism, and K: turban. You press <<K>>.
B: egg, J: scrambled egg, and K: doorknocker. You press <<K>>.
B: microscope, J: stained glass, and K: strainer. You press <<J>>.
B: polygraph, J: hairdryer, and K: harness. You press <<J>>.
B: chip, J: iguana, and K: hedge. You press <<B>>.
B: tick, J: binder, and K: shoelace. You press <<B>>.
B: nut, J: yogurt, and K: jug. You press <<K>>.
B: hairpin, J: giraffe, and K: fur coat. You press <<B>>.
B: lasagna, J: statue, and K: bookshelf. You press <<B>>.
B: grill, J: catapult, and K: sonogram. You press <<K>>.
B: fondue, J: pill, and K: firewood. You press <<K>>.
B: sunroof, J: onion, and K: flan. You press <<B>>.
B: wreck, J: bungee, and K: cockroach. You press <<K>>.

Multi-attribute decision-making

Data source: [1]

Number of experiments: 1
Number of participants: 73
Number of choices: 7008

Example prompt:
You are repeatedly presented with two options, labeled A and R.
Each option represents a fictitious product and you have to infer which product is superior in terms of quality.
You select a product by pressing the corresponding key.
For each decision, you are provided with four expert ratings (with 1 representing a positive and 0 representing a negative rating).
The four experts differ in their validity.
The ratings of experts are given in descending order of their validity (having validities of 90%, 80%, 70%, and 60%).

Product A ratings: [0 1 1 1]. Product R ratings: [1 0 0 1]. You press <<A>>.
Product A ratings: [1 1 1 1]. Product R ratings: [0 0 1 1]. You press <<A>>.
Product A ratings: [1 0 0 0]. Product R ratings: [0 0 0 1]. You press <<A>>.
Product A ratings: [1 1 1 0]. Product R ratings: [0 0 1 0]. You press <<A>>.
Product A ratings: [0 1 1 1]. Product R ratings: [1 1 1 0]. You press <<R>>.
Product A ratings: [0 1 0 1]. Product R ratings: [1 1 0 0]. You press <<R>>.
Product A ratings: [0 0 1 1]. Product R ratings: [1 0 1 0]. You press <<R>>.
Product A ratings: [1 0 0 1]. Product R ratings: [0 1 1 1]. You press <<R>>.
Product A ratings: [0 1 1 1]. Product R ratings: [1 0 0 1]. You press <<A>>.
Product A ratings: [0 1 1 1]. Product R ratings: [1 1 1 0]. You press <<R>>.
Product A ratings: [1 0 1 0]. Product R ratings: [0 0 1 1]. You press <<A>>.
Product A ratings: [1 1 0 0]. Product R ratings: [0 1 0 1]. You press <<A>>.
Product A ratings: [1 0 0 0]. Product R ratings: [0 1 1 0]. You press <<R>>.

Product A ratings: [1 0 0 1]. Product R ratings: [0 1 1 1]. You press <<R>>.
Product A ratings: [1 1 1 1]. Product R ratings: [0 0 1 1]. You press <<A>>.
Product A ratings: [1 0 0 0]. Product R ratings: [0 0 0 1]. You press <<A>>.
Product A ratings: [1 1 0 1]. Product R ratings: [0 0 0 1]. You press <<A>>.
Product A ratings: [1 0 0 1]. Product R ratings: [0 1 1 1]. You press <<R>>.
Product A ratings: [0 1 0 1]. Product R ratings: [1 1 0 0]. You press <<R>>.
Product A ratings: [1 0 1 1]. Product R ratings: [0 0 0 0]. You press <<A>>.
Product A ratings: [1 0 0 0]. Product R ratings: [0 0 0 1]. You press <<A>>.
Product A ratings: [0 1 1 1]. Product R ratings: [1 1 1 0]. You press <<R>>.
Product A ratings: [0 1 1 0]. Product R ratings: [1 0 0 0]. You press <<A>>.
Product A ratings: [1 1 1 0]. Product R ratings: [0 1 1 1]. You press <<A>>.
Product A ratings: [0 0 0 0]. Product R ratings: [1 1 0 0]. You press <<R>>.
Product A ratings: [0 1 1 1]. Product R ratings: [1 0 0 1]. You press <<A>>.
Product A ratings: [0 0 0 0]. Product R ratings: [1 1 0 0]. You press <<R>>.
Product A ratings: [1 0 0 0]. Product R ratings: [0 1 1 0]. You press <<R>>.
Product A ratings: [0 0 0 0]. Product R ratings: [1 1 0 0]. You press <<R>>.
Product A ratings: [0 1 1 1]. Product R ratings: [1 0 0 1]. You press <<A>>.
Product A ratings: [0 0 0 1]. Product R ratings: [1 0 0 0]. You press <<R>>.
Product A ratings: [1 0 0 0]. Product R ratings: [0 1 1 0]. You press <<R>>.
Product A ratings: [1 1 0 0]. Product R ratings: [0 0 0 0]. You press <<A>>.
Product A ratings: [1 1 0 0]. Product R ratings: [0 1 0 1]. You press <<A>>.
Product A ratings: [1 0 0 0]. Product R ratings: [0 0 0 1]. You press <<A>>.
Product A ratings: [0 0 0 1]. Product R ratings: [1 0 0 0]. You press <<R>>.
Product A ratings: [1 0 1 0]. Product R ratings: [0 0 1 1]. You press <<A>>.
Product A ratings: [1 1 0 0]. Product R ratings: [0 0 0 0]. You press <<A>>.
Product A ratings: [0 0 1 1]. Product R ratings: [1 0 1 0]. You press <<R>>.
Product A ratings: [1 0 0 0]. Product R ratings: [0 1 1 0]. You press <<R>>.
Product A ratings: [1 1 1 0]. Product R ratings: [0 0 1 0]. You press <<A>>.
Product A ratings: [1 0 0 1]. Product R ratings: [0 1 1 1]. You press <<R>>.
Product A ratings: [1 0 1 0]. Product R ratings: [0 0 1 1]. You press <<A>>.
Product A ratings: [0 1 1 0]. Product R ratings: [1 0 0 0]. You press <<A>>.
Product A ratings: [1 1 0 0]. Product R ratings: [0 1 0 1]. You press <<A>>.
Product A ratings: [0 1 1 0]. Product R ratings: [1 0 0 0]. You press <<A>>.
Product A ratings: [1 0 0 0

Grammar judgement

Data source: [33]

Number of experiments: 1
Number of participants: 3192
Number of choices: 89376

Example prompt:
You're about to answer a set of 20 questions about grammar. How many of the 20 questions do you think you will answer correctly?

You say <<4>>.
Compared to other participants in this study, how well do you think you will do? Marking 90% means you will do better than 90% of participants, marking 10% means you will do better than only 10%, and marking 50% means that you will perform better than half of the participants.
You say <<50>>%.
On a scale of 0 to 10, how difficult is recognizing correct grammar for the average participant?
You say <<6>>.
On a scale of 0 to 10, how difficult is recognizing correct grammar for you?
You say <<6>>.

You will now see twenty questions.
In each question, some part of each sentence is in square brackets.
Five choices for rephrasing that part follow each sentence; one choice repeats the original, and the other four are different.
Your task is to use the buttons J, E, V, H, and G to select the grammatically correct choice.

Q1. The school-age child faces a formidable task when during the first few years of classroom experiences [he or she is expected to master the printed form of language.]
The choices are:
V: he or she expects to master the printed form of language.
E: he or she is expected to master the printed form of language.
H: he or she faces expectations of mastering the printed form of language.
J: mastery of the printed form of language is expected of him or her.
G: mastery of print is expected by his or her teacher.
You press <<E>>.

Q2. He came to the United States as a young [man, he found] a job as a coal miner.
The choices are:
H: man, he found
G: man and found
E: man and there he was able to find
V: man and then finding
J: man and had found
You press <<H>>.

Q3. To a large degree, [poetry, along with all the other arts, is] a form of imitation.
The choices are:
E: poetry, along with all the other arts, is
V: poetry along with all the other arts is
J: poetry, along with all the other arts, are
G: poetry, and other arts, is
H: poetry and art are

You press <<V>>.

Q4. Delegates to the political convention found [difficulty to choose] a candidate from among the few nominated.
The choices are:
E: difficulty to choose
G: it difficult in making the choice of
H: it difficult to choose
V: choosing difficult when selecting
J: making a choice difficult in selecting
You press <<E>>.

Q5. Reading in any language can be viewed as a developmental task much the same as learning to walk, to cross the street independently, to care for one's possessions, or [accepting responsibility for one's own decisions.]
The choices are:
J: accepting responsibility for one's own decisions.
V: accepting one's own decisions responsibly.
H: to accept responsibility for one's own decisions.
E: accepting responsibility and making one's own decisions.
G: to make one's own decisions.
You press <<G>>.

Q6. Sea forests of giant kelp, which fringe only one coastline in the Northern Hemisphere, [is native to shores] throughout the Southern Hemisphere.
The choices are:
E: is native to shores
V: is native to most shores
G: are native only in shores
J: are native
H: are native to shores
You press <<J>>.

Q7. Taking an occasional respite between chapters or assignments is more desirable [than a long, continuous period of study.
The choices are:
E: than a long, continuous period of study.
G: than a period of long, continuous study.
V: than a long period of continuous study.
J: than studying for a long, continuous period.
H: than a study period long and continuous.
You press <<V>>.

Q8. Like so many characters in Russian fiction, [Crime and Punishment exhibits] a behavior so foreign to the American temperament that many readers find the story rather incredible.

The choices are:
J: Crime and Punishment exhibits
H: those in Crime and Punishment exhibit
G: those in Crime and Punishment exhibits
V: often exhibiting
E: characterized by
You press <<G>>.

Q9. Don Quixote provides a cross section of Spanish life, thought, and [portrays the feelings of many Spaniards] at the end of the chivalric age.
The choices are:
H: portrays th

Two-step task

Data source: [34]

Number of experiments: 2
Number of participants: 181
Number of choices: 52861

Example prompt:
Each day you will either be presented with spaceships P and F or with spaceships Z and J.
These spaceships will take you to two different planets L and Q.
You can take a spaceship by pressing the corresponding key.
Each planet has one alien on it and each alien has its own space treasure mine.
When you arrive at a planet, you will ask the alien for space treasure from its mine.
When you ask the alien, you will find out whether you got space treasure.
However, sometimes the alien will not bring up any treasure.
The quality of each alien's mine will change during the game.
Before you choose a spaceship, you will be told whether there is a treasure multiplier.
If there is a treasure multiplier, you will receive 5 times the amount of treasure you will find.
Your goal is to get as much treasure as possible over the next 200 days.

There is no treasure multiplier. You are presented with spaceships F and P. You press <<P>>. You end up on planet L. You find 7 pieces of space treasure. You receive 7 pieces of space treasure.
There is no treasure multiplier. You are presented with spaceships F and P. You press <<F>>. You end up on planet Q. You find 0 pieces of space treasure. You receive 0 pieces of space treasure.
There is no treasure multiplier. You are presented with spaceships J and Z. You press <<Z>>. You end up on planet L. You find 7 pieces of space treasure. You receive 7 pieces of space treasure.
There is no treasure multiplier. You are presented with spaceships Z and J. You press

<<Z>>. You end up on planet L. You find 7 pieces of space treasure. You receive 7 pieces of space treasure.
There is no treasure multiplier. You are presented with spaceships Z and J. You press <<Z>>. You end up on planet L. You find 8 pieces of space treasure. You receive 8 pieces of space treasure.
There is a treasure multiplier. You are presented with spaceships F and P. You press <<P>>. You end up on planet L. You find 6 pieces of space treasure. You receive 30 pieces of space treasure.
There is a treasure multiplier. You are presented with spaceships F and P. You press <<P>>. You end up on planet L. You find 8 pieces of space treasure. You receive 40 pieces of space treasure.
There is no treasure multiplier. You are presented with spaceships J and Z. You press <<Z>>. You end up on planet L. You find 9 pieces of space treasure. You receive 9 pieces of space treasure.
There is no treasure multiplier. You are presented with spaceships P and F. You press <<P>>. You end up on planet L. You find 8 pieces of space treasure. You receive 8 pieces of space treasure.
There is a treasure multiplier. You are presented with spaceships J and Z. You press <<Z>>. You end up on planet L. You find 9 pieces of space treasure. You receive 45 pieces of space treasure.
There is a treasure multiplier. You are presented with spaceships P and F. You press <<P>>. You end up on planet L. You find 9 pieces of space treasure. You receive 45 pieces of space treasure.
There is no treasure multiplier. You are presented with spaceships J and Z. You press <<Z>>. You end up on planet L. You find 8 pieces of space treasure. You receive 8 pieces of space treasure.
There is a treasure multiplier. You are presented with spaceships J and Z. You press <<Z>>. You end up on planet L. You find 9 pieces of space treasure. You receive 45 pieces of space treasure.
There is no treasure multiplier. You are presented with spaceships Z and J. You press <<Z>>. You end up on planet L. You find 5 pieces of space treasure. You receive 5 pieces of space treasure.
There is no treasure multiplier. You are presented with spaceships Z and J. You press <<Z>>. You end up on planet L. You find 6 pieces of space treasure. You receive 6 pieces of space treasure.
There is a treasure multiplier. You are presented with spaceships P and F. You press <<P>>. You end up on planet L. You find 8 pieces of space treasure. You receive 40 pieces of space treasure.
There is no treasure multiplier. You are presented with spaceships J and Z. You press <<Z>>. You end up on planet L. You find 7 pieces of space treasure.

Two-step task

Data source: [35]

Number of experiments: 2

Number of participants: 367
Number of choices: 67041

Example prompt:
Each day you will either be presented with spaceships G and S or with spaceships T and N.
These spaceships will take you to two different planets R and Z.
You can take a spaceship by pressing the corresponding key.
Each planet has one alien on it and each alien has its own space treasure mine.
When you arrive at a planet, you will ask the alien for space treasure from its mine.
When you ask the alien, you will find out whether you got space treasure.
However, sometimes the alien's mine will dig up antimatter.
Antimatter is bad because each piece will destroy a piece of space treasure, reducing the total amount of treasure that you have.
The quality of each alien's mine will change during the game.
Your goal is to get as much treasure and as little antimatter as possible over the next 125 days.

You are presented with spaceships N and T. You press <<T>>. You end up on planet R. You find 1 pieces of antimatter.
You are presented with spaceships T and N. You do not respond in time on this day. You do not go to any planet. You find nothing.
You are presented with spaceships N and T. You press <<N>>. You end up on planet Z. You find 4 pieces of space treasure.
You are presented with spaceships T and N. You press <<N>>. You end up on planet Z. You find 4 pieces of space treasure.
You are presented with spaceships S and G. You press <<S>>. You end up on planet Z. You find 5 pieces of space treasure.
You are presented with spaceships T and N. You press <<N>>. You end up on planet Z. You find 3 pieces of space treasure.
You are presented with spaceships N and T. You press <<N>>. You end up on planet Z. You find 4 pieces of space treasure.
You are presented with spaceships T and N. You press <<N>>. You end up on planet Z. You find 2 pieces of space treasure.
You are presented with spaceships S and G. You press <<S>>. You end up on planet Z. You find 1 pieces of space treasure.
You are presented with spaceships T and N. You press <<N>>. You end up on planet Z. You find 3 pieces of antimatter.
You are presented with spaceships T and N. You press <<T>>. You end up on planet R. You find 2 pieces of space treasure.
You are presented with spaceships G and S. You press <<G>>. You end up on planet R. You find 4 pieces of space treasure.
You are presented with spaceships S and G. You press <<G>>. You end up on planet R. You find 2 pieces of space treasure.
You are presented with spaceships T and N. You press <<T>>. You end up on

planet R. You find 3 pieces of space treasure.
You are presented with spaceships T and N. You press <<T>>. You end up on planet R. You find 5 pieces of space treasure.
You are presented with spaceships N and T. You press <<T>>. You end up on planet R. You find 4 pieces of space treasure.
You are presented with spaceships S and G. You press <<G>>. You end up on planet R. You find 4 pieces of space treasure.
You are presented with spaceships S and G. You press <<G>>. You end up on planet R. You find nothing.
You are presented with spaceships T and N. You press <<T>>. You end up on planet R. You find 3 pieces of antimatter.
You are presented with spaceships T and N. You press <<N>>. You end up on planet Z. You find 2 pieces of space treasure.
You are presented with spaceships S and G. You press <<S>>. You end up on planet Z. You find 2 pieces of space treasure.
You are presented with spaceships S and G. You press <<S>>. You end up on planet Z. You find 4 pieces of space treasure.
You are presented with spaceships S and G. You press <<S>>. You end up on planet Z. You find 4 pieces of space treasure.
You are presented with spaceships N and T. You press <<N>>. You end up on planet Z. You find 4 pieces of space treasure.
You are presented with spaceships N and T. You press <<N>>. You end up on planet Z. You find 5 pieces of space treasure.
You are presented with spaceships S and G. You press <<S>>. You end up on planet Z. You find 3 pieces of space treasure.
You are presented with spaceships G and S. You press <<S>>. You end up on planet Z. You find 4 pieces of space treasure.
You are presented with spaceships S and G. Yo

Risky choice

Data source: [36]

Number of experiments: 1
Number of participants: 1755
Number of choices: 499728

Example prompt:
You will play multiple rounds of a gambling game.
In each round, you will be presented with 6 different gambles labeled: Q, N, E, S, H, and K.
You will have to choose one of the gambles and receive a payoff for doing so.
The payoff you receive depends on both the gamble you choose and also the color of a ball we pull out of a jar with 100 colored balls.
There are a different number of balls of each color on every round.
The colors with more balls are more likely to be chosen.

Before making your choice, you may check how much different gambles are worth for different ball colors.
Each time you check a gamble will cost you 4 points.
To choose or check a gamble, first press the corresponding key, followed by typing "stop" (for choosing) or the ball color you would like to check.

A new round begins.
There are 50 pink balls, 19 red balls, 15 black balls, and 16 maroon balls.
You press <<Q>> and then type <<pink>>. The payoff for this combination would be -105 points.
You press <<E>> and then type <<pink>>. The payoff for this combination would be 47 points.
You press <<H>> and then type <<pink>>. The payoff for this combination would be 91 points.
You press <<H>> and then type <<stop>>. A maroon ball is chosen, and you earn -16 points.

A new round begins.
There are 2 pink balls, 47 red balls, 14 black balls, and 37 maroon balls.
You press <<Q>> and then type <<red>>. The payoff for this combination would be -168 points.
You press <<E>> and then type <<red>>. The payoff for this combination would be 209 points.
You press <<H>> and then type <<red>>. The payoff for this combination would be 22 points.
You press <<E>> and then type <<stop>>. A maroon ball is chosen, and you earn 25 points.

A new round begins.
There are 13 pink balls, 1 red balls, 47 black balls, and 39 maroon balls.
You press <<Q>> and then type <<black>>. The payoff for this combination would be -101 points.
You press <<E>> and then type <<black>>. The payoff for this combination would be -98 points.
You press <<H>> and then type <<black>>. The payoff for this combination would be 32 points.
You press <<H>> and then type <<stop>>. A maroon ball is chosen, and you earn -85 points.

A new round begins.
There are 7 pink balls, 15 red balls, 76 black balls, and 2 maroon balls.
You press <<N>> and then type <<black>>. The payoff for this combination would be -108 points.
You press <<Q>> and then type <<black>>. The payoff for this combination would be -191 points.

You press <<S>> and then type <<black>>. The payoff for this combination would be -222 points.
You press <<K>> and then type <<stop>>. A black ball is chosen, and you earn -90 points.

A new round begins.
There are 17 pink balls, 54 red balls, 16 black balls, and 13 maroon balls.
You press <<Q>> and then type <<red>>. The payoff for this combination would be 138 points.
You press <<N>> and then type <<red>>. The payoff for this combination would be 171 points.
You press <<E>> and then type <<red>>. The payoff for this combination would be -197 points.
You press <<N>> and then type <<stop>>. A red ball is chosen, and you earn 171 points.

A new round begins.
There are 1 pink balls, 38 red balls, 25 black balls, and 36 maroon balls.
You press <<N>> and then type <<red>>. The payoff for this combination would be -124 points.
You press <<E>> and then type <<red>>. The payoff for this combination would be -158 points.
You press <<S>> and then type <<red>>. The payoff for this combination would be -114 points.
You press <<H>> and then type <<stop>>. A black ball is chosen, and you earn 338 points.

A new round begins.
There are 37 pink balls, 44 red balls, 17 black balls, and 2 maroon balls.
You press <<Q>> and then type <<red>>. The payoff for this combination would be -198 points.
You press <<S>> and then type <<red>>. The payoff for this combination would be 39 points.
You press <<H>> and then type <<red>>. The payoff for this combination would be -63 points.
You press <<E>> and then type <<stop>>. A red ball is chosen, and you earn 51 points.

A new round begins.
There are 12 pink balls, 32 red balls, 45

Tile-revealing task

Data source: [37]

Number of experiments: 1

Number of participants: 707
Number of choices: 321738

Example prompt:
You are playing a game where you are revealing patterns on a binary grid.
Each turn you will be presented with a 7x7 grid of tiles.
If you give the location on the grid with an ”X” on it, then it will either turn into a ”1” or a ”0”.
Please give the location as a [row, column] pair where both row and column take values between 0 and 6.
Do not give the location on the grid where there is already a ”1” or a ”0”.
Your job is to reveal as many 1s as possible while revealing as little 0s as possible.
The game ends when you have revealed all 1s on the grid.
You will have 25 boards to complete, each with a different underlying pattern.

You are currently solving board 1.
The board state is now:
XXXXXXX
XXXXXXX
XXXXXXX
XXX1XXX
XXXXXXX
XXXXXXX
XXXXXXX
You choose <<[2, 2]>> and get a miss!

The board state is now:
XXXXXXX
XXXXXXX
XX0XXXX
XXX1XXX
XXXXXXX
XXXXXXX
XXXXXXX
You choose <<[2, 4]>> and get a miss!

The board state is now:
XXXXXXX
XXXXXXX
XX0X0XX
XXX1XXX
XXXXXXX
XXXXXXX
XXXXXXX
You choose <<[2, 3]>> and get a hit!

The board state is now:
XXXXXXX
XXXXXXX
XX010XX
XXX1XXX
XXXXXXX
XXXXXXX
XXXXXXX
You choose <<[1, 3]>> and get a miss!

The board state is now:
XXXXXXX
XXX0XXX
XX010XX
XXX1XXX
XXXXXXX
XXXXXXX
XXXXXXX
You choose <<[3, 4]>> and get a hit!

The board state is now:
XXXXXXX
XXX0XXX
XX010XX
XXX11XX
XXXXXXX
XXXXXXX
XXXXXXX
You choose <<[3, 2]>> and get a hit!

The board state is now:
XXXXXXX
XXX0XXX
XX010XX
XX111XX
XXXXXXX
XXXXXXX
XXXXXXX
You choose <<[3, 5]>> and get a hit!

The board state is now:
XXXXXXX
XXX0XXX
XX010XX
XX1111X
XXXXXXX

XXXXXXX
XXXXXXX
You choose <<[3, 6]>> and get a hit!

The board state is now:
XXXXXXX
XXX0XXX
XX010XX
XX11111
XXXXXXX
XXXXXXX
XXXXXXX
You choose <<[3, 1]>> and get a hit!

You are currently solving board 2.
The board state is now:
XXXXXXX
X1XXXXX
XXXXXXX
XXXXXXX
XXXXXXX
XXXXXXX
XXXXXXX
You choose <<[2, 1]>> and get a miss!

The board state is now:
XXXXXXX
X1XXXXX
X0XXXXX
XXXXXXX
XXXXXXX
XXXXXXX
XXXXXXX
You choose <<[0, 1]>> and get a miss!

The board state is now:
X0XXXXX
X1XXXXX
X0XXXXX
XXXXXXX
XXXXXXX
XXXXXXX
XXXXXXX
You choose <<[1, 2]>> and get a hit!

The board state is now:

X0XXXXX
X11XXXX
X0XXXXX
XXXXXXX
XXXXXXX
XXXXXXX
XXXXXXX
You choose <<[1, 0]>> and get a hit!

The board state is now:
X0XXXXX
111XXXX
X0XXXXX
XXXXXXX
XXXXXXX
XXXXXXX
XXXXXXX
You choose <<[1, 3]>> and get a hit!

The board state is now:
X0XXXXX
1111XXX
X0XXXXX
XXXXXXX
XXXXXXX
XXXXXXX
XXXXXXX
You choose <<[1, 4]>> and get a hit!

The board state is now:
X0XXXXX
11111XX
X0XXXXX
XXXXXXX
XXXXXXX
XXXXXXX
XXXXXXX
You choose <<[1, 5]>> and get a miss!

The board state is now:
X0XXXXX
111110X
X0XXXXX
XXXXXXX
XXXXXXX
XXXXXXX

XXXXXXX
You choose <<[0, 0]>> and get a miss!

The board state is now:
00XXXXX
111110X
X0XXXXX
XXXXXXX
XXXXXXX
XXXXXXX
XXXXXXX
You choose <<[2, 2]>> and get a miss!

The board state is now:
00XXXXX
111110X
X00XXXX
XXXXXXX
XXXXXXX
XXXXXXX
XXXXXXX
You choose <<[2, 3]>> and get a hit!

The board state is now:
00XXXXX
111110X
X001XXX
XXXXXXX
XXXXXXX
XXXXXXX
XXXXXXX
You choose <<[3, 3]>> and get a hit!

The board state is now:
00XXXXX
111110X
X001XXX
XXX1XXX
XXXXXXX
XXXXXXX
XXXXXXX
You choose <<[0, 3]>> and get a hit!

The board state is now:
00X1XXX
111110X

X001XXX
XXX1XXX
XXXXXXX
XXXXXXX
XXXXXXX
You choose <<[4, 3]>> and get a hit!

You are currently solving board 3.
The board state is now:
XXXXXXX
XXXXXXX
XXXXXXX
XXXXXXX
XXXXXXX
XXX1XXX
XXXXXXX
You choose <<[4, 2]>> and get a hit!

The board state is now:
XXXXXXX
XXXXXXX
XXXXXXX
XXXXXXX
XX1XXXX
XXX1XXX
XXXXXXX
You choose <<[3, 1]>> and get a hit!

The board state is now:
XXXXXXX
XXXXXXX
XXXXXXX
X1XXXXX
XX1XXXX
XXX1XXX
XXXXXXX
You choose <<[2, 0]>> and get a hit!

The board state is now:
XXXXXXX
XXXXXXX
1XXXXXX
X1XXXXX
XX1XXXX
XXX1XXX
XXXXXXX

You choose <<[1, 1]>> and get a miss!

The board state is now:
XXXXXXX
X0XXXXX
1XXXXXX
X1XXXXX
XX1XXXX
XXX1XXX
XXXXXXX
You choose <<[2, 2]>> and get a miss!

The board state is now:
XXXXXXX
X0XXXXX
1X0XXXX
X1XXXXX
XX1XXXX
XXX1XXX
XXXXXXX
You choose <<[3, 3]>> and get a hit!

The board state is now:
XXXXXXX
X0XXXXX

Probabilistic instrumental learning

Data source: [38]

Number of experiments: 2
Number of participants: 77
Number of choices: 7392

Example prompt:
You are going to visit four different casinos (named 1, 2, 3, and 4) 24 times each.
Each casino owns two slot machines that return either 0 or 0.5 points stochastically with different probabilities.
You can play one of the machines in order to win points by pressing the corresponding key.
Your goal is to maximize the sum of received points within all visits.

You go to casino 3. You can choose between machines B and C. You press <<B>> and receive 0.0 points.
You go to casino 4. You can choose between machines P and T. You press <<T>> and receive 0.5 points.

You go to casino 4. You can choose between machines P and T. You press <<T>> and receive 0.5 points.
You go to casino 3. You can choose between machines B and C. You press <<B>> and receive 0.0 points.
You go to casino 1. You can choose between machines F and I. You press <<I>> and receive 0.0 points.
You go to casino 1. You can choose between machines F and I. You press <<F>> and receive 0.0 points.
You go to casino 2. You can choose between machines L and J. You press <<L>> and receive 0.5 points.
You go to casino 3. You can choose between machines B and C. You press <<B>> and receive 0.0 points.
You go to casino 4. You can choose between machines P and T. You press <<T>> and receive 0.0 points.
You go to casino 3. You can choose between machines B and C. You press <<C>> and receive 0.0 points.
You go to casino 4. You can choose between machines P and T. You press <<P>> and receive 0.5 points.
You go to casino 1. You can choose between machines F and I. You press <<F>> and receive 0.5 points.
You go to casino 2. You can choose between machines L and J. You press <<L>> and receive 0.5 points.
You go to casino 2. You can choose between machines L and J. You press <<L>> and receive 0.5 points.
You go to casino 1. You can choose between machines F and I. You press <<F>> and receive 0.0 points.
You go to casino 2. You can choose between machines L and J. You press <<J>> and receive 0.5 points.
You go to casino 1. You can choose between machines F and I. You press <<I>> and receive 0.0 points.
You go to casino 1. You can choose between machines F and I. You press <<F>> and receive 0.0 points.
You go to casino 2. You can choose between machines L and J. You press <<J>> and receive 0.0 points.
You go to casino 2. You can choose between machines L and J. You press <<L>> and receive 0.5 points.
You go to casino 3. You can choose between machines B and C. You press <<B>> and receive 0.5 points.
You go to casino 3. You can choose between machines B and C. You press <<B>> and receive 0.0 points.
You go to casino 2. You can choose between machines L and J. You press <<L>> and receive 0.5 points.
You go to casino 3. You can choose between machines B and C. You press <<C>> and receive 0.5 points.
You go to casino 2. You can choose between machines L and J. You press <<L>>

and receive 0.0 points.
You go to casino 1. You can choose between machines F and I. You press <<I>> and receive 0.0 points.
You go to casino 4. You can choose between machines P and T. You press <<P>> and receive 0.0 points.
You go to casino 4. You can choose between machines P and T. You press <<T>> and receive 0.5 points.
You go to casino 4. You can choose between machines P and T. You press <<T>> and receive 0.5 points.
You go to casino 1. You can choose between machines F and I. You press <<I>> and receive 0.5 points.
You go to casino 4. You can choose between machines P and T. You press <<P>> and receive 0.5 points.
You go to casino 3. You can choose between machines B and C. You press <<C>> and receive 0.5 points.
You go to casino 2. You can choose between machines L and J. You press <<L>> and receive 0.5 points.
You go to casino 3. You can choose between machines B and C. You press <<C>> and receive 0.5 points.
You go to casino 4. You can choose between machines P and T. You press <<P>> and receive 0.5 points.
You go to casino 3. You can choose between machines B and C. You press <<C>> and receive 0.5 points.
You go to casino 4. You can choose between machines P and T. You press <<P>> and receive 0.5 point

Medin categorization

Data source: [39]

Number of experiments: 2
Number of participants: 228
Number of choices: 37848

Example prompt:
You will observe a series of objects, one at a time.
The objects differ along three binary dimensions: shape (square vs. triangle), size (1.50 inch vs. 0.75 inch), and shading (black vs. white).
Each dimension is indicated by the three digits, for example, '121' means a square, 0.75 inch, black object.
Based on some combination of the three dimensions, each object belongs to one of two categories, W or N.
You have to assign each object to one of the two categories by pressing the corresponding key.
If your choice is correct, you get a point, otherwise you lose a point.
Your goal is to get as many points as possible.

At some point, you begin a 'test block' in which you will see eight objects.
Here, you have to assign each object to one of the two categories as before.
Furthermore, you have to rate how typical the object is for the category you chose, on a scale from 1 to 9.
1 means 'not at all typical', and 9 means 'most typical'.

You see the image 112, press <<W>> and get 1 points.
You see the image 121, press <<N>> and get 0 points.
You see the image 212, press <<W>> and get 0 points.
You see the image 211, press <<N>> and get 0 points.
You see the image 221, press <<W>> and get 0 points.
You see the image 122, press <<W>> and get 0 points.
You see the image 122, press <<N>> and get 1 points.
You see the image 221, press <<W>> and get 0 points.
You see the image 121, press <<N>> and get 0 points.
You see the image 212, press <<N>> and get 1 points.
You see the image 211, press <<N>> and get 0 points.
You see the image 112, press <<W>> and get 1 points.
You see the image 112, press <<W>> and get 1 points.
You see the image 212, press <<N>> and get 1 points.
You see the image 221, press <<W>> and get 0 points.
You see the image 211, press <<N>> and get 0 points.
You see the image 121, press <<N>> and get 0 points.
You see the image 122, press <<W>> and get 0 points.
You see the image 212, press <<N>> and get 1 points.
You see the image 221, press <<W>> and get 0 points.
You see the image 211, press <<N>> and get 0 points.
You see the image 121, press <<N>> and get 0 points.
You see the image 112, press <<W>> and get 1 points.
You see the image 122, press <<W>> and get 0 points.
You see the image 122, press <<N>> and get 1 points.
You see the image 112, press <<W>> and get 1 points.
You see the image 121, press <<N>> and get 0 points.
You see the image 221, press <<N>> and get 1 points.
You see the image 212, press <<N>> and get 1 points.
You see the image 211, press <<N>> and get 0 points.
You see the image 112, press <<W>> and get 1 points.
You see the image 212, press <<N>> and get 1 points.
You see the image 122, press <<W>> and get 0 points.
You see the image 211, press <<N>> and get 0 points.
You see the image 221, press <<W>> and get 0 points.
You see the image 121, press <<N>> and get 0 points.
You see the image 122, press <<N>> and get 1 points.
You see the image 212, press <<W>> and get 0 points.
You see the image 211, press <<N>> and get 0 points.

You see the image 221, press <<N>> and get 1 points.
You see the image 112, press <<W>> and get 1 points.
You see the image 121, press <<W>> and get 1 points.
You see the image 121, press <<W>> and get 1 points.
You see the image 211, press <<N>> and get 0 points.
You see the image 221, press <<N>> and get 1 points.
You see the image 212, press <<N>> and get 1 points.
You see the image 122, press <<W>> and get 0 points.
You see the image 112, press <<W>> and get 1 points.
You see the image 212, press <<N>> and get 1 points.
You see the image 112, press <<W>> and get 1 points.
You see the image 121, press <<N>> and get 0 points.
You see the image 122, press <<W>> and get 0 points.
You see the image 221, press <<N>> and get 1 points.
You see the image 211, press <<N>> and get 0 points.
You see the image 112, press <<W>> and get 1 points.
You see the image 122, press <<W>> and get 0 points.
You see the image 121, press <<N>> and get 0 points.
You see the image 221, press <<N>> and get 1 points.
You see the image 212, press <<W>> and get 0 points.
You see the image 211, p

Zoopermarket

Data source: [40]

Number of experiments: 3
Number of participants: 96
Number of choices: 34442

Example prompt:
You will have to repeatedly feed animals with fruits.
Each fruit contains two vitamins.
Every animal has a different preference for the vitamins.
The vitamin contents and the preferences are both given as vectors with two entries.
Your points are calculated as the dot product of the vitamin content with the preference of the current animal.
For example, let us assume that you have to feed the elephant who has a preference [-1 1].
Then, if you feed the elephant a fruit with vitamin content [-1 1], this would yield 2 points.
If you feed it a fruit with vitamins [1 0], this would yield -1 points.
You have to buy the fruits in a market, in which you can go left or right for two steps.
You can press I to go left, and V to go right.
Per round, you always collect two fruits.
There are eight animals in total and you have to feed one, two, or three of them in

each block.
In each block, there are twelve trials with different animals in random order.
After these twelve trials, there are three more in which you have to feed new animals.
The fruits in the market are rearranged after each block, meaning that you have to relearn the positions.
Your goal is to maximize the points obtained.

A new block starts. The locations of the fruits in the market got scrambled.
You have to feed the crocodile. It has the preference [1 1].
You press <<V>> and find the apple which has the vitamins [-1 -1]. You get -2 points.
You press <<V>> and find the orange which has the vitamins [0 1]. You get 1 points.
You have to feed the crocodile. It has the preference [1 1].
You press <<V>> and find the apple which has the vitamins [-1 -1]. You get -2 points.
You press <<I>> and find the blueberry which has the vitamins [-1 1]. You get 0 points.
You have to feed the crocodile. It has the preference [1 1].
You press <<I>> and find the strawberry which has the vitamins [1 1]. You get 2 points.
You press <<V>> and find the grapes which has the vitamins [1 -1]. You get 0 points.
You have to feed the crocodile. It has the preference [1 1].
You press <<I>> and find the strawberry which has the vitamins [1 1]. You get 2 points.
You press <<V>> and find the grapes which has the vitamins [1 -1]. You get 0 points.
You have to feed the kangaroo. It has the preference [-1 0].
You press <<V>> and find the apple which has the vitamins [-1 -1]. You get 1 points.
You press <<I>> and find the blueberry which has the vitamins [-1 1]. You get 1 points.
You have to feed the kangaroo. It has the preference [-1 0].
You press <<V>> and find the apple which has the vitamins [-1 -1]. You get 1 points.
You press <<I>> and find the blueberry which has the vitamins [-1 1]. You get 1 points.
You have to feed the crocodile. It has the preference [1 1].
You press <<I>> and find the strawberry which has the vitamins [1 1]. You get 2 points.
You press <<V>> and find the grapes which has the vitamins [1 -1]. You get 0 points.
You have to feed the kangaroo. It has the preference [-1 0].
You press <<V>> and find the apple which has the vitamins [-1 -1]. You get 1

points.
You press <<I>> and find the blueberry which has the vitamins [-1 1]. You get 1 points.
You have to feed the kangaroo. It has the preference [-1 0].
You press <<V>> and find the apple which has the vitamins [-1 -1]. You get 1 points.
You press <<I>> and find the blueberry which has the vitamins [-1 1]. You get 1 points.
You have to feed the kangaroo. It has the preference [-1 0].
You press <<V>> and find the apple which has the vitamins [-1 -1]. You get 1 points.
You press <<I>> and find the blueberry which has the vitamins [-1 1]. You get 1 points.
You have to feed the kangaroo. It has the preference [-1 0].
You press <<V>> and find the apple which has the vitamins [-1 -1]. You get 1 points.
You press <<I>> and find the blueberry which has the vitamins [-1 1]. You get 1 points.
You have to feed the crocodile. It has the preference [1 1].
You press <<V>> and find the apple which has the vitamins [-1 -1]. You get -2 points.
You press <<V>> and find the orange which has the vitamins [0 1]. You get 1 points.
You

choices13k

Data source: [5]

Number of experiments: 1
Number of participants: 13735
Number of choices: 1097375

Example prompt:
You will encounter a series of gambling problems where you have to select between two options.
You can select an option by pressing the corresponding key.
For some problems, you are told the points you received and missed out on after each selection, while for others this information is suppressed.
In cases where the probabilities are unknown, they sum up to one and remain constant within a problem.

Option L delivers 10.0 points with 80.0% chance, or -25.0 points with 20.0% chance.
Option B delivers 0.0 points with 20.0% chance, or 5.0 points with 80.0% chance.
You press <<B>>. You receive 5.0 points by selecting this option. You would have received 10.0 points had you chosen the other option.

You press <<B>>. You receive 5.0 points by selecting this option. You would have received -25.0 points had you chosen the other option.
You press <<B>>. You receive 5.0 points by selecting this option. You would have received 10.0 points had you chosen the other option.
You press <<B>>. You receive 5.0 points by selecting this option. You would have received -25.0 points had you chosen the other option.
You press <<B>>. You receive 0.0 points by selecting this option. You would have received -25.0 points had you chosen the other option.

Option L delivers either 30.0 points with 100.0% chance, or 30.0 points with 0.0% chance.
Option B delivers either 0.0 points with unknown chance, or 42.0 points with unknown chance.
You press <<L>>. You receive 30.0 points by selecting this option. You would have received 42.0 points had you chosen the other option.
You press <<L>>. You receive 30.0 points by selecting this option. You would have received 0.0 points had you chosen the other option.
You press <<L>>. You receive 30.0 points by selecting this option. You would have received 42.0 points had you chosen the other option.
You press <<L>>. You receive 30.0 points by selecting this option. You would have received 42.0 points had you chosen the other option.
You press <<B>>. You receive 42.0 points by selecting this option. You would have received 30.0 points had you chosen the other option.

Option L delivers 8.0 points with 100.0% chance, or 8.0 points with 0.0% chance.
Option B delivers 5.0 points with 95.0% chance, or 54.0 points with 5.0% chance.
You press <<B>>. You receive 5.0 points by selecting this option. You would have received 8.0 points had you chosen the other option.
You press <<B>>. You receive 5.0 points by selecting this option. You would have received 8.0 points had you chosen the other option.
You press <<B>>. You receive 5.0 points by selecting this option. You would have received 8.0 points had you chosen the other option.
You press <<B>>. You receive 5.0 points by selecting this option. You would have received 8.0 points had you chosen the other option.
You press <<B>>. You receive 5.0 points by selecting this option. You would have received 8.0 points had you chosen the other option.

Option L delivers 20.0 points with 100.0% chance, or 20.0 points with 0.0% chance.
Option B delivers 15.0 points with 99.0% chance, 47.5 points with 0.0078% chance, 48.5 points with 0.0547% chance, 49.5 points with 0.1641% chance, 50.5 points with 0.2734% chance, 51.5 points with 0.2734% chance, 52.5 points with 0.1641% chance, 53.5 points with 0.0547% chance, or 54.5 points with 0.0078% chance.
You press <<B>>. You receive 15.0 points by selecting this option. You would have received 20.0 points had you chosen the other option.
You press <<B>>. You receive 15.0 points by selecting this option. You would have

received 20.0 points had you chosen the other option.
You press <<B>>. You receive 15.0 points by selecting this option. You would have received 20.0 points had you chosen the other option.
You press <<B>>. You receive 15.0 points by selecting this option. You would have received 20.0 points had you chosen the other option.
You press <<B>>. You receive 15.0 points by selecting this option. You would have received 20.0 points had you chosen the other option.

Option L delivers 15.0 points with 5.0% chance, or 9.0 points with 95.0%

CPC18

Data source: [41]

Number of experiments: 1
Number of participants: 216
Number of choices: 162000

Example prompt:
You will encounter a series of gambling problems where you have to select between two options.
You can select an option by pressing the corresponding key.
You will encounter each problem 25 times.
In the first five encounters, you will not receive feedback.
In the remaining 20 encounters, you will receive feedback about the outcomes of both options.
In cases where the probabilities are stated to be unknown, they sum up to one and remain constant within a problem.

Option F delivers 3 points with 80.0% chance, 94 points with 1.25% chance, 95 points with 5.0% chance, 96 points with 7.5% chance, 97 points with 5.0% chance, 98 points with 1.25% chance.
Option X delivers -19 points with 50.0% chance, 59 points with 50.0% chance.
You press <<X>>.
You press <<X>>.
You press <<X>>.
You press <<X>>.
You press <<X>>.
You press <<X>> and gain 59 points. You would have gained 3 points had you chosen option F.
You press <<X>> and gain 59 points. You would have gained 98 points had you chosen option F.
You press <<X>> and gain 59 points. You would have gained 3 points had you chosen option F.
You press <<X>> and lose 19 points. You would have gained 95 points had you chosen option F.

You press <<X>> and lose 19 points. You would have gained 3 points had you chosen option F.
You press <<X>> and gain 59 points. You would have gained 3 points had you chosen option F.
You press <<X>> and lose 19 points. You would have gained 3 points had you chosen option F.
You press <<X>> and lose 19 points. You would have gained 3 points had you chosen option F.
You press <<F>> and gain 3 points. You would have lost 19 points had you chosen option X.
You press <<X>> and lose 19 points. You would have gained 3 points had you chosen option F.
You press <<X>> and lose 19 points. You would have gained 3 points had you chosen option F.
You press <<X>> and gain 59 points. You would have gained 3 points had you chosen option F.
You press <<X>> and gain 59 points. You would have gained 3 points had you chosen option F.
You press <<X>> and lose 19 points. You would have gained 3 points had you chosen option F.
You press <<X>> and lose 19 points. You would have gained 96 points had you chosen option F.
You press <<X>> and gain 59 points. You would have gained 3 points had you chosen option F.
You press <<X>> and lose 19 points. You would have gained 3 points had you chosen option F.
You press <<X>> and gain 59 points. You would have gained 98 points had you chosen option F.
You press <<X>> and gain 59 points. You would have gained 3 points had you chosen option F.
You press <<X>> and lose 19 points. You would have gained 3 points had you chosen option F.

Option F delivers -12 points with 95.0% chance, 47 points with 2.5% chance, 49 points with 1.25% chance, 53 points with 0.625% chance, 61 points with 0.3125% chance, 77 points with 0.15625% chance, 109 points with 0.078125% chance, 173 points with 0.078125% chance.
Option X delivers -9 points with 100.0% chance.
You press <<F>>.
You press <<F>>.
You press <<F>>.
You press <<F>>.
You press <<F>>.
You press <<F>> and lose 12 points. You would have lost 9 points had you chosen option X.

You press <<F>> and lose 12 points. You would have lost 9 points had you chosen option X.
You press <<F>> and lose 12 points. You would have lost 9 points had you chosen option X.
You press <<F>> and lose 12 points. You would have lost 9 points had you chosen option X.
You press <<F>> and lose 12 points. You would have lost 9 points had you chosen option X.
You press <<F>> and lose 12 points. You would have lost 9 points had you chosen option X.
You press <<F>> and lose 12 points. You would have lost 9 points had you chosen option X.
You press <<F>> and lose 12 points. You would have lost 9 points had you chosen option X.
You press <<F>> and gain 49 points. You would have lost 9 points had you chosen option X.
You press <<F>> and gain 53 points. You would have lost 9 points had you chosen option X.
You press <<F>> and lose 12 points. You would have lost 9 points had you chosen option X.
You press <<F>> and lose 12 points. You would

Episodic long-term memory

Data source: [42]

Number of experiments: 3
Number of participants: 132
Number of choices: 18649

Example prompt:
In this experiment, you will go through three cycles of three tasks.
Each cycle concerns itself with a list of 30 words for you to study.
In the first task of each cycle, you will go through the list.
You need to remember the words indicated by a red border.
For each word, please make a judgement on whether the object is larger or smaller than a football, and press the key "O" if the object is larger than a football, and the key "M" if it is smaller instead.
In the second task of each cycle, you will try to solve as many arithmetic equations as you can in one minute.
In the third task of each cycle, you will recall the words that you memorized in the first task of that cycle.

List 1, task 1:
You see the word "church", surrounded by a blue border. You press <<O>>. The word disappears but the blue border stays for another 3 seconds before disappearing.

You see the word ”robot”, surrounded by a blue border. You press <<O>>. The word disappears but the blue border stays for another 3 seconds before disappearing.
You see the word ”jewelry”, surrounded by a red border. You press <<M>>. The word disappears but the red border stays for another 3 seconds before disappearing.
You see the word ”skull”, surrounded by a blue border. You press <<O>>. The word disappears but the blue border stays for another 3 seconds before disappearing.
You see the word ”apple”, surrounded by a blue border. You press <<M>>. The word disappears but the blue border stays for another 3 seconds before disappearing.
You see the word ”garden”, surrounded by a blue border. You press <<O>>. The word disappears but the blue border stays for another 3 seconds before disappearing.
You see the word ”pipe”, surrounded by a red border. You press <<M>>. The word disappears but the red border stays for another 3 seconds before disappearing.
You see the word ”needle”, surrounded by a red border. You press <<M>>. The word disappears but the red border stays for another 3 seconds before disappearing.
You see the word ”circus”, surrounded by a red border. You press <<O>>. The word disappears but the red border stays for another 3 seconds before disappearing.
You see the word ”towel”, surrounded by a blue border. You press <<M>>. The word disappears but the blue border stays for another 3 seconds before disappearing.
You see the word ”rabbit”, surrounded by a blue border. You press <<M>>. The word disappears but the blue border stays for another 3 seconds before disappearing.
You see the word ”diamond”, surrounded by a red border. You press <<M>>. The word disappears but the red border stays for another 3 seconds before disappearing.
You see the word ”cocktail”, surrounded by a red border. You press <<M>>. The word disappears but the red border stays for another 3 seconds before disappearing.
You see the word ”satellite”, surrounded by a red border. You press <<O>>. The word disappears but the red border stays for another 3 seconds before disappearing.
You see the word ”sweater”, surrounded by a blue border. You press <<O>>. The word disappears but the blue border stays for another 3 seconds before disappearing.
You see the word ”planet”, surrounded by a red border. You press <<O>>. The word disappears but the red border stays for another 3 seconds before disappearing.
You see the word ”pizza”, surrounded by a blue border. You press <<O>>. The word disappears but the blue border stays for another 3 seconds before disappearing.
You see the word ”forest”, surrounded by a blue border. You press <<O>>. The word disappears but the blue border stays for another 3 seconds before disappearing.
You see the word ”carpet”, surrounded by a blue border. You press <<O>>. The word disappears but the blue border stays for another 3 seconds before disappearing.
You see the word ”highway”, surrounded by a red border. You press <<O>>. The word disappears but the red border stays for another 3 seconds before disappearing.
You see the word ”jeep”, surrounded by a blue border. You press <<O>>. The word disappears but the blue border stays for another 3 seconds before disappearing.
You see t

Intertemporal choice

Data source: [43]

Number of experiments: 1
Number of participants: 11937
Number of choices: 142236

Example prompt:
In the following you will be presented with multiple choices between two options G and C.
Please name which option you would prefer by pressing the corresponding key.

You have the choice between receiving 500$ immediately (press G) or receiving 550$ in one year (press C). You press <<G>>.
You have the choice between receiving 500$ immediately (press G) or receiving 600$ in one year (press C). You press <<C>>.
You have the choice between paying 500$ immediately (press G) or paying 550$ in one year (press C). You press <<G>>.
You have the choice between paying 500$ immediately (press G) or paying 510$ in one year (press C). You press <<C>>.
You have the choice between receiving 5000$ immediately (press G) or receiving 5500$ in one year (press C). You press <<G>>.
You have the choice between receiving 5000$ immediately (press G) or receiving 6000$ in one year (press C). You press <<C>>.
You have the choice between receiving 500$ in one year (press G) or receiving 600$ in two years (press C). You press <<C>>.
You have the choice between receiving 500$ immediately (press G) or receiving 700$ in two years (press C). You press <<C>>.
You have the choice between wait 500$ immediately (press G) or wait 600$ in one year (press C). You press <<C>>.
You have the choice between receive 500$ immediately (press G) or receive 600$ in one year (press C). You press <<C>>.

Horizon task

Data source: [44]

Number of experiments: 1
Number of participants: 78
Number of choices: 25336

Example prompt:
You are participating in multiple games involving two slot machines, labeled J and R.
The two slot machines are different across different games.
Each time you choose a slot machine, you get some points.
You choose a slot machine by pressing the corresponding key.
Each slot machine tends to pay out about the same amount of points on average.
Your goal is to choose the slot machines that will give you the most points across the experiment.

The first 4 trials in each game are instructed trials where you will be told which slot machine to choose.
After these instructed trials, you will have the freedom to choose for either 1 or 6 trials.

Game 1. There are 10 trials in this game.
You are instructed to press J and get 57 points.
You are instructed to press R and get 29 points.
You are instructed to press J and get 66 points.
You are instructed to press R and get 38 points.
You press <<R>> and get 45 points.
You press <<R>> and get 38 points.
You press <<R>> and get 49 points.
You press <<J>> and get 59 points.
You press <<R>> and get 28 points.
You press <<R>> and get 51 points.

Game 2. There are 10 trials in this game.
You are instructed to press J and get 76 points.
You are instructed to press J and get 89 points.
You are instructed to press R and get 61 points.
You are instructed to press J and get 74 points.
You press <<R>> and get 59 points.
You press <<J>> and get 72 points.
You press <<J>> and get 61 points.
You press <<R>> and get 70 points.
You press <<R>> and get 73 points.
You press <<J>> and get 65 points.

Game 3. There are 10 trials in this game.
You are instructed to press R and get 60 points.
You are instructed to press R and get 43 points.
You are instructed to press J and get 54 points.
You are instructed to press J and get 65 points.
You press <<R>> and get 56 points.
You press <<J>> and get 38 points.
You press <<R>> and get 56 points.
You press <<J>> and get 61 points.
You press <<R>> and get 59 points.
You press <<J>> and get 56 points.

Game 4. There are 5 trials in this game.
You are instructed to press J and get 36 points.
You are instructed to press J and get 43 points.
You are instructed to press R and get 57 points.

You are instructed to press R and get 49 points.
You press <<R>> and get 63 points.

Game 5. There are 5 trials in this game.
You are instructed to press R and get 44 points.
You are instructed to press J and get 45 points.
You are instructed to press R and get 29 points.
You are instructed to press J and get 38 points.
You press <<R>> and get 43 points.

Game 6. There are 5 trials in this game.
You are instructed to press R and get 74 points.
You are instructed to press R and get 70 points.
You are instructed to press R and get 61 points.
You are instructed to press J and get 77 points.
You press <<J>> and get 74 points.

Game 7. There are 5 trials in this game.
You are instructed to press J and get 36 points.
You are instructed to press R and get 50 points.
You are instructed to press J and get 49 points.
You are instructed to press J and get 34 points.
You press <<R>> and get 48 points.

Game 8. There are 10 trials in this game.
You are instructed to press J and get 54 points.
You are instructed to press R and get 64 points.
You are instructed to press R and get 63 points.
You are instructed to press R and get 52 points.
You press <<J>> and get 63 points.
You press <<J>> and get 65 points.
You press <<R>> and get 70 points.
You press <<J>> and get 69 points.
You press <<R>> and get 64 points.
You press <<J>> and get 64 points.

Game 9. There are 5 trials in this game.
You are instructed to press J and get 57 points.
You are instructed to press R and get 50 points.
You are instructed to press R and get 57 points.
You are instructed to press J and get 72 points.
You press <<R>> and get 47 points.

Game 10. There are 10 trials in this game.
You are instructed to press R and get 21 points.

You are instructed to press J and get 26 points.
You are instructed to press J and get 52 points.
You are instructed to press J and get 27 points.
You press <<R>> and get 21 points.
You press <<J>> a

Structured bandit

Data source: [45]

Number of experiments: 5
Number of participants: 534
Number of choices: 160200

Example prompt:
You will be playing a game for 30 rounds.
Each round contains 10 trials.
In each trial, you have to select one option that will generate a reward between 0 and 50 points.
You can choose between options 1, 2, 3, 4, 5, 6, 7 and 8 by pressing the corresponding key.
After each round the options reset and each option can produce different rewards in the following round.
Your goal is to maximize your reward.

You are playing round 1:
You press <<1>> and get 13.927462234 points.
You press <<2>> and get 36.7688570508 points.
You press <<1>> and get 14.2022179045 points.
You press <<1>> and get 14.255711791 points.
You press <<1>> and get 14.0630012349 points.
You press <<1>> and get 13.7662251776 points.
You press <<1>> and get 14.0950976416 points.
You press <<1>> and get 13.9059322374 points.
You press <<1>> and get 13.7876455405 points.
You press <<1>> and get 14.0791620504 points.

You are playing round 2:
You press <<1>> and get 7.9614376644 points.
You press <<1>> and get 8.0581019194 points.
You press <<1>> and get 7.8981838872 points.
You press <<1>> and get 7.6801851393 points.
You press <<1>> and get 7.8750440099 points.
You press <<1>> and get 7.8730616431 points.
You press <<1>> and get 7.9118344028 points.
You press <<1>> and get 8.134691905 points.

You press <<1>> and get 7.3146307967 points.
You press <<1>> and get 7.5832954876 points.

You are playing round 3:
You press <<1>> and get 45.5086514616 points.
You press <<1>> and get 45.4708060494 points.
You press <<1>> and get 45.9992623342 points.
You press <<1>> and get 45.2562607277 points.
You press <<1>> and get 45.4858045741 points.
You press <<1>> and get 45.5714527483 points.
You press <<1>> and get 45.6341629546 points.
You press <<1>> and get 45.0394158823 points.
You press <<1>> and get 45.4305272183 points.
You press <<1>> and get 45.5876540303 points.

You are playing round 4:
You press <<1>> and get 11.3846751956 points.
You press <<1>> and get 11.5363886375 points.
You press <<1>> and get 11.7231131775 points.
You press <<1>> and get 11.6522950289 points.
You press <<1>> and get 11.526071202 points.
You press <<1>> and get 11.8137491734 points.
You press <<1>> and get 11.3114379632 points.
You press <<1>> and get 11.9434174388 points.
You press <<1>> and get 11.2375196571 points.
You press <<1>> and get 11.7253908566 points.

You are playing round 5:
You press <<1>> and get 6.3035553498 points.
You press <<1>> and get 6.1091934289 points.
You press <<1>> and get 5.8593245302 points.
You press <<1>> and get 6.7472106803 points.
You press <<1>> and get 5.8960908181 points.
You press <<1>> and get 6.4056392625 points.
You press <<1>> and get 6.2178725578 points.
You press <<1>> and get 6.0009585684 points.
You press <<1>> and get 6.5478514159 points.
You press <<1>> and get 6.1982281156 points.

You are playing round 6:
You press <<1>> and get 7.6893330342 points.
You press <<1>> and get 7.3522351643 points.
You press <<1>> and get 7.5121866021 points.
You press <<1>> and get 7.7384186564 points.
You press <<1>> and get 6.8107475324 points.

You press <<4>> and get 24.8575884026 points.
You press <<2>> and get 13.8675683188 points.
You press <<1>> and get 7.8646759927 points.
You press <<4>> and get 24.9148267139 points.
You press <<2>> and get 13.7239940234 points.

You are playing round 7:
You press <<1>> and get 4.8637779497 points.
You press <<4>> and get 21.4211685718 points.
You press <<2>> and get 9.9283331067 points.
You press <<1>> and get 4.3283739798 points.
You press <<1>> and get 4.2486815414 points.
You press <<2>> and get 9.7358045234 points.
You press <<3>> and get 15.5522984698 points.
You press <<4>> and get 21.1157043351 points.
You press <<4>> and get 21.2293801786 points.
You press <<4>> and get 21.2346665196 points.

You are playing round 8:
You press <<5>> and get 21.2600225368 points.
You press <<6>> and get 14.9281922416 points.
You press <<6>> and get 15.1378822594 points.
You press <<6>> and get 15.0143768307 points.
You press <<8>> and get 2.1704472833 points.
You press <<7>> and get 8.4601420045 points.
You press <<5>> and get

Horizon task

Data source: [46]

Number of experiments: 1
Number of participants: 78
Number of choices: 43680

Example prompt:
You are participating in multiple games involving two slot machines, labeled F and N.
The two slot machines are different across different games.
Each time you choose a slot machine, you get some points.
You choose a slot machine by pressing the corresponding key.
Each slot machine tends to pay out about the same amount of points on average.
Your goal is to choose the slot machines that will give you the most points across the experiment.
The first 4 trials in each game are instructed trials where you will be told which slot machine to choose.

After these instructed trials, you will have the freedom to choose for either 1 or 6 trials.

Game 1. There are 5 trials in this game.
You are instructed to press F and get 63 points.
You are instructed to press N and get 60 points.
You are instructed to press N and get 64 points.
You are instructed to press N and get 74 points.
You press <<N>> and get 65 points.

Game 2. There are 10 trials in this game.
You are instructed to press N and get 36 points.
You are instructed to press N and get 41 points.
You are instructed to press F and get 40 points.
You are instructed to press F and get 33 points.
You press <<N>> and get 44 points.
You press <<N>> and get 34 points.
You press <<F>> and get 48 points.
You press <<F>> and get 50 points.
You press <<N>> and get 38 points.
You press <<F>> and get 35 points.

Game 3. There are 5 trials in this game.
You are instructed to press F and get 18 points.
You are instructed to press N and get 51 points.
You are instructed to press N and get 41 points.
You are instructed to press F and get 23 points.
You press <<N>> and get 42 points.

Game 4. There are 10 trials in this game.
You are instructed to press F and get 65 points.
You are instructed to press N and get 55 points.
You are instructed to press N and get 68 points.
You are instructed to press N and get 61 points.
You press <<F>> and get 54 points.
You press <<N>> and get 74 points.
You press <<N>> and get 71 points.
You press <<N>> and get 42 points.
You press <<F>> and get 63 points.
You press <<N>> and get 53 points.

Game 5. There are 10 trials in this game.
You are instructed to press N and get 18 points.
You are instructed to press F and get 53 points.
You are instructed to press F and get 50 points.

You are instructed to press N and get 19 points.
You press <<F>> and get 46 points.
You press <<F>> and get 44 points.
You press <<N>> and get 10 points.
You press <<F>> and get 42 points.
You press <<F>> and get 39 points.
You press <<F>> and get 44 points.

Game 6. There are 10 trials in this game.
You are instructed to press F and get 13 points.
You are instructed to press N and get 51 points.
You are instructed to press F and get 1 points.
You are instructed to press N and get 36 points.
You press <<N>> and get 38 points.
You press <<N>> and get 36 points.
You press <<N>> and get 52 points.
You press <<N>> and get 36 points.
You press <<N>> and get 27 points.
You press <<F>> and get 12 points.

Game 7. There are 5 trials in this game.
You are instructed to press F and get 65 points.
You are instructed to press N and get 50 points.
You are instructed to press F and get 74 points.
You are instructed to press F and get 55 points.
You press <<N>> and get 72 points.

Game 8. There are 10 trials in this game.
You are instructed to press N and get 29 points.
You are instructed to press F and get 42 points.
You are instructed to press F and get 40 points.
You are instructed to press F and get 42 points.
You press <<F>> and get 53 points.
You press <<F>> and get 47 points.
You press <<N>> and get 40 points.
You press <<N>> and get 41 points.
You press <<F>> and get 38 points.
You press <<F>> and get 51 points.

Game 9. There are 5 trials in this game.
You are instructed to press N and get 60 points.
You are instructed to press N and get 66 points.
You are instructed to press F and get 61 points.
You are instructed to press F and get 65 points.
You press <<N>> and get 54 points.

Game 10. There are 10 trials in this game.
You are instructed to press F and get 44 points.
You are instructed to pr

Weather prediction task

Data source: [47]

Number of experiments: 1
Number of participants: 23
Number of choices: 4600

Example prompt:
You will be playing a game in which you pretend to be a weather forecaster.
In each trial, you will see between one and three tarot cards.
Your task is to decide if the combination of cards presented predicts rainy weather (by pressing E) or fine weather (by pressing J).

You are seeing the following: card 2, card 4. You press <<E>>. You are correct, the weather is indeed rainy.
You are seeing the following: card 1, card 2. You press <<J>>. You are correct, the weather is indeed fine.
You are seeing the following: card 3, card 4. You press <<J>>. You are wrong, the weather is rainy.
You are seeing the following: card 2, card 3. You press <<J>>. You are correct, the weather is indeed fine.
You are seeing the following: card 1. You press <<E>>. You are wrong, the weather is fine.
You are seeing the following: card 1, card 2, card 3. You press <<E>>. You are wrong, the weather is fine.
You are seeing the following: card 1, card 2, card 4. You press <<J>>. You are wrong, the weather is rainy.
You are seeing the following: card 1, card 2, card 4. You press <<E>>. You are correct, the weather is indeed rainy.
You are seeing the following: card 3, card 4. You press <<E>>. You are correct, the weather is indeed rainy.
You are seeing the following: card 3, card 4. You press <<E>>. You are correct, the weather is indeed rainy.
You are seeing the following: card 1. You press <<J>>. You are correct, the weather is indeed fine.
You are seeing the following: card 1, card 4. You press <<J>>. You are correct, the weather is indeed fine.
You are seeing the following: card 4. You press <<E>>. You are wrong, the weather is fine.
You are seeing the following: card 3, card 4. You press <<E>>. You are correct, the weather is indeed rainy.

You are seeing the following: card 1, card 2. You press <<J>>. You are correct, the weather is indeed fine.
You are seeing the following: card 3, card 4. You press <<E>>. You are correct, the weather is indeed rainy.
You are seeing the following: card 2. You press <<J>>. You are correct, the weather is indeed fine.
You are seeing the following: card 1, card 4. You press <<J>>. You are wrong, the weather is rainy.
You are seeing the following: card 1, card 4. You press <<J>>. You are correct, the weather is indeed fine.
You are seeing the following: card 1. You press <<J>>. You are correct, the weather is indeed fine.
You are seeing the following: card 1, card 3, card 4. You press <<J>>. You are correct, the weather is indeed fine.
You are seeing the following: card 2. You press <<J>>. You are correct, the weather is indeed fine.
You are seeing the following: card 1, card 2. You press <<J>>. You are correct, the weather is indeed fine.
You are seeing the following: card 2, card 3, card 4. You press <<E>>. You are correct, the weather is indeed rainy.
You are seeing the following: card 3. You press <<J>>. You are correct, the weather is indeed fine.
You are seeing the following: card 1, card 2, card 3. You press <<J>>. You are wrong, the weather is rainy.
You are seeing the following: card 3, card 4. You press <<E>>. You are correct, the weather is indeed rainy.
You are seeing the following: card 1, card 3. You press <<J>>. You are correct, the weather is indeed fine.
You are seeing the following: card 1, card 4. You press <<J>>. You are wrong, the weather is rainy.
You are seeing the following: card 2, card 3, card 4. You press <<E>>. You are wrong, the weather is fine.
You are seeing the following: card 2, card 4. You press <<E>>. You are correct, the weather is indeed rainy.
You are seeing the following: card 4. You press <<J>>. You are wrong, the weather is rainy.
You are seeing the following: card 1, card 2. You press <<J>>. You are correct, the weather is indeed fine.
You are seeing the following: card 1, card 3, card 4. You press <<E>>. You are correct, the weather is indeed rainy.
You are seeing the following: card 4. You press <<J>>. You are wrong, the weather is rainy.
You are seeing the following: card 1, card 2. You press <<J>>. You are correct, the weather is indeed fine.
You are seeing the follow

Iowa gambling task

Data source: [48]

Number of experiments: 9
Number of participants: 511
Number of choices: 55435

Example prompt:
You see in front of you four decks of cards labeled H, V, J, and D.
You get a loan of 2000$ of play money.
You have to select one card at a time, from any of the four decks, for 100 trials.
You select a card from a deck by pressing the corresponding key.
After turning a card, you win some money, the amount varies with the deck.
You sometimes also have to pay a penalty, which also varies with the deck.
Your goal is to maximize profit on the loan of the play money.

You press <<H>>. You win 100.0$ and lose 200.0$.
You press <<H>>. You win 100.0$ and lose 150.0$.
You press <<V>>. You win 100.0$ and lose 0.0$.
You press <<D>>. You win 50.0$ and lose 250.0$.
You press <<J>>. You win 50.0$ and lose 0.0$.
You press <<V>>. You win 100.0$ and lose 0.0$.
You press <<H>>. You win 100.0$ and lose 0.0$.
You press <<V>>. You win 100.0$ and lose 0.0$.
You press <<D>>. You win 50.0$ and lose 0.0$.
You press <<V>>. You win 100.0$ and lose 0.0$.
You press <<J>>. You win 50.0$ and lose 0.0$.
You press <<J>>. You win 50.0$ and lose 0.0$.
You press <<H>>. You win 100.0$ and lose 300.0$.
You press <<V>>. You win 100.0$ and lose 0.0$.
You press <<V>>. You win 100.0$ and lose 1250.0$.
You press <<D>>. You win 50.0$ and lose 0.0$.
You press <<J>>. You win 50.0$ and lose 50.0$.
You press <<H>>. You win 100.0$ and lose 0.0$.
You press <<V>>. You win 100.0$ and lose 0.0$.
You press <<D>>. You win 50.0$ and lose 0.0$.
You press <<J>>. You win 50.0$ and lose 0.0$.
You press <<D>>. You win 50.0$ and lose 0.0$.
You press <<V>>. You win 100.0$ and lose 0.0$.
You press <<H>>. You win 100.0$ and lose 0.0$.
You press <<D>>. You win 50.0$ and lose 0.0$.
You press <<H>>. You win 100.0$ and lose 0.0$.
You press <<J>>. You win 50.0$ and lose 50.0$.
You press <<D>>. You win 50.0$ and lose 0.0$.
You press <<V>>. You win 100.0$ and lose 0.0$.

You press <<H>>. You win 100.0$ and lose 0.0$.
You press <<V>>. You win 100.0$ and lose 0.0$.
You press <<D>>. You win 50.0$ and lose 0.0$.
You press <<H>>. You win 100.0$ and lose 250.0$.
You press <<J>>. You win 50.0$ and lose 50.0$.
You press <<D>>. You win 50.0$ and lose 0.0$.
You press <<V>>. You win 100.0$ and lose 0.0$.
You press <<H>>. You win 100.0$ and lose 350.0$.
You press <<V>>. You win 100.0$ and lose 0.0$.
You press <<D>>. You win 50.0$ and lose 0.0$.
You press <<J>>. You win 50.0$ and lose 0.0$.
You press <<H>>. You win 100.0$ and lose 300.0$.
You press <<V>>. You win 100.0$ and lose 0.0$.
You press <<D>>. You win 50.0$ and lose 0.0$.
You press <<V>>. You win 100.0$ and lose 1250.0$.
You press <<H>>. You win 100.0$ and lose 0.0$.
You press <<V>>. You win 100.0$ and lose 0.0$.
You press <<D>>. You win 50.0$ and lose 0.0$.
You press <<J>>. You win 50.0$ and lose 50.0$.
You press <<H>>. You win 100.0$ and lose 0.0$.
You press <<V>>. You win 100.0$ and lose 0.0$.
You press <<D>>. You win 50.0$ and lose 0.0$.
You press <<J>>. You win 50.0$ and lose 50.0$.
You press <<H>>. You win 100.0$ and lose 0.0$.
You press <<V>>. You win 100.0$ and lose 0.0$.
You press <<D>>. You win 50.0$ and lose 250.0$.
You press <<J>>. You win 50.0$ and lose 0.0$.
You press <<D>>. You win 50.0$ and lose 0.0$.
You press <<V>>. You win 100.0$ and lose 0.0$.
You press <<H>>. You win 100.0$ and lose 150.0$.
You press <<H>>. You win 100.0$ and lose 200.0$.
You press <<H>>. You win 100.0$ and lose 350.0$.
You press <<V>>. You win 100.0$ and lose 0.0$.
You press <<V>>. You win 100.0$ and lose 0.0$.
You press <<V>>. You win 100.0$ and lose 0.0$.
You press <<V>>. You win 100.0$ and lose 0.0$.
You press <<V>>. You win 100.0$ and lose 0.0$.
You press <<V>>. You win 100.0$ and lose 0.0$.
You press <<D>>. You win 50.0$ and lose 0.0$.
You press <<V>>. You win 100.0$ and lose 0.0$.
You press <<V>>. You win 100.0$ and lose 1250.0$.
You press <<J>>. You win 50.0$ and lose 0.0$.
You press <<J>>. You win 50.0$ and lose 50.0$.
You press <<D>>. You win 50.0$ and lose 0.0$.
You press <<V>>. You win 100.0$ and lose 0.0$.

You press <<V>>. You win 100.0$ and lose 0.0$.
You press <<V>>. You win 100.0$ and lose 0.0$.
You press <<D>>. You win 50.0$ and lose 0.0$.
Yo

Virtual subway network

Data source: [49]

Number of experiments: 4
Number of participants: 789
Number of choices: 227923

Example prompt:
Imagine that you are a tourist and you have to navigate the subway network in an unfamiliar town.
In each round, you will have to navigate from a starting station to a goal station.
Please try to plan your trip as quickly as possible.
During your trip, you will see the name of the current station and its neighboring stations in all four directions.
If there is no neighboring station in a particular direction, there will be a circle instead of a station name.
You can go north by pressing G, west by pressing B, south by pressing V, and east by pressing C.
When you reach the goal station, press Z to end the round and start the next round.

The new starting station is 1 and the goal station is 3.
Your station: 1. Neighboring stations: circle on the north, 2 on the east, circle on the south, and 9 on the west. You press <<B>>.
Your station: 9. Neighboring stations: circle on the north, 1 on the east, 8 on the south, and circle on the west. You press <<V>>.
Your station: 8. Neighboring stations: 9 on the north, circle on the east, 7 on the south, and circle on the west. You press <<V>>.
Your station: 7. Neighboring stations: 8 on the north, 6 on the east, circle on the south, and circle on the west. You press <<C>>.
Your station: 6. Neighboring stations: circle on the north, 5 on the east, circle on the south, and 7 on the west. You press <<C>>.
Your station: 5. Neighboring stations: 4 on the north, circle on the east, circle on the south, and 6 on the west. You press <<G>>.
Your station: 4. Neighboring stations: 3 on the north, circle on the east, 5 on the south, and circle on the west. You press <<G>>.
Your station: 3. Neighboring stations: 2 on the north, circle on the east, 4 on the south, and circle on the west. You press <<Z>>.
You are successful.

The new starting station is 1 and the goal station is 3.

Your station: 1. Neighboring stations: circle on the north, 2 on the east, circle on the south, and 9 on the west. You press <<B>>.
Your station: 9. Neighboring stations: circle on the north, 1 on the east, 8 on the south, and circle on the west. You press <<V>>.
Your station: 8. Neighboring stations: 9 on the north, circle on the east, 7 on the south, and circle on the west. You press <<V>>.
Your station: 7. Neighboring stations: 8 on the north, 6 on the east, circle on the south, and circle on the west. You press <<C>>.
Your station: 6. Neighboring stations: circle on the north, 5 on the east, circle on the south, and 7 on the west. You press <<C>>.
Your station: 5. Neighboring stations: 4 on the north, circle on the east, circle on the south, and 6 on the west. You press <<G>>.
Your station: 4. Neighboring stations: 3 on the north, circle on the east, 5 on the south, and circle on the west. You press <<G>>.
Your station: 3. Neighboring stations: 2 on the north, circle on the east, 4 on the south, and circle on the west. You press <<Z>>.
You are successful.

The new starting station is 4 and the goal station is 6.
Your station: 4. Neighboring stations: 3 on the north, circle on the east, 5 on the south, and circle on the west. You press <<V>>.
Your station: 5. Neighboring stations: 4 on the north, circle on the east, circle on the south, and 6 on the west. You press <<B>>.
Your station: 6. Neighboring stations: circle on the north, 5 on the east, circle on the south, and 7 on the west. You press <<Z>>.
You are successful.

The new starting station is 1 and the goal station is 7.
Your station: 1. Neighboring stations: circle on the north, 2 on the east, circle on the south, and 9 on the west. You press <<B>>.
Your station: 9. Neighboring stations: circle on the north, 1 on the east, 8 on the south, and circle on the west. You press <<V>>.
Your station: 8. Neighboring stations: 9 on the north, circle on the east, 7 on the south, and circle on the west. You press <<V>>.
Your station: 7. Neighboring stations: 8 on the north, 6 on the east, circle on the south, and circle on the west. You press <<Z>>.
You are successful.

The new starting station is 9 and the goal station is 7.
Your station: 9. Neighboring stations: circle

Multi-task reinforcement learning

Data source: [14]

Number of experiments: 2

Number of participants: 380
Number of choices: 76760

Example prompt:
You will explore a castle, walking from room to room.
In each room, you will find different amounts of resources: wood, stone, and iron.
In each room, there are three doors that lead to different rooms.
The doors are labeled I, P, and G.
You have to choose the right doors to find the most valuable resources.
You choose a door by pressing the corresponding key.
At the beginning of each round, you will be shown how valuable the resources are.
These values are given as market prices for wood, stone, and iron.
Multiplying the prices with the amounts of resources and adding them up yields a reward.
You want to maximize the cumulative reward.
After every round, you will start in room 0 again and see the new market prices.

The current market prices are 1 for wood, -1 for stone, and 0 for iron.
You are in room 0. You press <<P>> and you find 0 wood, 0 stone, and 0 iron. You get 0 points.
You are in room 2. You press <<P>> and you find 100 wood, 100 stone, and 0 iron. You get 0 points.
The current market prices are -1 for wood, 1 for stone, and 0 for iron.
You are in room 0. You press <<I>> and you find 0 wood, 0 stone, and 0 iron. You get 0 points.
You are in room 1. You press <<G>> and you find 70 wood, 70 stone, and 70 iron. You get 0 points.
The current market prices are -1 for wood, 1 for stone, and 0 for iron.
You are in room 0. You press <<P>> and you find 0 wood, 0 stone, and 0 iron. You get 0 points.
You are in room 2. You press <<G>> and you find 0 wood, 90 stone, and 0 iron. You get 90 points.
The current market prices are -2 for wood, 1 for stone, and 0 for iron.
You are in room 0. You press <<P>> and you find 0 wood, 0 stone, and 0 iron. You get 0 points.
You are in room 2. You press <<G>> and you find 0 wood, 90 stone, and 0 iron. You get 90 points.
The current market prices are 1 for wood, -2 for stone, and 0 for iron.
You are in room 0. You press <<I>> and you find 0 wood, 0 stone, and 0 iron. You get 0 points.
You are in room 1. You press <<G>> and you find 70 wood, 70 stone, and 70 iron. You get -70 points.
The current market prices are -1 for wood, 1 for stone, and 0 for iron.
You are in room 0. You press <<P>> and you find 0 wood, 0 stone, and 0 iron. You get 0 points.

You are in room 2. You press <<G>> and you find 0 wood, 90 stone, and 0 iron. You get 90 points.
The current market prices are 1 for wood, -2 for stone, and 0 for iron.
You are in room 0. You press <<P>> and you find 0 wood, 0 stone, and 0 iron. You get 0 points.
You are in room 2. You press <<G>> and you find 0 wood, 90 stone, and 0 iron. You get -180 points.
The current market prices are 1 for wood, -1 for stone, and 0 for iron.
You are in room 0. You press <<G>> and you find 0 wood, 0 stone, and 0 iron. You get 0 points.
You are in room 3. You press <<P>> and you find 0 wood, 100 stone, and 60 iron. You get -100 points.
The current market prices are 1 for wood, -2 for stone, and 0 for iron.
You are in room 0. You press <<I>> and you find 0 wood, 0 stone, and 0 iron. You get 0 points.
You are in room 1. You press <<G>> and you find 70 wood, 70 stone, and 70 iron. You get -70 points.
The current market prices are -1 for wood, 1 for stone, and 0 for iron.
You are in room 0. You press <<P>> and you find 0 wood, 0 stone, and 0 iron. You get 0 points.
You are in room 2. You press <<G>> and you find 0 wood, 90 stone, and 0 iron. You get 90 points.
The current market prices are 1 for wood, -2 for stone, and 0 for iron.
You are in room 0. You press <<I>> and you find 0 wood, 0 stone, and 0 iron. You get 0 points.
You are in room 1. You press <<G>> and you find 70 wood, 70 stone, and 70 iron. You get -70 points.
The current market prices are -2 for wood, 1 for stone, and 0 for iron.
You are in room 0. You press <<P>> and you find 0 wood, 0 stone, and 0 iron. You get 0 points.
You are in room 2. You press <<G>> and you find 0 wood, 90 stone, and 0 iron. You get 90 points.
The current market prices are 1 for wood, -2 for stone, and 0 for iron.
You are in room 0. You press <<I>> and you find 0 wood, 0 stone, and 0 iron. You get 0 points.
You are in room

Horizon task

Data source: [50]

Number of experiments: 1
Number of participants: 36
Number of choices: 15290

Example prompt:

You are participating in multiple games involving two slot machines, labeled M and U.
The two slot machines are different across different games.
Each time you choose a slot machine, you get some points.
You choose a slot machine by pressing the corresponding key.
Each slot machine tends to pay out about the same amount of points on average.
Your goal is to choose the slot machines that will give you the most points across the experiment.
The first 4 trials in each game are instructed trials where you will be told which slot machine to choose.
After these instructed trials, you will have the freedom to choose for either 1 or 6 trials.

Game 1. There are 10 trials in this game.
You are instructed to press U and get 45 points.
You are instructed to press M and get 25 points.
You are instructed to press U and get 38 points.
You are instructed to press M and get 12 points.
You press <<U>> and get 38 points.
You press <<U>> and get 42 points.
You press <<U>> and get 44 points.
You press <<U>> and get 35 points.
You press <<U>> and get 42 points.
You press <<U>> and get 45 points.

Game 2. There are 10 trials in this game.
You are instructed to press U and get 71 points.
You are instructed to press M and get 35 points.
You are instructed to press U and get 71 points.
You are instructed to press M and get 26 points.
You press <<U>> and get 61 points.
You press <<U>> and get 55 points.
You press <<U>> and get 61 points.
You press <<U>> and get 61 points.
You press <<U>> and get 67 points.
You press <<U>> and get 69 points.

Game 3. There are 5 trials in this game.
You are instructed to press U and get 50 points.
You are instructed to press M and get 43 points.
You are instructed to press M and get 46 points.
You are instructed to press U and get 45 points.
You press <<U>> and get 61 points.

Game 4. There are 10 trials in this game.

You are instructed to press U and get 52 points.
You are instructed to press M and get 51 points.
You are instructed to press U and get 57 points.
You are instructed to press M and get 54 points.
You press <<U>> and get 67 points.
You press <<U>> and get 58 points.
You press <<U>> and get 68 points.
You press <<U>> and get 57 points.
You press <<U>> and get 68 points.
You press <<U>> and get 65 points.

Game 5. There are 10 trials in this game.
You are instructed to press U and get 32 points.
You are instructed to press M and get 32 points.
You are instructed to press M and get 38 points.
You are instructed to press U and get 33 points.
You press <<M>> and get 37 points.
You press <<M>> and get 48 points.
You press <<M>> and get 38 points.
You press <<M>> and get 49 points.
You press <<M>> and get 36 points.
You press <<M>> and get 48 points.

Game 6. There are 5 trials in this game.
You are instructed to press U and get 74 points.
You are instructed to press M and get 51 points.
You are instructed to press M and get 72 points.
You are instructed to press M and get 76 points.
You press <<U>> and get 57 points.

Game 7. There are 10 trials in this game.
You are instructed to press U and get 40 points.
You are instructed to press U and get 34 points.
You are instructed to press M and get 31 points.
You are instructed to press M and get 37 points.
You press <<U>> and get 41 points.
You press <<U>> and get 43 points.
You press <<U>> and get 47 points.
You press <<U>> and get 41 points.
You press <<U>> and get 44 points.
You press <<U>> and get 45 points.

Game 8. There are 5 trials in this game.
You are instructed to press U and get 56 points.
You are instructed to press M and get 59 points.

You are instructed to press M and get 48 points.
You are instructed to press M and get 48 points.
You press <<U>> and get 71 points.

Game 9. There are 5 trials in this game.
You are instructed to press U and get 46 points.
You are instructed to press M and get 46 points.
You are instructed to press U and get 47 points.
You are instructed to press U and get 56 points.
You press <<U>> and get 47 points.

Game 10. There are 10 trials in this game.
You are instructed to press M and get 49 points.
You are instructed to p

Horizon task

Data source: [8] and unpublished data from the authors

Number of experiments: 5
Number of participants: 221
Number of choices: 138875

Example prompt:
You are participating in multiple games involving two slot machines, labeled C and A.
The two slot machines are different across different games.
Each time you choose a slot machine, you get some points.
You choose a slot machine by pressing the corresponding key.
Each slot machine tends to pay out about the same amount of points on average.
Your goal is to choose the slot machines that will give you the most points across the experiment.
The first 4 trials in each game are instructed trials where you will be told which slot machine to choose.
After these instructed trials, you will have the freedom to choose for either 1 or 6 trials.

Game 1. There are 5 trials in this game.
You are instructed to press A and get 66 points.
You are instructed to press A and get 80 points.
You are instructed to press C and get 29 points.
You are instructed to press A and get 75 points.
You press <<A>> and get 81 points.

Game 2. There are 10 trials in this game.
You are instructed to press A and get 69 points.

You are instructed to press A and get 50 points.
You are instructed to press C and get 51 points.
You are instructed to press A and get 64 points.
You press <<C>> and get 42 points.
You press <<A>> and get 54 points.
You press <<A>> and get 64 points.
You press <<A>> and get 64 points.
You press <<A>> and get 57 points.
You press <<C>> and get 55 points.

Game 3. There are 10 trials in this game.
You are instructed to press A and get 31 points.
You are instructed to press C and get 43 points.
You are instructed to press A and get 26 points.
You are instructed to press C and get 36 points.
You press <<C>> and get 26 points.
You press <<C>> and get 41 points.
You press <<C>> and get 44 points.
You press <<C>> and get 44 points.
You press <<C>> and get 43 points.
You press <<C>> and get 53 points.

Game 4. There are 10 trials in this game.
You are instructed to press C and get 65 points.
You are instructed to press A and get 77 points.
You are instructed to press A and get 52 points.
You are instructed to press C and get 73 points.
You press <<C>> and get 61 points.
You press <<C>> and get 81 points.
You press <<C>> and get 70 points.
You press <<C>> and get 67 points.
You press <<A>> and get 62 points.
You press <<C>> and get 68 points.

Game 5. There are 10 trials in this game.
You are instructed to press A and get 70 points.
You are instructed to press C and get 19 points.
You are instructed to press A and get 43 points.
You are instructed to press C and get 41 points.
You press <<A>> and get 53 points.
You press <<C>> and get 19 points.
You press <<A>> and get 61 points.
You press <<A>> and get 68 points.
You press <<A>> and get 62 points.
You press <<C>> and get 46 points.

Game 6. There are 10 trials in this game.
You are instructed to press C and get 63 points.
You are instructed to press A and get 44 points.
You are instructed to press C and get 49 points.
You are instructed to press C and get 47 points.
You press <<A>> and get 52 points.
You press <<A>> and get 52 points.
You press <<C>> and get 55 points.
You press <<A>> and get 51 points.
You press <<A>> and get 34 points.
You press <<C>> and get 56 points.

Game 7. There are 5 trials in this game.
You are instructed to press C and get 61 points.
You are instructed to press A and get 44 points.
You are instructed to press A and get 41 points.
You are instructed to press A and get 47 points.
You press <<A>> and get 29 points.

Game 8. There are 5 trials in this game.
You are instructed to press C and get 51 points.
You are instructed to press A and get 76 points.
You are instructed to press C and get 54 points.
You are instructed to press A and get 84 points.
You press <<C>> and get 58 points.

Game 9. There are 10 trials in this game.
You are instructed to press C and get 54 points.
You are instructed to press A and get 14 points.
You are instructed to press A and get 15 points.
You are instructed to press C and get 46 points.
You press <<A>> and get 20 points.
You press <<C>> and get 44 points.
You press <<A>> and get 16 points.
You press <<C>> and get 46 points.
You press

Aversive learning

Data source: [51]

Number of experiments: 1
Number of participants: 57
Number of choices: 18240

Example prompt:

You are going to predict the probability of electric shocks associated with two visual stimuli.
First, you will have to indicate the probability that a stimulus predicts a shock at the current moment in time on a rating bar (between 0 and 100 percent).
After that, the outcome for each stimulus will be presented visually.
An upcoming shock will be indicated by a square over the stimulus, while a no-shock outcome will be indicated by a circle.
Finally, shocks will be delivered after you have learned about the outcome visually.
If both stimuli indicate a shock, they will be presented one after the other in random order.
The shock probability fluctuates over time such that one stimulus has a stable probability while the other varies.

Stimulus J and K are shown on the screen. You predict that the shock probability for stimulus J is <<50.0>>% and the shock probability for stimulus K is <<50.0>>%. After that, a circle is shown over stimulus J, and a square is shown over stimulus K. Finally, a shock is delivered for stimulus K.
Stimulus J and K are shown on the screen. You predict that the shock probability for stimulus J is <<50.0>>% and the shock probability for stimulus K is <<59.33>>%. After that, a square is shown over stimulus J, and a circle is shown over stimulus K. Finally, a shock is delivered for stimulus J.
Stimulus J and K are shown on the screen. You predict that the shock probability for stimulus J is <<55.72>>% and the shock probability for stimulus K is <<59.33>>%. After that, a circle is shown over stimulus J, and a circle is shown over stimulus K. Finally, no shocks are delivered.
Stimulus J and K are shown on the screen. You predict that the shock probability for stimulus J is <<55.72>>% and the shock probability for stimulus K is <<59.33>>%. After that, a circle is shown over stimulus J, and a circle is shown over stimulus K. Finally, no shocks are delivered.
Stimulus J and K are shown on the screen. You predict that the shock probability for stimulus J is <<55.72>>% and the shock probability for stimulus K is <<59.33>>%. After that, a circle is shown over stimulus J, and a circle is shown over stimulus K. Finally, no shocks are delivered.
Stimulus J and K are shown on the screen. You predict that the shock probability for stimulus J is <<55.72>>% and the shock probability for stimulus K is <<59.33>>%. After that, a circle is shown over stimulus J, and a square is shown over stimulus K. Finally, a shock is delivered for stimulus K.
Stimulus J and K are shown on the screen. You predict that the shock probability for stimulus J is <<55.72>>% and the shock probability for stimulus K is <<62.42>>%. After that, a circle is shown over stimulus J, and a circle is shown over stimulus K. Finally, no shocks are delivered.
Stimulus J and K are shown on the screen. You predict that the shock probability for stimulus J is <<55.72>>% and the shock probability for stimulus K is <<62.42>>%. After that, a circle is shown over stimulus J, and a circle is shown over stimulus K. Finally, no shocks are delivered.

Stimulus J and K are shown on the screen. You predict that the shock probability for stimulus J is <<55.72>>% and the shock probability for stimulus K is <<62.42>>%. After that, a square is shown over stimulus J, and a square is shown over stimulus K. Finally, shocks are delivered for both stimulus J and stimulus K.
Stimulus J and K are shown on the screen. You predict that the shock probability for stimulus J is <<58.2>>% and the shock probability for stimulus K is <<65.0>>%. After that, a circle is shown over stimulus J, and a square is shown over stimulus K. Finally, a shock is delivered for stimulus K.
Stimulus J and K are shown on the screen. You predict that the shock probability for stimulus J is <<58.2>>% and the shock probability for stimulus K is <<67.48>>%. After that, a circle is shown over stimulus J, and a circle is shown over stimulus K. Finally, no shocks are delivered.
Stimulus J and K are shown on the screen. You predict that the shock probability for stimulus J is <<58.2>>% and the shock probability

Spatially correlated multi-armed bandit

Data source: [15]

Number of experiments: 1
Number of participants: 78
Number of choices: 9360

Example prompt:
You will be presented with a series of 16 different environments to explore.
In each trial, you can select an option between numbers 1 and 30 by pressing the corresponding key.
By selecting any of these options, you will earn points associated with each unique option.
Imagine these options 1 through 30 as lying next to each other in an ordered line; options closer to each other tend to have similar rewards as rewards tend to cluster together.
For each environment, you will be able to make either 5 or 10 choices.
When you made all your choices in a given environment, you will start making choices in the next unexplored environment.
The rewards underlying the different options are different in each environment so you will learn them anew for each environment.
Each environment starts with the value of a single option revealed.
When you choose the number corresponding to a different option, you will be told the value of that option and receive those points.
Previously revealed options, including the starting option, can also be reselected, although there may be small changes in the point value.
It is your task to gain as many points as possible across all 16 environments.

Environment number 1:
The value of option 4 is 76. You have 5 choices to make in this environment.

You press <<5>> and receive 61 points.
You press <<3>> and receive 64 points.
You press <<6>> and receive 42 points.
You press <<2>> and receive 49 points.
You press <<11>> and receive 22 points.

Environment number 2:
The value of option 2 is 65. You have 10 choices to make in this environment.
You press <<3>> and receive 68 points.
You press <<1>> and receive 40 points.
You press <<9>> and receive 10 points.
You press <<17>> and receive 51 points.
You press <<18>> and receive 35 points.
You press <<16>> and receive 54 points.
You press <<14>> and receive 48 points.
You press <<23>> and receive 64 points.
You press <<22>> and receive 53 points.
You press <<23>> and receive 65 points.

Environment number 3:
The value of option 22 is 37. You have 5 choices to make in this environment.
You press <<13>> and receive 30 points.
You press <<7>> and receive 67 points.
You press <<8>> and receive 77 points.
You press <<6>> and receive 32 points.
You press <<9>> and receive 57 points.

Environment number 4:
The value of option 16 is 34. You have 10 choices to make in this environment.
You press <<5>> and receive 77 points.
You press <<6>> and receive 53 points.
You press <<4>> and receive 79 points.
You press <<3>> and receive 41 points.
You press <<22>> and receive 25 points.
You press <<29>> and receive 41 points.
You press <<26>> and receive 46 points.
You press <<12>> and receive 64 points.
You press <<11>> and receive 41 points.
You press <<13>> and receive 55 points.

Environment number 5:
The value of option 19 is 26. You have 5 choices to make in this environment.
You press <<8>> and receive 42 points.
You press <<3>> and receive 44 points.
You press <<24>> and receive 37 points.

You press <<29>> and receive 35 points.
You press <<15>> and receive 48 points.

Environment number 6:
The value of option 16 is 44. You have 10 choices to make in this environment.
You press <<9>> and receive 32 points.
You press <<4>> and receive 43 points.
You press <<21>> and receive 26 points.
You press <<26>> and receive 5 points.
You press <<1>> and receive 13 points.
You press <<6>> and receive 9 points.
You press <<12>> and receive 27 points.
You press <<18>> and receive 36 points.
You press <<23>> and receive 23 points.
You press <<17>> and receive 21 points.

Environment number 7:
The value of option 2 is 36. You have 5 choices to make in this environment.
You press <<9>> and receive 49 points.
You press <<16>> and receive 72 points.
You press <<17>> and receive 59 points.
You press <<15>> and receive 49 points.
You press <<17>> and receive 58 points.

Environment number 8:
The value of option 9 is 66. You have 10 choices to make in this environment.
You press <<10>> and receive 66 points.
You press <<8>> and receive 37 points.
You press <<11>> and receive 38 points.
You pr

Serial reaction time task

Data source: [52]

Number of experiments: 2
Number of participants: 238
Number of choices: 238000

Example prompt:
Press the instructed key.

Act as fast and accurately as possible.

The instruction is to press D, you press <<D>> in 1375 ms. That is correct.
The instruction is to press K, you press <<K>> in 1960 ms. That is correct.

The instruction is to press J, you press <<J>> in 1043 ms. That is correct.
The instruction is to press F, you press <<F>> in 718 ms. That is correct.
The instruction is to press D, you press <<D>> in 568 ms. That is correct.
The instruction is to press K, you press <<K>> in 527 ms. That is correct.
The instruction is to press J, you press <<J>> in 587 ms. That is correct.
The instruction is to press K, you press <<K>> in 565 ms. That is correct.
The instruction is to press J, you press <<J>> in 541 ms. That is correct.
The instruction is to press F, you press <<F>> in 631 ms. That is correct.
The instruction is to press D, you press <<D>> in 456 ms. That is correct.
The instruction is to press K, you press <<K>> in 480 ms. That is correct.
The instruction is to press J, you press <<J>> in 627 ms. That is correct.
The instruction is to press J, you press <<J>> in 1816 ms. That is correct.
The instruction is to press F, you press <<F>> in 731 ms. That is correct.
The instruction is to press D, you press <<D>> in 563 ms. That is correct.
The instruction is to press K, you press <<K>> in 401 ms. That is correct.
The instruction is to press J, you press <<J>> in 472 ms. That is correct.
The instruction is to press D, you press <<D>> in 414 ms. That is correct.
The instruction is to press K, you press <<K>> in 412 ms. That is correct.
The instruction is to press J, you press <<J>> in 439 ms. That is correct.
The instruction is to press F, you press <<F>> in 612 ms. That is correct.
The instruction is to press K, you press <<K>> in 468 ms. That is correct.
The instruction is to press J, you press <<J>> in 497 ms. That is correct.
The instruction is to press F, you press <<F>> in 397 ms. That is correct.
The instruction is to press D, you press <<D>> in 318 ms. That is correct.
The instruction is to press K, you press <<K>> in 374 ms. That is correct.
The instruction is to press J, you press <<J>> in 327 ms. That is correct.
The instruction is to press F, you press <<F>> in 604 ms. That is correct.
The instruction is to press D, you press <<D>> in 300 ms. That is correct.
The instruction is to press K, you press <<K>> in 555 ms. That is correct.
The instruction is to press J, you press <<J>> in 314 ms. That is correct.
The instruction is to press K, you press <<K>> in 441 ms. That is correct.
The instruction is to press J, you press <<J>> in 474 ms. That is correct.
The instruction is to press F, you press <<D>> in 394 ms. That is incorrect.
The instruction is to press K, you press <<K>> in 586 ms. That is correct.
The instruction is to press K, you press <<K>> in 528 ms. That is correct.
The instruction is to press J, you press <<J>> in 466 ms. That is correct.
The instruction is to press F, you press <<F>> in 434 ms. That is correct.
The instruction is to press D, you press <<D>> in 355 ms. That is correct.
The instruction is to press J, you press <<K>> in 390 ms. That is incorrect.
The instruction is to press F, you press <<F>> in 769 ms. That is correct.
The instruction is to press D, you press <<D>> in 416 ms. That is correct.
The instruction is to press K, you press <<K>> in 395 ms. That is correct.
The instruction is to press K, you press <<K>> in 601 ms. That is correct.
The instruction is to press J, you press <<J>> in 475 ms. That is correct.
The instruction is to press J, you press <<J>> in 549 ms. That is correct.

The instruction is to press F, you press <<F>> in 438 ms. That is correct.
The instruction is to press D, you press <<D>> in 327 ms. That is correct.
The instruction is to press K, you press <<K>> in 438 ms. That is correct.
The instruction is to press J, you press <<J>> in 342 ms. That is correct.
The instruction is to press F, you press <<F>> in 456 ms. That is correct.
The instruction is to press D, you press <<D>> in 380 ms. That is correct.
The instruction is to press K, you press <<K¿

Decisions from description

Data source: [53]

Number of experiments: 1
Number of participants: 1981
Number of choices: 28153

Example prompt:
You will choose from two monetary lotteries by pressing W or H.
The lotteries offer different points with different probabilities.
Your choice will trigger a random draw from the chosen lottery that will be added to your bonus.
Your goal is to maximize your bonus.
You will be presented with multiple choice problems consisting of different lotteries varying in outcomes and probabilities.

Lottery W offers 4.0 points with 80.0% probability or 0.0 points with 20.0% probability.
Lottery H offers 3.0 points with 100.0% probability.
You press <<H>>.

Lottery W offers 4.0 points with 20.0% probability or 0.0 points with 80.0% probability.
Lottery H offers 3.0 points with 25.0% probability, or 0.0 points with 75.0% probability.
You press <<H>>.

Lottery W offers -3.0 points with 100.0% probability.
Lottery H offers -32.0 points with 10.0% probability, or 0.0 points with 90.0% probability.
You press <<W>>.

Decisions from experience

Data source: [53]

Number of experiments: 79
Number of participants: 3942

Number of choices: 1015249

Example prompt:
You can sample from two monetary lotteries by pressing K or D.
The lotteries offer different points with different probabilities.
Initially, you will not know the outcomes and probabilities of the lotteries, but you can learn about them through sampling.
Whenever you sample, a random draw from the selected lottery will be generated, which does not affect your bonus.
You can sample from the lotteries in whatever order and for as long as you like.
Whenever you feel ready, you can stop sampling by pressing X and then choose one lottery for real by pressing the corresponding key.
This choice will then trigger a random draw from the chosen lottery that will be added to your bonus.
Your goal is to maximize your bonus.
You will be presented with multiple choice problems consisting of different lotteries varying in outcomes and probabilities.

You encounter a new choice problem:
You press <<K>> and observe 4.0 points.
You press <<K>> and observe 4.0 points.
You press <<K>> and observe 4.0 points.
You press <<K>> and observe 4.0 points.
You press <<K>> and observe 0.0 points.
You press <<K>> and observe 4.0 points.
You press <<K>> and observe 4.0 points.
You press <<K>> and observe 4.0 points.
You press <<K>> and observe 4.0 points.
You press <<K>> and observe 4.0 points.
You press <<K>> and observe 0.0 points.
You press <<K>> and observe 4.0 points.
You press <<D>> and observe 3.0 points.
You press <<D>> and observe 3.0 points.
You press <<D>> and observe 3.0 points.
You press <<D>> and observe 3.0 points.
You press <<D>> and observe 3.0 points.
You press <<D>> and observe 3.0 points.
You press <<D>> and observe 3.0 points.
You press <<D>> and observe 3.0 points.
You press <<D>> and observe 3.0 points.
You press <<D>> and observe 3.0 points.
You press <<D>> and observe 3.0 points.
You press <<D>> and observe 3.0 points.
You press <<D>> and observe 3.0 points.
You press <<D>> and observe 3.0 points.

You press <<D>> and observe 3.0 points.
You press <<D>> and observe 3.0 points.
You press <<K>> and observe 4.0 points.
You press <<K>> and observe 4.0 points.
You press <<K>> and observe 4.0 points.
You press <<K>> and observe 4.0 points.
You press <<K>> and observe 0.0 points.
You press <<K>> and observe 4.0 points.
You press <<X>> to stop sampling and then press <<K>>.

You encounter a new choice problem:
You press <<K>> and observe 0.0 points.
You press <<K>> and observe 0.0 points.
You press <<K>> and observe 0.0 points.
You press <<D>> and observe 0.0 points.
You press <<D>> and observe 0.0 points.
You press <<D>> and observe 0.0 points.
You press <<D>> and observe 0.0 points.
You press <<D>> and observe 3.0 points.
You press <<K>> and observe 0.0 points.
You press <<K>> and observe 0.0 points.
You press <<K>> and observe 4.0 points.
You press <<K>> and observe 0.0 points.
You press <<K>> and observe 0.0 points.
You press <<K>> and observe 0.0 points.
You press <<K>> and observe 0.0 points.
You press <<K>> and observe 4.0 points.
You press <<K>> and observe 0.0 points.
You press <<K>> and observe 4.0 points.
You press <<K>> and observe 0.0 points.
You press <<K>> and observe 0.0 points.
You press <<K>> and observe 0.0 points.
You press <<K>> and observe 0.0 points.
You press <<K>> and observe 0.0 points.
You press <<K>> and observe 0.0 points.
You press <<K>> and observe 0.0 points.
You press <<K>> and observe 0.0 points.
You press <<K>> and observe 0.0 points.
You press <<K>> and observe 0.0 points.
You press <<K>> and observe 0.0 points.
You press <<D>> and observe 0.0 points.
You press <<D>> and observe 0.0 points.
You press <<D>> and observe 0.0 points.
You press <<D>> and observe 3.0 points.
You press <<D>> and observe 0.0 points.

You press <<D>> and observe 0.0 points.
You press <<D>> and observe 0.0 points.
You press <<D>> and observe 0.0 points.
You press <<D>> and observe 0.0 points.
You press <<D>> and observe 0.0 points.
You press <<D>> and observe 3.0 points.
You press <<D>> and observe 0.0 points.
You press <<D>> and observe 0.0 points.
You press <<D>> and observe 0.0 points.
You press <<D>> and observe 0.0 points.

Changing bandit

Data source: [54]

Number of experiments: 1
Number of participants: 30
Number of choices: 141000

Example prompt:
You are participating in multiple games involving two slot machines, labeled M and V.
The two slot machines are different in different games.
Each time you choose a slot machine, you get points (choosing the same slot machine will not always give you the same points).
You select a slot machine by pressing the corresponding key.
The expected points change randomly, abruptly, and independently with a hazard rate (which you will be told).
When the points change, the new expected point value assigned to that slot machine is sampled from a uniform distribution (from 1 to 99 points).
For example, if the hazard rate is 0.1, the expected points of the machines change with 10%.
Your goal is to choose the slot machine that will give you the most points.

Game 1. The hazard rate is 0.1. There are 100 trials in this game.
You press <<M>> and get 65 points.
You press <<V>> and get 58 points.
You press <<M>> and get 65 points.
You press <<M>> and get 65 points.
You press <<V>> and get 5 points.
You press <<M>> and get 65 points.
You press <<M>> and get 65 points.
You press <<M>> and get 65 points.
You press <<V>> and get 60 points.
You press <<M>> and get 65 points.
You press <<M>> and get 65 points.

You press <<V>> and get 39 points.
You press <<M>> and get 65 points.
You press <<M>> and get 65 points.
You press <<M>> and get 65 points.
You press <<M>> and get 65 points.
You press <<V>> and get 87 points.
You press <<V>> and get 82 points.
You press <<V>> and get 82 points.
You press <<V>> and get 56 points.
You press <<M>> and get 65 points.
You press <<M>> and get 65 points.
You press <<M>> and get 65 points.
You press <<M>> and get 65 points.
You press <<V>> and get 28 points.
You press <<M>> and get 65 points.
You press <<M>> and get 65 points.
You press <<M>> and get 65 points.
You press <<M>> and get 65 points.
You press <<M>> and get 65 points.
You press <<M>> and get 65 points.
You press <<M>> and get 65 points.
You press <<M>> and get 65 points.
You press <<M>> and get 65 points.
You press <<M>> and get 65 points.
You press <<M>> and get 65 points.
You press <<M>> and get 65 points.
You press <<M>> and get 65 points.
You press <<M>> and get 10 points.
You press <<V>> and get 28 points.
You press <<V>> and get 28 points.
You press <<V>> and get 28 points.
You press <<V>> and get 28 points.
You press <<V>> and get 28 points.
You press <<V>> and get 28 points.
You press <<V>> and get 28 points.
You press <<M>> and get 13 points.
You press <<V>> and get 28 points.
You press <<V>> and get 28 points.
You press <<V>> and get 28 points.
You press <<V>> and get 28 points.
You press <<V>> and get 28 points.
You press <<M>> and get 13 points.
You press <<V>> and get 29 points.
You press <<M>> and get 88 points.
You press <<M>> and get 88 points.

You press <<M>> and get 88 points.
You press <<M>> and get 88 points.
You press <<M>> and get 88 points.
You press <<M>> and get 88 points.
You press <<V>> and get 47 points.
You press <<M>> and get 88 points.
You press <<M>> and get 53 points.
You press <<V>> and get 18 points.
You press <<M>> and get 3 points.
You press <<V>> and get 18 points.
You press <<V>> and get 18 points.
You press <<V>> and get 18 points.
You press <<V>> and get 18 points.
You press <<V>> and get 18 points.
You press <<V>> and get 18 points.
You press <<V>> and get 18 points.
You press <<M>> and get 49 points.
You press <<M>> and get 49 points.
You press <<M>> and get 49 points.
You press <<M>> and get 49 points.
You press <<M>> and get 49 points.
You press <<M>> and get 49 points.
You press <<M>> and get 49 points.
You press <<M>> and get 49 points.
You press <<V>> and get 71 points.
You press <<V>> and get 71 points.
You press <<M>> and get 68 points.
You press <<V>> and get 59 points.
You press <<M>> and get 68 points.
You press <<M>> and get 68 points.
You press <<M>> and get 68 points.
You press <<M>> and get 68 points.
You press <<M>> and get 68 points.
You press <<M>> and get 68 points.
You press <<M>> and get 68 points.
You press <<M>> and get 68 points.
You press <<M>> and get 68 points.
You press <<M>> and g

Probabilistic reasoning

Data source: [55]

Number of experiments: 2
Number of participants: 128
Number of choices: 19740

Example prompt:
You have to estimate the probability of a series of weather-related queries.
Please respond by typing your estimated probabilities.

What is the probability that the weather will be icy and not frosty on a random day in England? You estimate <<20>>%.
What is the probability that the weather will be not normal or not typical on a random day in England? You estimate <<50>>%.
What is the probability that the weather will be not frosty on a random day in England? You estimate <<65>>%.
If the weather in England is not frosty on a random day, what is the probability that the weather will also be not icy on the same day? You estimate <<40>>%.
If the weather in England is icy on a random day, what is the probability that the weather will also be not frosty on the same day? You estimate <<20>>%.
If the weather in England is typical on a random day, what is the probability that the weather will also be normal on the same day? You estimate <<50>>%.
What is the probability that the weather will be icy and frosty on a random day in England? You estimate <<35>>%.
What is the probability that the weather will be normal on a random day in England? You estimate <<50>>%.
What is the probability that the weather will be icy or frosty on a random day in England? You estimate <<35>>%.
What is the probability that the weather will be not icy or not frosty on a random day in England? You estimate <<60>>%.
If the weather in England is not typical on a random day, what is the probability that the weather will also be not normal on the same day? You estimate <<40>>%.
What is the probability that the weather will be normal and typical on a random day in England? You estimate <<30>>%.
What is the probability that the weather will be not normal on a random day in England? You estimate <<20>>%.
If the weather in England is not frosty on a random day, what is the probability that the weather will also be icy on the same day? You estimate <<20>>%.
If the weather in England is not normal on a random day, what is the probability that the weather will also be typical on the same day? You estimate <<40>>%.
If the weather in England is icy on a random day, what is the probability that the weather will also be frosty on the same day? You estimate <<60>>%.
If the weather in England is not icy on a random day, what is the probability that the weather will also be not frosty on the same day? You estimate <<60>>%.
What is the probability that the weather will be typical on a random day in England? You estimate <<70>>%.
What is the probability that the weather will be typical or not normal on a random day in England? You estimate <<50>>%.
What is the probability that the weather will be typical and not normal on a random day in England? You estimate <<30>>%.

What is the probability that the weather will be not normal and not typical on a random day in England? You estimate <<40>>%.
If the weather in England is frosty on a random day, what is the probability that the weather will also be not icy on the same day? You estimate <<60>>%.
What is the probability that the weather will be frosty or not icy on a random day in England? You estimate <<65>>%.
If the weather in England is not typical on a random day, what is the probability that the weather will also be normal on the same day? You estimate <<34>>%.
If the weather in England is normal on a random day, what is the probability that the weather will also be not typical on the same day? You estimate <<45>>%.
If the weather in England is normal on a random day, what is the probability that the weather will also be typical on the same day? You estimate <<10>>%.
What is the probability that the weather will be icy or not frosty on a random day in England? You estimate <<40>>%.
What is the probability that the weather will be frosty on a random day in England? You estimate <<40>>%.
If the weather in England is frosty on a random day, what is the probability that the weather will also be icy on the same day? You estimate <<40>>%.
If the weather in England is not icy on a random day, what is the probability that the wea

Two-step task

Data source: [56]

Number of experiments: 1
Number of participants: 139
Number of choices: 55878

Example prompt:
You are participating in a space treasure game.
In this game, you will be visiting two alien planets in search of treasure.
Each planet has two aliens on it.
The blue aliens live on the blue planet.
The red aliens live on the red planet.
When you visit a planet, you can choose an alien to trade with by pressing the corresponding button.
When you trade with an alien, it will either give you treasure or junk.
Your goal is to figure out, and trade with, the aliens that are most likely to give you treasure.
To visit a planet, you will choose one rocket ship from two by pressing the corresponding button.
They have different designations.
Each rocket ship has a planet it will fly to most of the time.
But sometimes they will take you to the other planet!
Remember the following hints:

1. How likely an alien is to give you treasure will change over time, but this change will be slow.
2. Whether you get treasure depends only on the alien you choose to trade with.
3. If there is an alien you want to trade with, remember to pick the rocket ship that is most likely to take you to that alien's planet.

You are presented with two spaceships called S and C. You press <<S>>. You end up on the blue planet. You see a blue alien named D and a blue alien named R. You press <<R>>. You find junk.
You are presented with two spaceships called S and C. You press <<S>>. You end up on the blue planet. You see a blue alien named D and a blue alien named R. You press <<D>>. You find treasure.
You are presented with two spaceships called S and C. You press <<S>>. You end up on the blue planet. You see a blue alien named D and a blue alien named R. You press <<D>>. You find junk.
You are presented with two spaceships called S and C. You press <<C>>. You end up on the red planet. You see a red alien named G and a red alien named V. You press <<V>>. You find junk.
You are presented with two spaceships called S and C. You press <<S>>. You end up on the red planet. You see a red alien named G and a red alien named V. You press <<G>>. You find treasure.
You are presented with two spaceships called S and C. You press <<S>>. You end up on the blue planet. You see a blue alien named D and a blue alien named R. You press <<D>>. You find junk.
You are presented with two spaceships called S and C. You press <<S>>. You end up on the blue planet. You see a blue alien named D and a blue alien named R. You press <<R>>. You find junk.
You are presented with two spaceships called S and C. You press <<C>>. You end up on the red planet. You see a red alien named G and a red alien named V. You press <<G>>. You find junk.
You are presented with two spaceships called S and C. You press <<C>>. You end up on the blue planet. You see a blue alien named D and a blue alien named R. You press <<D>>. You find junk.
You are presented with two spaceships called S and C. You press <<S>>. You end up on the blue planet. You see a blue alien named D and a blue alien named R. You press <<R>>. You find treasure.
You are presented with two spaceships called S and C. You press <<S>>. You end up on the red planet. You see a red alien named G and a red alien named V. You press <<V>>. You find treasure.
You are presented with two spaceships called S and C. You press <<S>>. You end up on the red planet. You see a red alien named G and a red alien named V. You press <<V>>. You find treasure.
You are presented with two spaceships called S and C. You press <<S>>. You end up on the blue planet. You see a blue alien named D and a blue alien named R. You press <<D>>. You find treasure.

You are presented with two spaceships called S and C. You press <<S>>. You end up on the blue planet. You see a blue alien named D and a blue alien named R. You press <<R>>. You find junk.
You are presented with two spaceships called S and C. You press <<S>>. You end up on the red planet. You see a red alien named G and a red alien named V. You press <<V>>. You find junk.
You are presented with two spaceships called S and C. You press <<S>>. You end up on the blue planet. You see a blue alien named D and a blue alien

Evaluation data

Two-step task (modified cover story)

Data source: [57]

Number of experiments: 1
Number of participants: 24
Number of choices: 9702

Example prompt:

You are playing the role of a musician living in a fantasy land.
You play the flute for gold coins to an audience of genies, who live inside magic lamps on Pink Mountain and Blue Mountain.
Pink Mountain has genies H and J, and Blue Mountain has genies A and E.
Each genie lives in a lamp with the corresponding letter on it.
When you arrive on a mountain, you can pick up a lamp and rub it.
If the genie is in the mood for music, he will come out of his lamp, listen to a song, and give you a gold coin.
Each genie's interest in music changes with time.
To go to the mountains, you chose one of two magic carpets, which you purchase from a magician, who enchants them to fly.
Magic carpet K generally flies to Pink Mountain, and magic carpet O generally flies to Blue Mountain.
However, on rare occasions a strong wind blowing from that mountain makes flying there too dangerous because the wind might blow you off the carpet.
In this case, the carpet is forced to land instead on the other mountain.
You can take a magic carpet or pick up a lamp and rub it by pressing the corresponding key.
Your goal is to get as many coins as possible over the next 201 days.

You are presented with magic carpets K and O. You press <<K>>. You end up on Pink Mountain. You see lamp H and lamp J. You rub lamp <<H>>. You receive 0 coins.
You are presented with magic carpets O and K. You press <<K>>. You end up on Pink Mountain. You see lamp H and lamp J. You rub lamp <<J>>. You receive 1

coins.
You are presented with magic carpets K and O. You press <<K>>. You end up on Pink Mountain. You see lamp H and lamp J. You rub lamp <<J>>. You receive 1 coins.
You are presented with magic carpets O and K. You press <<K>>. You end up on Pink Mountain. You see lamp H and lamp J. You rub lamp <<J>>. You receive 1 coins.
You are presented with magic carpets K and O. You press <<K>>. You end up on Pink Mountain. You see lamp H and lamp J. You rub lamp <<J>>. You receive 0 coins.
You are presented with magic carpets O and K. You press <<O>>. You end up on Blue Mountain. You see lamp A and lamp E. You rub lamp <<A>>. You receive 1 coins.
You are presented with magic carpets O and K. You press <<O>>. You end up on Blue Mountain. You see lamp A and lamp E. You rub lamp <<A>>. You receive 0 coins.
You are presented with magic carpets K and O. You press <<O>>. You end up on Blue Mountain. You see lamp A and lamp E. You rub lamp <<E>>. You receive 1 coins.
You are presented with magic carpets K and O. You press <<O>>. You end up on Blue Mountain. You see lamp A and lamp E. You rub lamp <<E>>. You receive 0 coins.
You are presented with magic carpets O and K. You press <<K>>. You end up on Pink Mountain. You see lamp H and lamp J. You rub lamp <<J>>. You receive 0 coins.
You are presented with magic carpets O and K. You press <<K>>. You end up on Pink Mountain. You see lamp H and lamp J. You rub lamp <<H>>. You receive 0 coins.
You are presented with magic carpets O and K. You press <<O>>. You end up on Blue Mountain. You see lamp A and lamp E. You rub lamp <<A>>. You receive 1 coins.
You are presented with magic carpets K and O. You press <<O>>. You end up on Pink Mountain. You see lamp H and lamp J. You rub lamp <<J>>. You receive 1 coins.
You are presented with magic carpets K and O. You press <<O>>. You end up on Blue Mountain. You see lamp A and lamp E. You rub lamp <<A>>. You receive 0 coins.
You are presented with magic carpets O and K. You press <<K>>. You end up on Blue Mountain. You see lamp A and lamp E. You rub lamp <<A>>. You receive 1 coins.
You are presented with magic carpets K and O. You press <<O>>. You end up on Pink Mountain. You see lamp H and lamp J. You rub lamp <<J>>. You receive 0 coins.
You are presented with magic carpets K and O. You press <<K>>. You end up on Pink Mountain. You see lamp H and lamp J. You rub lamp <<J>>. You receive 0 coins.

You are presented with magic carpets O and K. You press <<O>>. You end up on Blue Mountain. You see lamp A and lamp E. You rub lamp <<A>>. You receive 0 coins.
You are presented with magic carpets O and K. You pr

Maggie's farm (modified problem structure)

Data source: [58]

Number of experiments: 1
Number of participants: 658
Number of choices: 921200

Example prompt:

You are participating in multiple games involving three apple trees, labeled S, F, and N.
The three apple trees are different across different games.
Each time you choose an apple tree, you get an apple of a given size.
You choose an apple tree by pressing the corresponding key.
Each apple tree tends to provide apples of about the same size on average.
Your goal is to choose the apple trees that will give you the largest apples across the experiment.
The first few trials in each game are instructed trials where you will be told which apple tree to choose.
After these instructed trials, you will have the freedom to choose for either 1 or 6 trials.

Game 1. There are 8 trials in this game.
You are instructed to press F and get an apple with size 3.0 centimeters.
You are instructed to press N and get an apple with size 2.0 centimeters.
You press <<S>> and get an apple with size 4.0 centimeters.
You press <<S>> and get an apple with size 4.0 centimeters.
You press <<S>> and get an apple with size 4.0 centimeters.
You press <<F>> and get an apple with size 5.0 centimeters.
You press <<N>> and get an apple with size 5.0 centimeters.
You press <<N>> and get an apple with size 4.0 centimeters.

Game 2. There are 10 trials in this game.
You are instructed to press F and get an apple with size 9.0 centimeters.
You are instructed to press N and get an apple with size 2.0 centimeters.
You are instructed to press F and get an apple with size 10.0 centimeters.
You are instructed to press F and get an apple with size 10.0 centimeters.
You press <<F>> and get an apple with size 10.0 centimeters.
You press <<F>> and get an apple with size 8.0 centimeters.
You press <<F>> and get an apple with size 9.0 centimeters.

You press <<F>> and get an apple with size 7.0 centimeters.
You press <<F>> and get an apple with size 9.0 centimeters.
You press <<F>> and get an apple with size 10.0 centimeters.

Game 3. There are 8 trials in this game.
You are instructed to press S and get an apple with size 2.0 centimeters.
You are instructed to press F and get an apple with size 6.0 centimeters.
You press <<F>> and get an apple with size 5.0 centimeters.
You press <<F>> and get an apple with size 6.0 centimeters.
You press <<F>> and get an apple with size 6.0 centimeters.
You press <<F>> and get an apple with size 5.0 centimeters.
You press <<F>> and get an apple with size 6.0 centimeters.
You press <<F>> and get an apple with size 7.0 centimeters.

Game 4. There are 11 trials in this game.
You are instructed to press F and get an apple with size 5.0 centimeters.
You are instructed to press S and get an apple with size 6.0 centimeters.
You are instructed to press F and get an apple with size 4.0 centimeters.
You are instructed to press F and get an apple with size 4.0 centimeters.
You are instructed to press N and get an apple with size 2.0 centimeters.
You press <<S>> and get an apple with size 6.0 centimeters.
You press <<S>> and get an apple with size 5.0 centimeters.
You press <<S>> and get an apple with size 3.0 centimeters.
You press <<S>> and get an apple with size 4.0 centimeters.
You press <<N>> and get an apple with size 2.0 centimeters.
You press <<S>> and get an apple with size 4.0 centimeters.

Game 5. There are 5 trials in this game.
You are instructed to press S and get an apple with size 6.0 centimeters.
You are instructed to press F and get an apple with size 7.0 centimeters.
You are instructed to press S and get an apple with size 6.0 centimeters.
You are instructed to press S and get an apple with size 5.0 centimeters.
You press <<S>> and get an apple with size 6.0 centimeters.

Game 6. There are 11 trials in this game.
You are instructed to press S and get an apple with size 6.0 centimeters.
You are instructed to press S and get an apple with size 4.0 centimeters.
You are instructed to press S and get an apple with size 5.0 centimeters.
You are instructed to press F and get an apple with size 3.0 centimeters.
You are instructed to press N and get an apple with size 4.0 centimeters.
You press <<S>> and get an apple with size 6.0 centime

Logical reasoning (entirely novel domain)

Data source: [33]

Number of experiments: 1
Number of participants: 3543
Number of choices: 99204

Example prompt:

You're about to answer a set of 20 questions about logical reasoning. How many of the 20 questions do you think you will answer correctly?
You say <<12>>.
Compared to other participants in this study, how well do you think you will do? Marking 90% means you will do better than 90% of participants, marking 10% means you will do better than only 10%, and marking 50% means that you will perform better than half of the participants.
You say <<70>>%.
On a scale of 0 to 10, how difficult is solving logical reasoning problems for the average participant?
You say <<6>>.
On a scale of 0 to 10, how difficult is solving logical reasoning problems for you?
You say <<4>>.

You will be presented with brief passages or statements and will be required to evaluate their reasoning or determine what inferences you can logically draw from the passage.
Your task is to use the buttons D, Z, F, O, and X to select the best answer choice, even though more than one choice may present a possible answer.

Q1. Life imitates art. Which of the following, if true, most strongly supports the previous statement?
The choices are:
Z: When Warren Beatty filmed Reds, he tried to suggest not only the chaos of the Russian Revolution but also its relationship to the present.
X: The number of professional ballet companies has increased over the last five years, but the number of dance majors has decreased.
D: On Tuesday, the business section of the newspaper had predicted the drop in interest rates that occurred on Friday.
O: Truman Capote wrote In Cold Blood as a result of a series of brutal slayings by two crazed killers.
F: Soon after the advent of color television, white shirts became less popular as dressy attire for men, and pastel-colored shirts began to sell well.
You press <<F>>.

Q2. On average, federal workers receive salaries 35.5 percent higher than private-sector salaries. For instance, federal workers in California average $19,206 a year, 25 percent higher than the average pay in the private sector, which is $15,365. This information would best support which of the following opinions?
The choices are:

Z: Private-sector salaries in California are above average.
X: The private sector is being paid fairly.
O: Federal jobs are more secure than private-sector jobs.
D: Public-sector work is more difficult than private-sector work.
F: Federal pay is out of line.
You press <<Z>>.

Q3. No high jumper entered the track meet unless he or she was a track club member. No track club member both entered the meet and was a high jumper. Which of the following conclusions can be correctly drawn from the two previous sentences?
The choices are:
F: No one but high jumpers entered the meet.
D: Only track club members entered the meet.
X: No track club members entered the meet.
Z: No high jumper entered the meet.
O: Some track club members entered the meet.
You press <<Z>>.

Q4. About 33% of American men between 25 and 50 are overweight. Research has shown that in most cases men between 25 and 50 who are overweight are more subject to heart disease than men who are not overweight. Which of the following is the most logical conclusion to this argument?
The choices are:
D: Therefore, 33% of the American men between 25 and 50 should lose weight.
O: Therefore, if 33% of the American men between 25 and 50 were to lose weight, they would reduce their risk of heart disease.
X: Therefore, if the men between 25 and 50 who are overweight were to lose weight, they would reduce their risk of heart disease by 33%.
Z: Therefore, if 33% of American men were to lose weight, they would reduce their risk of heart disease.
F: Therefore, if the overweight men between 25 and 50 were to lose weight, their risk of heart disease would be reduced.
You press <<F>>.

Q5. All computer geniuses are also brilliant mathematicians. Therefore, some computer geniuses don't require calculators for simple multiplication facts. Which of the following is the least necessary assumption for the previous conclusion to be logically correct?
The choices are:
F: Some brilliant mathematicians don't require calcul

Two-step task (neural alignment)

Data source: [59]

Number of experiments: 1

Number of participants: 94
Number of choices: 28153

Example prompt:

You are playing multiple rounds of a game.
Your goal is to collect as many gold coins as possible as you visit different states.
If you are in state C, you have the choice between options B and H.
If you are in state S, you have the choice between options G and X.
Picking one of these options may result in a gold coin.
How likely an option leads to a gold coin slowly changes during the game.
Picking option O generally leads to state C, and picking option N generally leads to state S.
However, on rare occasions you will end up in the other state.
You can select an option by pressing the corresponding key.

You are presented with options O and N. You press <<N>>. You end up in state S.
You are presented with option G and option X. You press <<X>>. You receive 1 coins.
You are presented with options O and N. You press <<N>>. You end up in state S.
You are presented with option G and option X. You press <<X>>. You receive 1 coins.
You are presented with options N and O. You press <<N>>. You end up in state S.
You are presented with option G and option X. You press <<X>>. You receive 1 coins.
You are presented with options N and O. You press <<O>>. You end up in state C.
You are presented with option B and option H. You press <<B>>. You receive 1 coins.
You are presented with options N and O. You press <<O>>. You end up in state S.
You are presented with option G and option X. You press <<X>>. You receive 1 coins.
You are presented with options N and O. You press <<O>>. You end up in state C.
You are presented with option B and option H. You press <<B>>. You receive 1 coins.
You are presented with options O and N. You press <<N>>. You end up in state S.
You are presented with option G and option X. You press <<X>>. You receive 0 coins.
You are presented with options O and N. You press <<N>>. You end up in state C.
You are presented with option B and option H. You press <<H>>. You receive 1 coins.
You are presented with options N and O. You press <<O>>. You end up in state C.
You are presented with option B and option H. You press <<H>>. You receive 1 coins.
You are presented with options N and O. You press <<O>>. You end up in state S.
You are presented with option G and option X. You press <<G>>. You receive 0 coins.

You are presented with options O and N. You press <<N>>. You end up in state S. You are presented with option G and option X. You press <<X>>. You receive 1 coins.
You are presented with options N and O. You press <<O>>. You end up in state C. You are presented with option B and option H. You press <<H>>. You receive 1 coins.
You are presented with options O and N. You press <<N>>. You end up in state S. You are presented with option G and option X. You press <<X>>. You receive 0 coins.
You are presented with options O and N. You press <<N>>. You end up in state S. You are presented with option G and option X. You press <<G>>. You receive 0 coins.
You are presented with options N and O. You press <<O>>. You end up in state C. You are presented with option B and option H. You press <<B>>. You receive 1 coins.
You are presented with options O and N. You press <<N>>. You end up in state S. You are presented with option G and option X. You press <<X>>. You receive 1 coins.
You are presented with options N and O. You press <<O>>. You end up in state S. You are presented with option G and option X. You press <<X>>. You receive 1 coins.
You are presented with options O and N. You press <<N>>. You end up in state S. You are presented with option G and option X. You press <<X>>. You receive 1 coins.
You are presented with options O and N. You press <<N>>. You end up in state S. You are presented with option G and option X. You press <<X>>. You receive 0 coins.
You are presented with options O and N. You press <<N>>. You end up in state C. You are presented with option B and option H. You press <<H>>. You receive 1 coins.
You are presented with options O and N. You press <<N>>. You end up in state C. You are presented with option B and option H. You press <<H>>. You receive 1 coins.
You are presented with options N and O.

Sentence reading (neural alignment)

Data source: [60]

Number of experiments: 1
Number of participants: 5
Number of choices: 0

Example prompt:

We were sitting on the couch.

Supplementary Information References

[1] Hilbig, B. E. & Moshagen, M. Generalized outcome-based strategy classification: Comparing deterministic and probabilistic choice models. *Psychonomic bulletin & review* **21**, 1431–1443 (2014).

[2] Paszke, A. *et al. PyTorch: an imperative style, high-performance deep learning library* (Curran Associates Inc., Red Hook, NY, USA, 2019).

[3] Defazio, A. *et al.* The road less scheduled (2024). URL https://arxiv.org/abs/2405.15682. 2405.15682.

[4] Nosofsky, R. M. The generalized context model: An exemplar model of classification. *Formal approaches in categorization* 18–39 (2011).

[5] Peterson, J. C., Bourgin, D. D., Agrawal, M., Reichman, D. & Griffiths, T. L. Using large-scale experiments and machine learning to discover theories of human decision-making. *Science* **372**, 1209–1214 (2021).

[6] Van den Bos, W. & McClure, S. M. Towards a general model of temporal discounting. *Journal of the experimental analysis of behavior* **99**, 58–73 (2013).

[7] Daw, N. D., Gershman, S. J., Seymour, B., Dayan, P. & Dolan, R. J. Model-based influences on humans' choices and striatal prediction errors. *Neuron* **69**, 1204–1215 (2011).

[8] Wilson, R. C., Geana, A., White, J. M., Ludvig, E. A. & Cohen, J. D. Humans use directed and random exploration to solve the explore–exploit dilemma. *Journal of experimental psychology: General* **143**, 2074 (2014).

[9] Sutton, R. S. & Barto, A. G. *Reinforcement learning: An introduction* (MIT press, 2018).

[10] Gershman, S. J. A unifying probabilistic view of associative learning. *PLoS computational biology* **11**, e1004567 (2015).

[11] Czerlinski, J., Gigerenzer, G. & Goldstein, D. G. How good are simple heuristics? (1999).

[12] Pedroni, A. *et al.* A prospect theory account of the "hot" columbia card task (2018).

[13] Hebart, M. N., Zheng, C. Y., Pereira, F. & Baker, C. I. Revealing the multidimensional mental representations of natural objects underlying human similarity judgements. *Nature human behaviour* **4**, 1173–1185 (2020).

[14] Tomov, M. S., Schulz, E. & Gershman, S. J. Multi-task reinforcement learning in humans. *Nature Human Behaviour* **5**, 764–773 (2021).

[15] Wu, C. M., Schulz, E., Speekenbrink, M., Nelson, J. D. & Meder, B. Generalization guides human exploration in vast decision spaces. *Nature human behaviour* **2**, 915–924 (2018).

[16] Newell, A. *Unified Theories of Cognition* (Harvard University Press, Cambridge, 1990).

[17] Anderson, J. R. & Lebiere, C. The newell test for a theory of cognition. *Behavioral and brain Sciences* **26**, 587–601 (2003).

[18] Anderson, J. R. *The adaptive character of thought* (Erlbaum, Hillsdale, NJ, 1990).

[19] Binz, M. *et al.* Meta-learned models of cognition. *Behavioral and Brain Sciences* 1–38 (2023).

[20] Andreas, J. Goldberg, Y., Kozareva, Z. & Zhang, Y. (eds) *Language models as agent models.* (eds Goldberg, Y., Kozareva, Z. & Zhang, Y.) *Findings of the Association for Computational Linguistics: EMNLP 2022*, 5769–5779 (Association for Computational Linguistics, Abu Dhabi, United Arab Emirates, 2022). URL https://aclanthology.org/2022.findings-emnlp.423.

[21] Badham, S. P., Sanborn, A. N. & Maylor, E. A. Deficits in category learning in older adults: Rule-based versus clustering accounts. *Psychology and Aging* **32**, 473 (2017).

[22] Bahrami, B. & Navajas, J. 4 arm bandit task dataset (2022). URL osf.io/f3t2a.

[23] Collsiöö, A., Juslin, P. & Winman, A. Is numerical information always beneficial? verbal and numerical cue-integration in additive and non-additive tasks. *Cognition* **240**, 105584 (2023).

[24] Cox, G. E., Hemmer, P., Aue, W. R. & Criss, A. H. Information and processes underlying semantic and episodic memory across tasks, items, and individuals. *Journal of Experimental Psychology: General* **147**, 545 (2018).

[25] Enkavi, A. Z. *et al.* Large-scale analysis of test–retest reliabilities of self-regulation measures. *Proceedings of the National Academy of Sciences* **116**, 5472–5477 (2019).

[26] Feng, S. F., Wang, S., Zarnescu, S. & Wilson, R. C. The dynamics of explore–exploit decisions reveal a signal-to-noise mechanism for random exploration. *Scientific reports* **11**, 3077 (2021).

[27] Flesch, T., Balaguer, J., Dekker, R., Nili, H. & Summerfield, C. Comparing continual task learning in minds and machines. *Proceedings of the National Academy of Sciences* **115**, E10313–E10322 (2018).

[28] Frey, R., Pedroni, A., Mata, R., Rieskamp, J. & Hertwig, R. Risk preference shares the psychometric structure of major psychological traits. *Science advances* **3**, e1701381 (2017).

[29] Garcia, B., Lebreton, M., Bourgeois-Gironde, S. & Palminteri, S. Experiential values are underweighted in decisions involving symbolic options. *Nature human behaviour* **7**, 611–626 (2023).

[30] Gershman, S. J. Deconstructing the human algorithms for exploration. *Cognition* **173**, 34–42 (2018).

[31] Collins, A. G., Brown, J. K., Gold, J. M., Waltz, J. A. & Frank, M. J. Working memory contributions to reinforcement learning impairments in schizophrenia. *Journal of Neuroscience* **34**, 13747–13756 (2014).

[32] Hebart, M. N. *et al.* Things-data, a multimodal collection of large-scale datasets for investigating object representations in human brain and behavior. *Elife* **12**, e82580 (2023).

[33] Jansen, R. A., Rafferty, A. N. & Griffiths, T. L. A rational model of the dunning–kruger effect supports insensitivity to evidence in low performers. *Nature Human Behaviour* **5**, 756–763 (2021).

[34] Kool, W., Cushman, F. A. & Gershman, S. J. When does model-based control pay off? *PLoS computational biology* **12**, e1005090 (2016).

[35] Kool, W., Gershman, S. J. & Cushman, F. A. Cost-benefit arbitration between multiple reinforcement-learning systems. *Psychological science* **28**, 1321–1333 (2017).

[36] Krueger, P. M., Callaway, F., Gul, S., Griffiths, T. L. & Lieder, F. Identifying resource-rational heuristics for risky choice. *Psychological Review* (2024).

[37] Kumar, S., Dasgupta, I., Daw, N. D., Cohen, J. D. & Griffiths, T. L. Disentangling abstraction from statistical pattern matching in human and machine learning. *PLoS computational biology* **19**, e1011316 (2023).

[38] Lefebvre, G., Lebreton, M., Meyniel, F., Bourgeois-Gironde, S. & Palminteri, S. Behavioural and neural characterization of optimistic reinforcement learning. *Nature Human Behaviour* **1**, 0067 (2017).

[39] Levering, K. R., Conaway, N. & Kurtz, K. J. Revisiting the linear separability constraint: New implications for theories of human category learning. *Memory & cognition* **48**, 335–347 (2020).

[40] Ludwig, T., Siegel, M. & Schulz, E. Human multi-task learning: the why and what (2023). URL http://dx.doi.org/10.32470/CCN.2023.1528-0.

[41] Plonsky, O., Apel, R., Erev, I., Ert, E. & Tennenholtz, M. When and how can social scientists add value to data scientists? a choice prediction competition for human decision making. *Unpublished Manuscript* (2018).

[42] Popov, V. & Dames, H. Intent matters: Resolving the intentional versus incidental learning paradox in episodic long-term memory. *Journal of Experimental Psychology: General* **152**, 268 (2023).

[43] Ruggeri, K. *et al.* The globalizability of temporal discounting. *Nature Human Behaviour* **6**, 1386–1397 (2022).

[44] Sadeghiyeh, H. *et al.* Temporal discounting correlates with directed exploration but not with random exploration. *Scientific reports* **10**, 4020 (2020).

[45] Schulz, E., Franklin, N. T. & Gershman, S. J. Finding structure in multi-armed bandits. *Cognitive psychology* **119**, 101261 (2020).

[46] Somerville, L. H. *et al.* Charting the expansion of strategic exploratory behavior during adolescence. *Journal of experimental psychology: general* **146**, 155 (2017).

[47] Speekenbrink, M., Channon, S. & Shanks, D. R. Learning strategies in amnesia. *Neuroscience & Biobehavioral Reviews* **32**, 292–310 (2008).

[48] Steingroever, H. *et al.* Data from 617 healthy participants performing the iowa gambling task: A "many labs" collaboration. *Journal of Open Psychology Data* **3**, e5–e5 (2015).

[49] Tomov, M. S., Yagati, S., Kumar, A., Yang, W. & Gershman, S. J. Discovery of hierarchical representations for efficient planning. *PLoS computational biology* **16**, e1007594 (2020).

[50] Waltz, J. A., Wilson, R. C., Albrecht, M. A., Frank, M. J. & Gold, J. M. Differential effects of psychotic illness on directed and random exploration. *Computational Psychiatry (Cambridge, Mass.)* **4**, 18 (2020).

[51] Wise, T., Michely, J., Dayan, P. & Dolan, R. J. A computational account of threat-related attentional bias. *PLoS computational biology* **15**, e1007341 (2019).

[52] Wu, S., Éltető, N., Dasgupta, I. & Schulz, E. Chunking as a rational solution to the speed–accuracy trade-off in a serial reaction time task. *Scientific reports* **13**, 7680 (2023).

[53] Wulff, D. U., Mergenthaler-Canseco, M. & Hertwig, R. A meta-analytic review of two modes of learning and the description-experience gap. *Psychological bulletin* **144**, 140 (2018).

[54] Xiong, H.-D., Ji-An, L., Mattar, M. & Wilson, R. Neural network modeling reveals diverse human exploration behaviors via state space analysis (2023).

[55] Zhu, J.-Q., Sanborn, A. N. & Chater, N. The bayesian sampler: Generic bayesian inference causes incoherence in human probability judgments. *Psychological review* **127**, 719 (2020).

[56] Zorowitz, S. & Niv, Y. Data from two-step task pilots (2023). URL osf.io/2tgjd.

[57] Feher da Silva, C. & Hare, T. A. Humans primarily use model-based inference in the two-stage task. *Nature Human Behaviour* **4**, 1053–1066 (2020).

[58] Dubois, M. & Hauser, T. U. Value-free random exploration is linked to impulsivity. *Nature Communications* **13**, 4542 (2022).

[59] Feher da Silva, C., Lombardi, G., Edelson, M. & Hare, T. A. Rethinking model-based and model-free influences on mental effort and striatal prediction errors. *Nature Human Behaviour* **7**, 956–969 (2023).

[60] Tuckute, G. *et al.* Driving and suppressing the human language network using large language models. *Nature Human Behaviour* **8**, 544–561 (2024).
