# Supplementary material for: A foundation model to predict and capture human cognition
Source: Nature. 2025 Jul 2;644(8078):1002–9. doi: 10.1038/s41586-025-09215-4 (PMC12390832; doi:10.1038/s41586-025-09215-4)
Supplement: Supplementary file 2 — Reporting Summary [file 41586_2025_9215_MOESM2_ESM.pdf]

nature portfolio

Corresponding author(s): Marcel Binz

Last updated by author(s): Apr 27, 2025

Reporting Summary

Nature Portfolio wishes to improve the reproducibility of the work that we publish. This form provides structure for consistency and transparency in reporting. For further information on Nature Portfolio policies, see our Editorial Policies and the Editorial Policy Checklist.

Statistics

For all statistical analyses, confirm that the following items are present in the figure legend, table legend, main text, or Methods section.

n/a	Confirmed	
☐	☒	The exact sample size (*n*) for each experimental group/condition, given as a discrete number and unit of measurement
☒	☐	A statement on whether measurements were taken from distinct samples or whether the same sample was measured repeatedly
☐	☒	The statistical test(s) used AND whether they are one- or two-sided<br>*Only common tests should be described solely by name; describe more complex techniques in the Methods section.*
☒	☐	A description of all covariates tested
☒	☐	A description of any assumptions or corrections, such as tests of normality and adjustment for multiple comparisons
☐	☒	A full description of the statistical parameters including central tendency (e.g. means) or other basic estimates (e.g. regression coefficient) AND variation (e.g. standard deviation) or associated estimates of uncertainty (e.g. confidence intervals)
☐	☒	For null hypothesis testing, the test statistic (e.g. *F*, *t*, *r*) with confidence intervals, effect sizes, degrees of freedom and *P* value noted<br>*Give P values as exact values whenever suitable.*
☒	☐	For Bayesian analysis, information on the choice of priors and Markov chain Monte Carlo settings
☒	☐	For hierarchical and complex designs, identification of the appropriate level for tests and full reporting of outcomes
☒	☐	Estimates of effect sizes (e.g. Cohen's *d*, Pearson's *r*), indicating how they were calculated

Our web collection on statistics for biologists contains articles on many of the points above.

Software and code

Policy information about availability of computer code

Data collection	No new data was collected in this study.
Data analysis	custom code on https://github.com/marcelbinz/Llama-3.1-Centaur-70B

For manuscripts utilizing custom algorithms or software that are central to the research but not yet described in published literature, software must be made available to editors and reviewers. We strongly encourage code deposition in a community repository (e.g. GitHub). See the Nature Portfolio guidelines for submitting code & software for further information.

Data

Policy information about availability of data

All manuscripts must include a data availability statement. This statement should provide the following information, where applicable:

- Accession codes, unique identifiers, or web links for publicly available datasets
- A description of any restrictions on data availability
- For clinical datasets or third party data, please ensure that the statement adheres to our policy

Psych-101 is publicly available on the Huggingface platform: https://huggingface.co/datasets/marcelbinz/Psych-101. The test set is accessible under a CC-BY-ND-4.0 license via a gated repository: https://huggingface.co/datasets/marcelbinz/Psych-101-test.

Research involving human participants, their data, or biological material

Policy information about studies with human participants or human data. See also policy information about sex, gender (identity/presentation), and sexual orientation and race, ethnicity and racism.

Reporting on sex and gender	N/A
Reporting on race, ethnicity, or other socially relevant groupings	N/A
Population characteristics	N/A
Recruitment	N/A
Ethics oversight	N/A

Note that full information on the approval of the study protocol must also be provided in the manuscript.

Field-specific reporting

Please select the one below that is the best fit for your research. If you are not sure, read the appropriate sections before making your selection.

☐ Life sciences ☒ Behavioural & social sciences ☐ Ecological, evolutionary & environmental sciences

For a reference copy of the document with all sections, see nature.com/documents/nr-reporting-summary-flat.pdf

Behavioural & social sciences study design

All studies must disclose on these points even when the disclosure is negative.

Study description	Meta-analysis
Research sample	Meta-analysis
Sampling strategy	Meta-analysis
Data collection	information available as part of the original studies
Timing	information available as part of the original studies
Data exclusions	information available as part of the original studies
Non-participation	information available as part of the original studies
Randomization	information available as part of the original studies

Reporting for specific materials, systems and methods

We require information from authors about some types of materials, experimental systems and methods used in many studies. Here, indicate whether each material, system or method listed is relevant to your study. If you are not sure if a list item applies to your research, read the appropriate section before selecting a response.

Materials & experimental systems

n/a	Involved in the study	
☒	☐	Antibodies
☒	☐	Eukaryotic cell lines
☒	☐	Palaeontology and archaeology
☒	☐	Animals and other organisms
☒	☐	Clinical data
☒	☐	Dual use research of concern
☒	☐	Plants

Methods

n/a	Involved in the study	
☒	☐	ChIP-seq
☒	☐	Flow cytometry
☐	☒	MRI-based neuroimaging

Plants

Seed stocks: N/A

Novel plant genotypes: N/A

Authentication: N/A

Magnetic resonance imaging

Experimental design

Design type: two-step task and sentence-reading task

Design specifications: see original reports:
https://www.nature.com/articles/s41562-023-01573-1
https://www.nature.com/articles/s41562-023-01783-7

Behavioral performance measures: see original reports:
https://www.nature.com/articles/s41562-023-01573-1
https://www.nature.com/articles/s41562-023-01783-7

Acquisition

Imaging type(s): functional, structural

Field strength: 3T

Sequence & imaging parameters: see original reports:
https://www.nature.com/articles/s41562-023-01573-1
https://www.nature.com/articles/s41562-023-01783-7

Area of acquisition: Whole brain

Diffusion MRI: ☐ Used ☒ Not used

Preprocessing

Preprocessing software: fMRIprep 24.0.0, SPM12 and custom MATLAB scripts

Normalization: identical to original study

Normalization template: identical to original study

Noise and artifact removal: identical to original study

Volume censoring: identical to original study

Statistical modeling & inference

Model type and settings: predictive modeling

Effect(s) tested: whether human behavior can be predicted by language model activity

Specify type of analysis: ☐ Whole brain ☐ ROI-based ☒ Both

Anatomical location(s): *Describe how anatomical locations were determined (e.g. specify whether automated labeling algorithms or probabilistic atlases were used).*

Statistic type for inference: N/A

(See Eklund et al. 2016)

Correction | N/A

Models & analysis

n/a	Involved in the study
☒	☐ Functional and/or effective connectivity
☒	☐ Graph analysis
☐	☒ Multivariate modeling or predictive analysis

Multivariate modeling and predictive analysis

independent variables: language model activity
feature extraction: internal representations were extracted from the models' residual stream and transformed using a principal component analysis. We set the number of retained components such that they explain 95% of the variance.
model, training, evaluation metrics: cross-validated linear regression, Pearson correlation
